# Effects of Nitro-Substitution on the Spectroscopic and Self-Assembly Properties of BODIPY Dyes

**DOI:** 10.1021/acsomega.4c08799

**Published:** 2025-04-07

**Authors:** Caroline Gwaro, Caroline Ndung’U, Petia Bobadova-Parvanova, Dylan Goliber, Quynh Do, Ashley R. Walker, Evan Murders, Daniel LaMaster, Frank R. Fronczek, Jayne Garno, Maria da Graça H. Vicente

**Affiliations:** † Department of Chemistry, Louisiana State University, Baton Rouge, Louisiana 70803, United States; ‡ Department of Chemistry and Fermentation Sciences, Appalachian State University, Boone, North Carolina 28608, United States; § Department of Chemistry, Talladega College, Talladega, Alabama 35160, United States

## Abstract

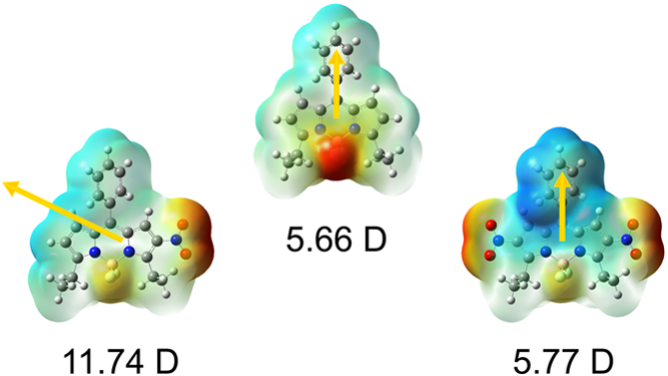

A series of boron dipyrromethene (BODIPY) dyes were nitrated in high yields using nitronium tetrafluoroborate at positions 2, 3, and 2,6 of the BODIPY core. This method allows for the regioselective nitration of the pyrrolic positions under milder conditions than previously reported methods. The photophysical properties and electronic transitions of these BODIPYs were investigated by using UV–vis spectroscopy, fluorescence spectroscopy, and density-functional theory (DFT) calculations. The introduction of one nitro group dramatically increases the dipole moment of the molecule, induces marked blue shifts in the absorption and emission bands, decreases the molar absorptivity, and increases the Stokes shifts of the BODIPYs. When a second nitro group is symmetrically introduced, the calculated dipole moments of the BODIPYs decrease in both the ground and excited states. Our studies show that the spectroscopic and self-assembly properties of nitro-substituted BODIPYs are highly dependent on solvent polarity and polarizability. In a polar organic solvent, nitro-substitution tends to quench the characteristic fluorescence of BODIPYs, while in a nonpolar solvent, significantly higher absolute fluorescence quantum yields are observed. On the other hand, aggregates are formed in aqueous solution, as observed by atomic force microscopy (AFM). Our results suggest a potential application of nitro-BODIPYs as polarity sensors.

## 1 Introduction

Boron dipyrromethene (abbreviated BODIPY) dyes are versatile fluorophores that have received great attention from researchers in the last two decades. This popularity is attributed to their broad spectrum of structure-dependent optical and chemical properties, including their strong absorptions in the UV–visible range with high molar absorptivity, sharp absorption and emission peaks, high fluorescence quantum yields, and relatively high chemical and thermal stabilities.^1−4^ For these reasons, BODIPYs have found multiple applications in biology and materials science, for example, as imaging agents,^3^ photocatalysts,^5^ photosensitizers,^6^ chemical sensors,^7^ and in molecular devices.^8^ Such diverse applications require fine-tuning of the BODIPY properties through functionalization at different core positions or by total synthesis from functionalized pyrrolic starting materials.^9,10^ The most common reactions for the functionalization of BODIPYs are electrophilic aromatic substitutions, including halogenation, nitration, sulfonation, and formylation.^11,12^ While many substituents have been installed at different positions using these reactions, the nitration of BODIPY dyes has lagged behind, as indicated by the small handful of papers published on BODIPY nitration. Nevertheless, nitro-substituted chromophores are desirable targets due to their unique electronic and optical properties and the easy reduction of the nitro group to an amine that can be further derivatized. Due to its strong electron-withdrawing ability, the nitro group controls the electronic states of π-conjugated systems, often inducing intramolecular charge transfer (ICT) processes. In addition to nitro groups, other strong electron-withdrawing substituents including CN and CF_3_ have been introduced onto the BODIPY core, and the resulting BODIPYs have shown enhanced stabilities and applications in diverse areas.^13−15^ Among the strong electron-withdrawing groups, the nitro group is particularly easy to introduce onto the BODIPY core under mild conditions and good yields, as we describe below.

Direct nitration of the 2- or 2,6-positions of BODIPYs has been previously achieved using nitric acid or cupric nitrate.^12,16,17^ Nitration using nitric acid in acetic anhydride at 0 °C gave the corresponding mononitro-BODIPYs in low to moderate yields due to the harsh reaction conditions.^16^ The use of an excess (5 equiv) of Cu(NO_3_)_2_•3H_2_O in dry solvents on a fully alkyl-substituted BODIPY gave the corresponding 3-nitro derivative in moderate yield due to the formation of several byproducts.^17^ More recently, we developed an alternative method for the mononitration of 8-pyridyl-BODIPY fluorophores in high yield using nitronium tetrafluoroborate (NO_2_BF_4_) at room temperature.^18^ Nitration of aromatic compounds using stable nitronium salts in organic solvents has been previously explored for many compounds, including porphyrin derivatives,^19^ but not for BODIPYs. Our previous studies revealed that the installation of a single nitro group at the 2-position induces large HOMO–LUMO gaps, fluorescence quenching, and increases the molecular dipole moment due to the strong electron-withdrawing effect of the nitro group.^18^ Furthermore, it was recently reported that aza-BODIPYs bearing one or two nitro groups at the 2,6-positions show higher photostability and larger Stokes shifts compared with the non-nitrated analogues.^20^ Herein, we report the synthesis of a new series of nitro-substituted BODIPY derivatives bearing the nitro group(s) at positions 2, 3, or 2,6 of the BODIPY core and discuss their unique structural, spectroscopic, electronic, and self-assembly properties.

## 2 Results and Discussion

### 2.1 Synthesis

**Scheme 1:**
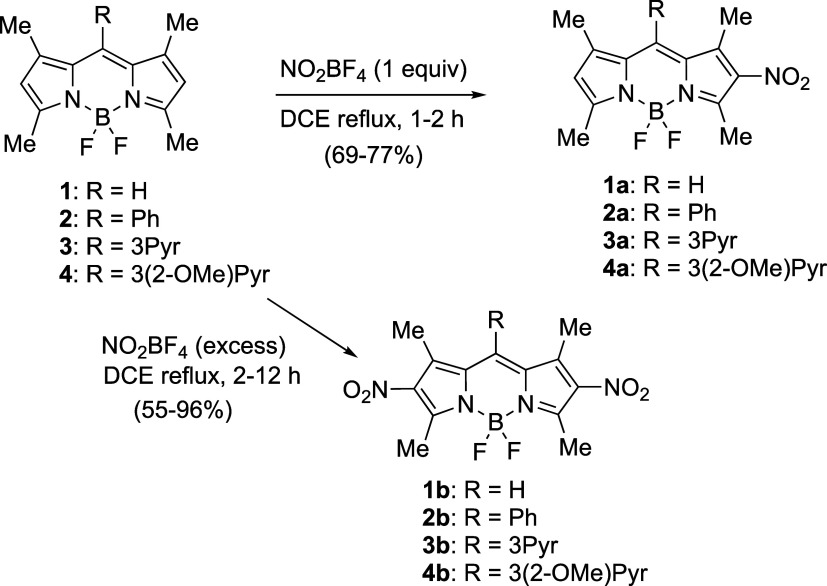
Nitration of BODIPYs **1**–**4** to Give the 2-Mono and 2,6-Dinitro Derivatives

We recently reported that 8(*meso*)-pyridyl-BODIPYs can be readily nitrated at the 2-position using NO_2_BF_4_ in dichloromethane (DCM) at room temperature in good yields.^18^ Under similar conditions, using dichloroethane (DCE) as the solvent in place of DCM, which allows refluxing at higher temperatures and results in higher product yields, BODIPYs **1**,^21^**2**,^22^**3**,^23^ and the new BODIPY **4** were nitrated at the 2- or 2,6-positions, depending on the number of equivalents of nitrating agent used. Mononitro derivatives **1a**–**4a** were isolated as the main product in good yields when BODIPYs **1**–**4** were treated with 1 equiv of NO_2_BF_4_ in refluxing DCE for up to 2 h, as shown in Scheme 1. On the other hand, 2,6-dinitro-BODIPYs **1b**–**4b** were the only product isolated when an excess of NO_2_BF_4_ was used (2 to 15 equiv) in refluxing DCE. Under these conditions, 8(*meso*)-nitration was not observed in the case of BODIPY **1**.

**Scheme 2:**
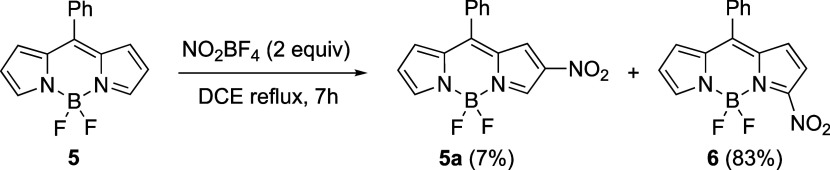
Nitration of 8-Phenyl-BODIPY **5** to Give the Mononitro Derivatives **5a** and **6**

To investigate the effect of nitration at the 3-position, the readily available *meso*-phenyl-BODIPY **5**(24) was treated with 2 equiv of NO_2_BF under the same conditions to give a mixture of 2-nitro (minor) and 3-nitro (major) BODIPYs **5a** and **6**. The 3-nitro-BODIPY **6** was obtained as the main product in 83% yield, while the 2-nitro derivative **5a** was obtained in only 7% yield, as shown in Scheme 2. Under these conditions, no nitration was observed at the 1- or 7-position, and no dinitro products were obtained. In agreement with these results, the nitration of BODIPYs bearing unsubstituted pyrrole rings using an excess of nitric acid in acetic anhydride at 0 °C is reported to give a mixture of the corresponding 2-nitro and 3-nitro derivatives in 6–11 and 36–37% yields, respectively.^16^ Since the 2,6-positions of BODIPY **5** bear the lowest molecular electrostatic potentials (MESPs, see the Supporting Information, Figure S1a),^25^ it was expected that the 2-position would be the most susceptible to electrophilic attack by the nitronium ion. However, the major product of the reaction is the 3-nitro derivative; this is consistent with a radical NO_2_· mechanism originated by single electron transfer from the BODIPY to NO_2_^+^, as previously suggested in the literature.^26^ The MESP of the cation radical BODIPY **5cat** (see the Supporting Information, Figure S1b) is least negative at position 3, suggesting that the radical anion preferentially reacts at this position.

**Scheme 3:**
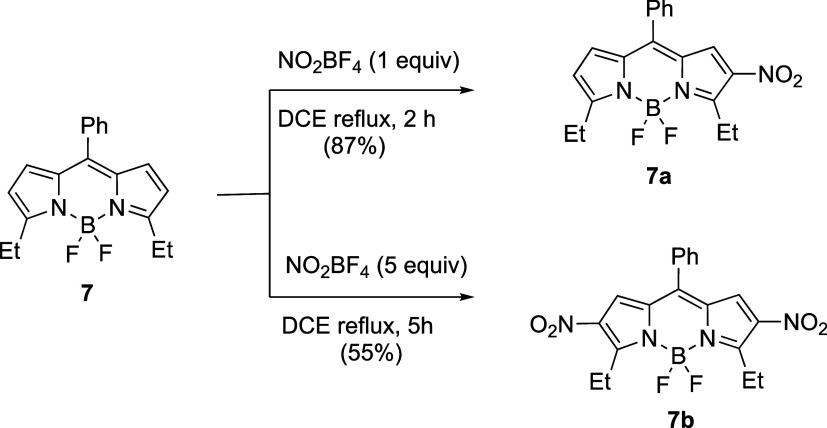
Nitration of 3,5-Diethyl-8-Phenyl-BODIPY to Give **7a** and **7b**

Blocking the 3,5-positions of the BODIPY with electron-donating ethyl groups, as in BODIPY **7**,^27^ allowed the preparation of both 2-nitro and 2,6-dinitro derivatives **7a** and **7b**, as shown in Scheme 3. The mononitro-BODIPY **7a** was prepared from BODIPY **7** in 87% yield using 1 equiv of NO_2_BF_4_ after 2 h refluxing in DCE, while an excess of NO_2_BF_4_ gave the 2,6-dinitro derivative **7b** in 55% yield after 5 h refluxing. Under these conditions, the 1,7-positions were unreactive, as we have previously observed for the electrophilic chlorination of BODIPY.^28^ On the other hand, electrophilic bromination at all available positions on BODIPY **5**, including the 1,7-positions, can be achieved with a large excess of bromine.^29^

### 2.2 Structural Characterization

The new, nitrated BODIPYs were characterized by ^1^H, ^11^B, and ^13^C NMR spectroscopy and HRMS (see the Supporting Information, Figures S13–S48). As expected, the mono-2-nitro and 3-nitro BODIPY derivatives show their unsymmetric structure in the ^1^H and ^13^C NMR spectra, while the symmetric 2,6-dinitro-BODIPYs show a symmetric structure (Supporting Information, Figure S2). All the ^11^B NMR spectra displayed a characteristic triplet between 0 and 1 ppm.

**Figure 1:**
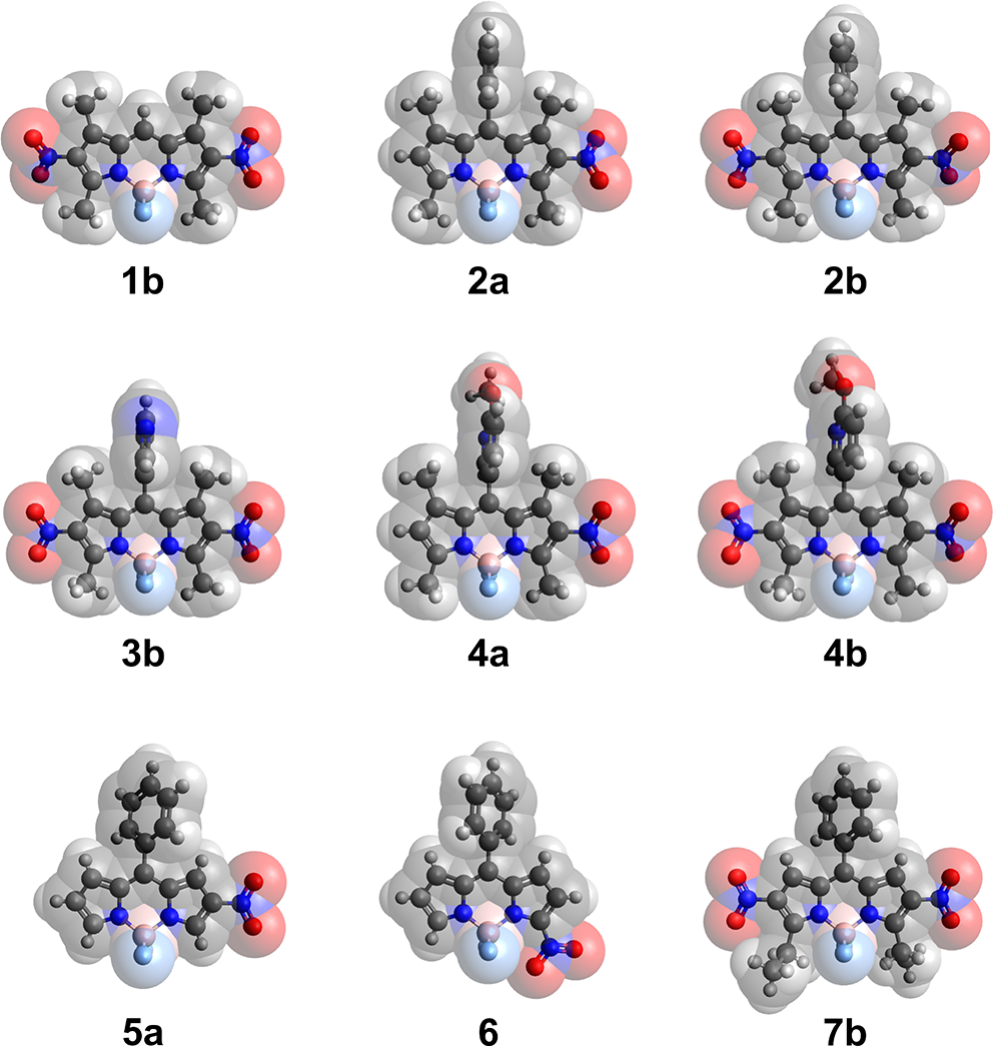
X-ray crystal structures of BODIPYs **1b**, **2a**, **2b**, **3b**, **4a**, **4b**, **5a**, **6**, and **7b** with overlaid van derWaals spheres.

The X-ray crystal structures for BODIPYs **1b**, **2a**, **2b** (two polymorphs), **3b**, **4a**, **4b, 5a, 6**, and **7b** were obtained and are shown in Figure 1. In **1b**, the BODIPY core has a slightly bowed shape, with the carbon atoms bearing the nitro groups lying 0.055 and 0.159 Å out of the plane of the central C_3_N_2_B ring. The nitro group planes form dihedral angles of 14.5 and 31.3° with the BODIPY core. In **2a**, the 12-atom BODIPY core is nearly planar with an average deviation of 0.012 Å and the nitro group has a dihedral angle of 23.6° with the core. In both polymorphs of **2b**, the BODIPY core is slightly less coplanar, having a mean deviation of 0.039 Å in one and 0.050 Å in the other. The four dihedral angles between the cores and the nitro groups are in the range 9.6–33.0°, with a mean value of 22.4°. Compound **3b** has four independent molecules, all of which exhibit some disorder in either the rotation of the nitro groups or the pyridyl group. In the most ordered molecule, the 12-atom BODIPY core is nearly planar, having a mean deviation of only 0.007 Å. Over the four molecules, the nitro groups are twisted by 2.0–30.0° out of the core planes, with a mean value of 17°. In **4a**, the BODIPY core is bowed with a mean deviation of 0.083 Å (maximum 0.176 Å for the boron atom), and the nitro group is twisted by 11.9° out of the core plane. In **4b**, the mean deviation of the BODIPY core atoms is 0.053 Å, and the nitro/core dihedral angles are 20.6 and 25.6°. In **5a**, the BODIPY core has a mean deviation of 0.044 Å from planarity, and the nitro group is nearly coplanar with the core, making a dihedral angle of only 2.8°. The crystal structure for **6** has been previously reported.^30^ In agreement with this report, the BODIPY core in **6** is slightly bowed, with its central C_3_N_2_B ring having its B atom 0.121 Å out of the plane of the other five atoms and the mean deviation of all 12 atoms 0.059 Å from coplanarity. The nitro group makes a dihedral angle of 8.9° with the core. BODIPY **7b** has two independent molecules, one of which lies on a twofold axis. The two molecules are nearly identical, with an average atomic deviation from the BODIPY core plane of 0.043 Å and core/nitro group dihedral angles ranging from 3.8 to 9.0°, with a mean of 5.4°.

### 2.3 Spectroscopic Properties

The spectroscopic properties of all BODIPYs were evaluated in two different organic solvents, acetonitrile and toluene, and in 80% water in acetonitrile. The results obtained from these studies are summarized in Table 1 and in the Supporting Information, Table S2. Their absorption and emission spectra can be found in the Supporting Information, Figures S5–S7. All BODIPYs show intense absorption maxima between 484 and 508 nm in acetonitrile and extinction coefficients ranging from 35,500 to 132,500 M^–1^·cm^–1^. The mononitro derivatives show marked hypsochromic shifts in both acetonitrile and toluene compared to the parent BODIPYs due to the strong electron-withdrawing effect of the nitro group. The observed absorption bands are due to the S_0_→S_1_ (π→π*) transition, as is characteristic for BODIPYs. In toluene compared with acetonitrile, bathochromic shifts in the order of 5–31 nm were observed in the absorption spectra for all BODIPY derivatives, as previously reported,^31,32^ due to the higher polarizability (12.4 Å^3^ for toluene vs 4.44 Å^3^ for acetonitrile^33^) and lower polarity (0.43 D for toluene vs 3.45 D for acetonitrile^33^) of toluene.

**Table 1:** Spectroscopic Properties of BODIPYs at Room Temperature in Acetonitrile and Toluene Solutions

BODIPY	acetonitrile			toluene		
	λ_abs_^max^/nm (log ε)	λ_em_^max^ /nm (Φ_f_)^a^	stokes shift/nm	λ_abs_^max^/nm (log ε)	λ_em_^max^ /nm (Φ_f_)^a^	stokes shift/nm
**1**	502 (4.98)	510 (0.76)	8	511 (4.95)	519 (0.85)	8
**1a**	486 (4.66)	502 (0.09)	16	500 (4.85)	516 (0.92)	16
**1b**	503 (4.55)	507 (0.00)	4	511 (4.99)	526 (0.83)	15
**2**	498 (4.99)	510 (0.47)	12	504 (4.97)	517 (0.57)	13
**2a**	487 (4.73)	504 (0.07)	17	498 (4.85)	513 (0.50)	15
**2b**	499 (4.76)	510 (0.00)	11	508 (5.12)	524 (0.46)	16
**3**	502 (4.97)	514 (0.43)	12	507 (4.97)	521 (0.53)	14
**3a**	491 (4.74)	507 (0.06)	16	500 (4.75)	517 (0.42)	17
**3b**	502 (4.69)	514 (0.00)	12	512 (5.02)	528 (0.34)	16
**4**	501 (4.95)	513 (0.44)	12	507 (4.97)	520 (0.57)	13
**4a**	491 (4.69)	507 (0.10)	16	500 (4.86)	516 (0.40)	16
**4b**	502 (4.73)	512 (0.00)	10	511 (5.10)	528 (0.29)	17
**5**	496 (4.74)	514 (0.008)	18	504 (4.79)	523 (0.04)	19
**5a**	484 (4.64)	506 (0.004)	22	514 (4.53)	536 (0.02)	22
**6**	484 (4.38)	529 (0.09)	45	495 (4.70)	517 (0.37)	22
**7**	508 (4.91)	524 (0.10)	16	515 (4.95)	531 (0.31)	16
**7a**	493 (4.63)	513 (0.05)	20	504 (4.82)	523 (0.38)	19
**7b**	506 (5.10)	520 (0.18)	14	516 (5.09)	535 (0.89)	19

a Absolute fluorescence quantum yields (Φ_f_).

**Figure 2:**
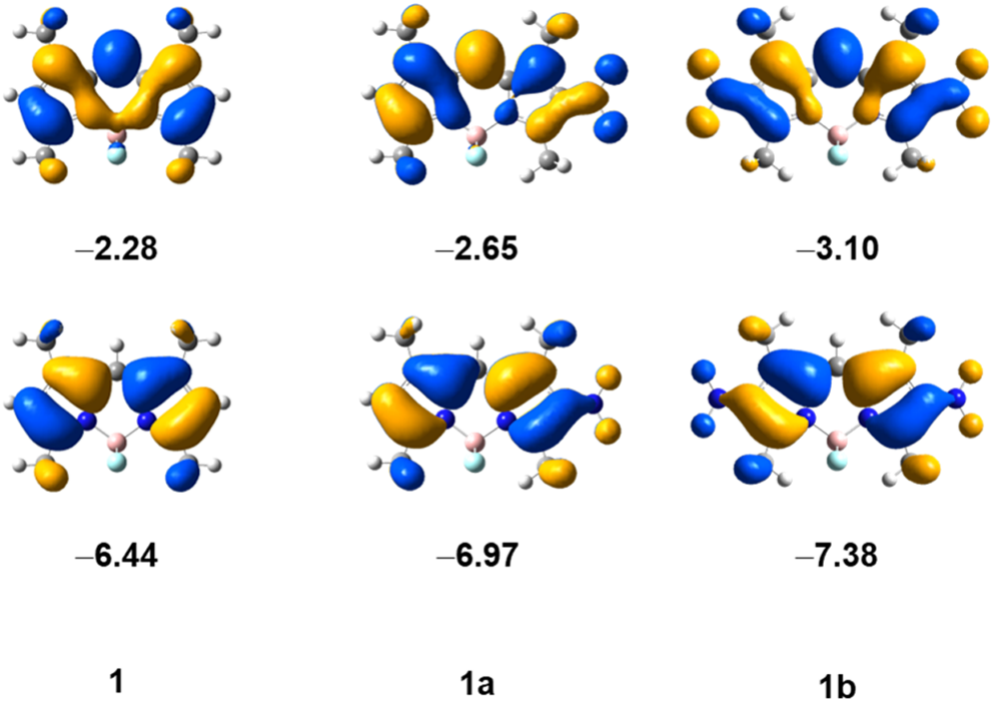
Ground state frontier molecular orbitals, HOMO (bottom) and LUMO (top), of BODIPY **1**, its 2-mononitro **1a**, and its 2,6-dinitro **1b**. Orbital energies in eV. The MO diagrams for the entire series of BODIPYs can be found in the Supporting Information, Figure S3.

The introduction of a nitro group at the 2-position, as in the case of BODIPYs **1a**–**4a**, **5a**, and **7a**, resulted in hypsochromic shifts in the absorption and emission bands, as previously observed,^16,18,20^ due to a larger HOMO–LUMO gap induced by the strong electron-withdrawing nitro group. These results were further confirmed by DFT calculations (Table 2). The mononitro substitution lowers both HOMO and LUMO (see Figure 2 and the Supporting Information, Figure S3), but the effect on HOMO is more pronounced, resulting in a larger HOMO–LUMO gap. On the other hand, the symmetric 2,6-dinitro-BODIPY derivatives displayed significant bathochromic shifts in the absorption and emission bands compared with the mononitro compounds. This is also consistent with the performed DFT calculations, the HOMO–LUMO gaps (Table 2) and energies of HOMO and LUMO (Figure 2 and Supporting Information, Figure S3). The second nitro-substitution lowers both HOMO and LUMO, but the effect on LUMO is more pronounced, resulting in a smaller HOMO–LUMO gap compared to the monosubstituted compounds.

**Table 2:** MN15/6-31+G(d,p) Calculated Spectroscopic and Molecular Properties of BODIPYs in Acetonitrile^a^

						dipole moment/D	
BODIPY	λ_abs_/nm	oscillator strength	λ_em_/nm	stokes shift/nm	HOMO–LUMO gap (eV)	S_0_	S_1_
**1**	443	0.64	503	60	4.16	5.94	5.96
**1a**	422	0.83	489	67	4.32	11.77	10.82
**1b**	429	1.03	485	56	4.28	5.77	5.84
**2**	437	0.60	492	55	4.23	6.76	6.80
**2a**	424	0.75	491	67	4.33	12.38	11.52
**2b**	435	0.90	491	56	4.26	7.97	8.07
**3**	439	0.60	500	61	4.19	5.27	5.47
**3a**	427	0.75	496	69	4.30	10.96	9.80
**3b**	419	0.78	467	48	4.41	7.67	7.88
**4**	439	0.59	496	57	4.20	5.66	5.78
**4a**	428	0.76	493	65	4.27	11.31	10.49
**4b**	437	0.88	492	55	4.24	7.29	7.28
**5**	427	0.49	481	54	4.32	7.90	8.12
**5a**	412	0.62	471	59	4.45	12.69	12.17
**6**	437	0.47	519	82	4.26	15.11	14.96
**7**	444	0.57	498	54	4.17	5.66	5.83
**7a**	426	0.72	491	65	4.30	11.74	10.74
**7b**	439	0.86	490	51	4.23	5.77	5.79

a The dominant transition for all the compounds is S_0_→S_1_.

An increase in the Stokes shifts in the order of ca. 4–8 nm was observed for the mononitro-BODIPYs compared to the parent compounds (Table 1). All DFT methods used (ωB97X-D, M06-2X, MN15, and TPSSh) overestimate the Stokes shifts of the BODIPYs and their nitrated derivatives; however, the larger shift of the mononitro-BODIPY compared to the unsubstituted compound is consistent throughout the series, regardless of the DFT functional used. The larger Stokes shifts observed for the mononitro-BODIPYs are likely due to larger structural differences between the ground and excited states compared to the parent compounds. Indeed, MN15/6-31+G(d,p) calculations of the **1**, **1a**, **1b**, **2**, **2a**, and **2b** and the **7**, **7a**, and **7b** series demonstrate that, upon excitation, the B–N bonds shorten, while the *meso*-C–C bonds elongate (Table S1, Supporting Information). However, in the case of mononitro **1a**, **2a**, and **7a**, the effect is considerably more significant. It is also interesting to note that while the excited states of **1** and **1b**, **2** and **2b**, and **7** and **7b** remain relatively symmetric, the excited states of **1a**, **2a**, and **7a** become even less symmetric than the ground state; this effect is more pronounced on the side of the molecule opposite of the nitro-substituent. Such structural changes upon excitation might explain the experimentally observed larger Stokes shifts for the mononitro-BODIPYs.

The shapes of the frontier molecular orbitals (MOs) for the BODIPY **1** series are shown in Figure 2 as an example; the MO diagrams for the entire series of BODIPYs can be found in the Supporting Information, Figure S3. The shapes of the frontier MOs clearly show the effect of the electron-withdrawing nitro group(s). Although the electron density is delocalized onto the nitro groups, it is also still located in the plane of the BODIPY core, indicating significant fluorescence activity, consistent with the experimental observations.

As expected, BODIPYs **5** and **7** show a pronounced decrease in fluorescence quantum yields relative to BODIPYs **1**–**4** due to the absence of the 1,7-methyl substituents in **5** and **7**. The absence of 1,7 groups on the BODIPY core allow for free rotation of the 8(*meso*)-phenyl group, therefore increasing the rate of nonradiative decay processes. In general, the nitrated BODIPYs showed lower fluorescence quantum yields compared with their unsubstituted parent BODIPYs caused by the nonradiative decay pathways introduced by the nitro groups. However, there are exceptions in the case of BODIPYs **1a** and **7a** in toluene, which are still under investigation; the trend in the molecular energies of each series may shed some light on this result. The decrease in molecular energy upon mononitration is consistent for **1**, **5**, and **7** as well as for **2**–**4**, with the former having an average of 0.043 eV lower energy. We hypothesize that the cause of this stabilization could affect the excited state behavior, reducing the nonradiative decay pathways, resulting in the inconsistent decrease in the fluorescent quantum yields. It should be noted that for **5a**, this reduction does not appear to be enough to overcome the pre-existing pathways. We also observed an enhancement in the fluorescence of the 3-nitro BODIPY **6** relative to the nearly nonfluorescent BODIPY **5**, as previously observed.^16^ This result is attributed to the strong electron-withdrawing ability of the nitro group, which reduces the effect of the *meso*-phenyl free rotation that leads to nonradiative pathways and fluorescence quenching. This result is in agreement with the much lower fluorescence quantum yield observed for the 2-formyl derivative of BODIPY **5** compared with its 3-formyl analogue.^34^

As expected, the introduction of a single strong electron-withdrawing nitro group significantly increases the theoretically calculated dipole moments of the BODIPYs, for example, from 5.94 D in BODIPY **1** to 11.77 D in **1a**, as shown in Table 2. When a second nitro group is symmetrically introduced into the BODIPY core, as in 2,6-dinitro-BODIPYs **1b**–**4b** and **7b**, the calculated dipole moment of the molecule decreases compared with the mononitro derivatives, becoming closer to that of the parent molecule. In the case of BODIPY **1b**, the dipole moment decreases to 5.77 D. The lower polarity of the 2,6-dinitro BODIPYs was also indicated by their lower r_f_ values on thin-layer chromatography (TLC), compared with the corresponding 2-mononitro-BODIPYs. Nitration of the BODIPY core also changes the orientation of the dipole moment, as shown in the Supporting Information, Figure S2, for BODIPYs **1**, **1a**, and **1b**, as an example. While in the symmetrically substituted BODIPYs, the vector is along the pseudo-*C*_2_ axis of symmetry (all compounds were optimized without symmetry constraints), in the case of the 2-mononitro-BODIPYs, it is oriented at approximately 60° to this pseudo-axis. Similar orientations are observed for the entire series of compounds. The orientation of the dipole moment remains similar upon excitation (Figure S2). However, as can be seen in the last column of Table 2, the magnitude of the dipole moment changes. Interestingly, it increases upon excitation for the symmetric parent and 2,6-disubstituted BODIPYs but decreases for the mononitro-BODIPYs. This decrease in the dipole moment upon excitation might be related to the generally lower fluorescence of mononitro-BODIPYs and is consistent with a recent study from our laboratory.^35^

The absolute fluorescence quantum yields observed for all BODIPYs were always higher in toluene compared with acetonitrile, even for the non-nitrated parent BODIPY derivatives **1**–**4**, **5**, and **7**, due to their very different polarity (0.43 D for toluene vs 3.45 D for acetonitrile^33^), polarizability (12.4 Å^3^ for toluene vs 4.44 Å^3^ for acetonitrile^33^), dielectric constant (ε=2.38 for toluene vs ε=37.5 in acetonitrile^33^), and consequentially their solvation ability. All of these solvent properties have been found to influence the fluorescence emission properties of BODIPYs; in particular, the dependence of fluorescence quantum yields with the solvent polarity has been previously observed^32,31,36−38^ and can be due to the increased rate of nonradiative deactivation.

Most strikingly, although the dinitro-BODIPY **1b** is nonfluorescent in acetonitrile, the absolute fluorescence quantum yield measured in toluene was 0.83. This fluorescence quenching effect might be due to the specific solvation of this compound in polar acetonitrile, as previously observed.^36−38^ The significantly higher dipole moment of nitro-substituted BODIPYs likely results in stronger solvation in the more polar solvent. Similarly, fluorescence quenching was also observed in aqueous solutions due not only to the polarity of the solvent but also to the formation of aggregates, as indicated by UV–vis spectroscopy (see Supporting Information Figure S7). The aggregates were imaged using atomic force microscopy (AFM), as described below.

### 2.4 AFM Studies

**Figure 3:**
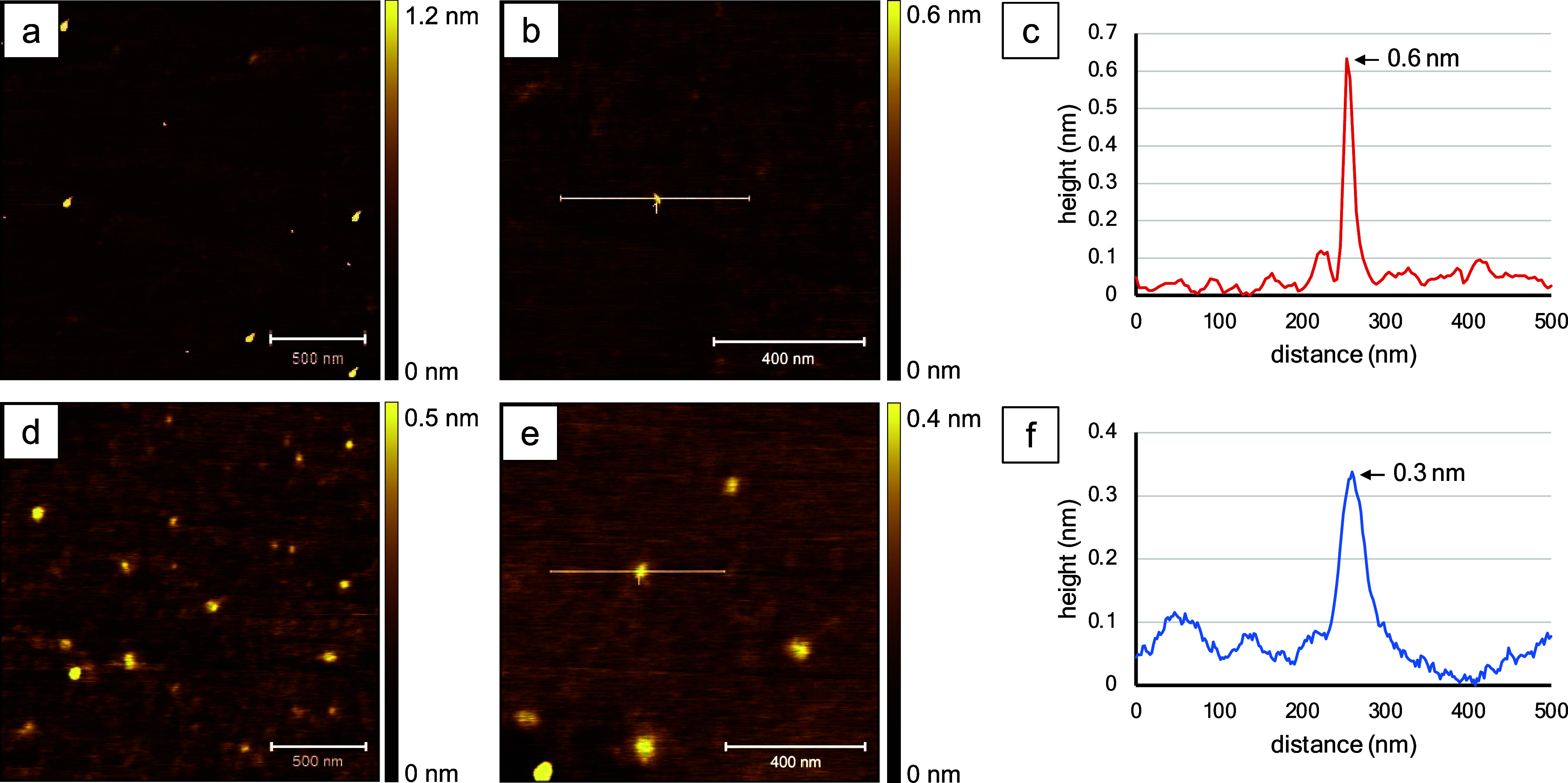
Topography images of BODIPY **1b** acquired with tapping-mode AFM in ambient air. Samples shown in the top panel were dissolved in acetonitrile, and those in the bottom panels were prepared in toluene. (a) Topography frame, 2 × 2 μm^2^; (b) zoom-in view, 1 × 1 μm^2^; (c) cursor profile for the line in (b); (d) image size 2 × 2 μm^2^; (e) close-up view, 1 × 1 μm^2^; and (f) cursor profile for the line in (e).

**Figure 4:**
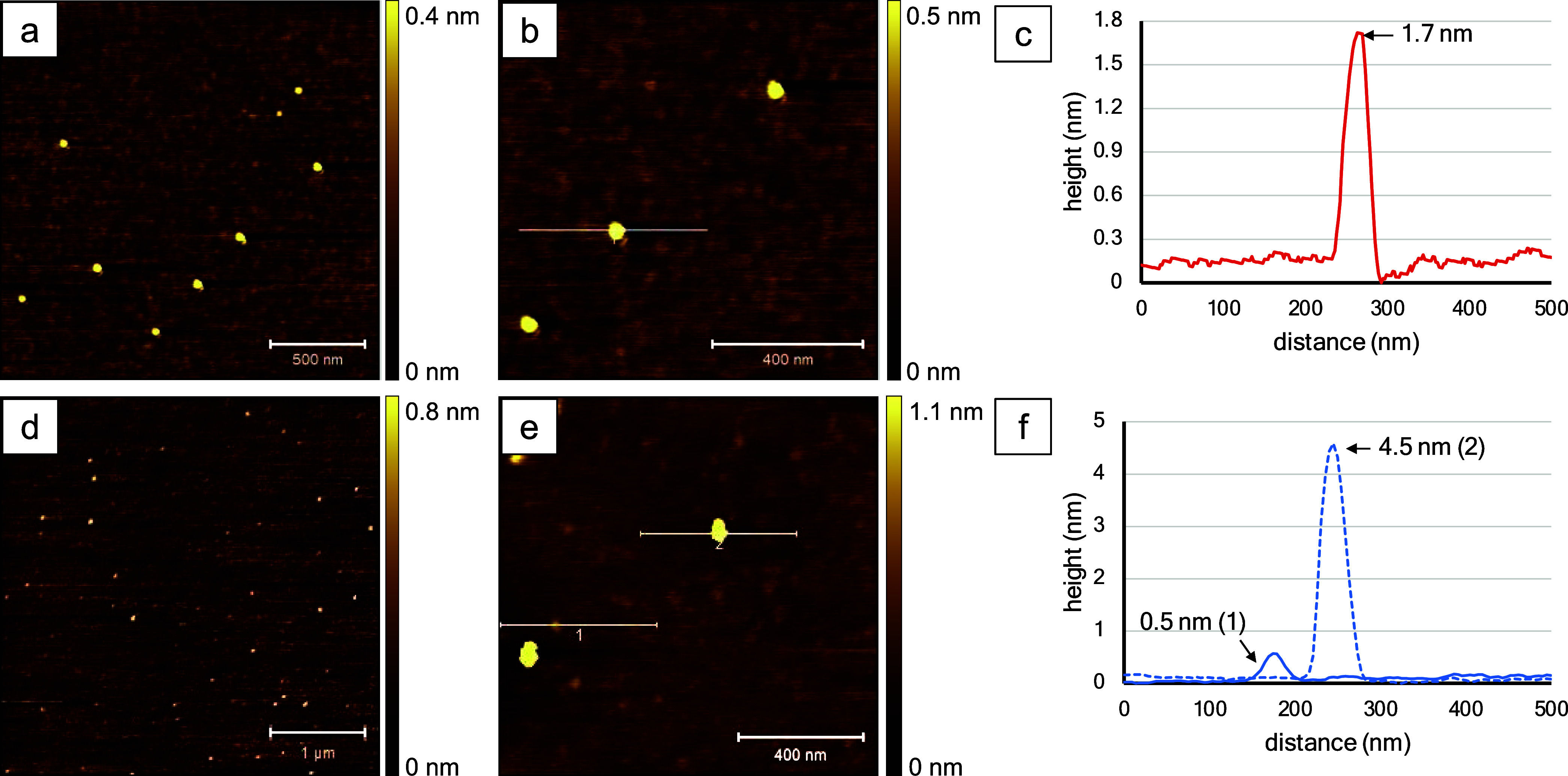
Topography AFM results for BODIPY **2b** dissolved in acetonitrile (top row) and toluene (bottom row); the images were captured using tapping-mode AFM in ambient conditions. (a) Topography frame, 2 × 2 μm^2^; (b) zoom-in view, 1 × 1 μm^2^; (c) cursor profile for the line in (b); (d) image size 4 × 4 μm^2^; (e) close-up view, 1.2 × 1.2 μm^2^; and (f) cursor profile for the line in (e).

**Figure 5:**
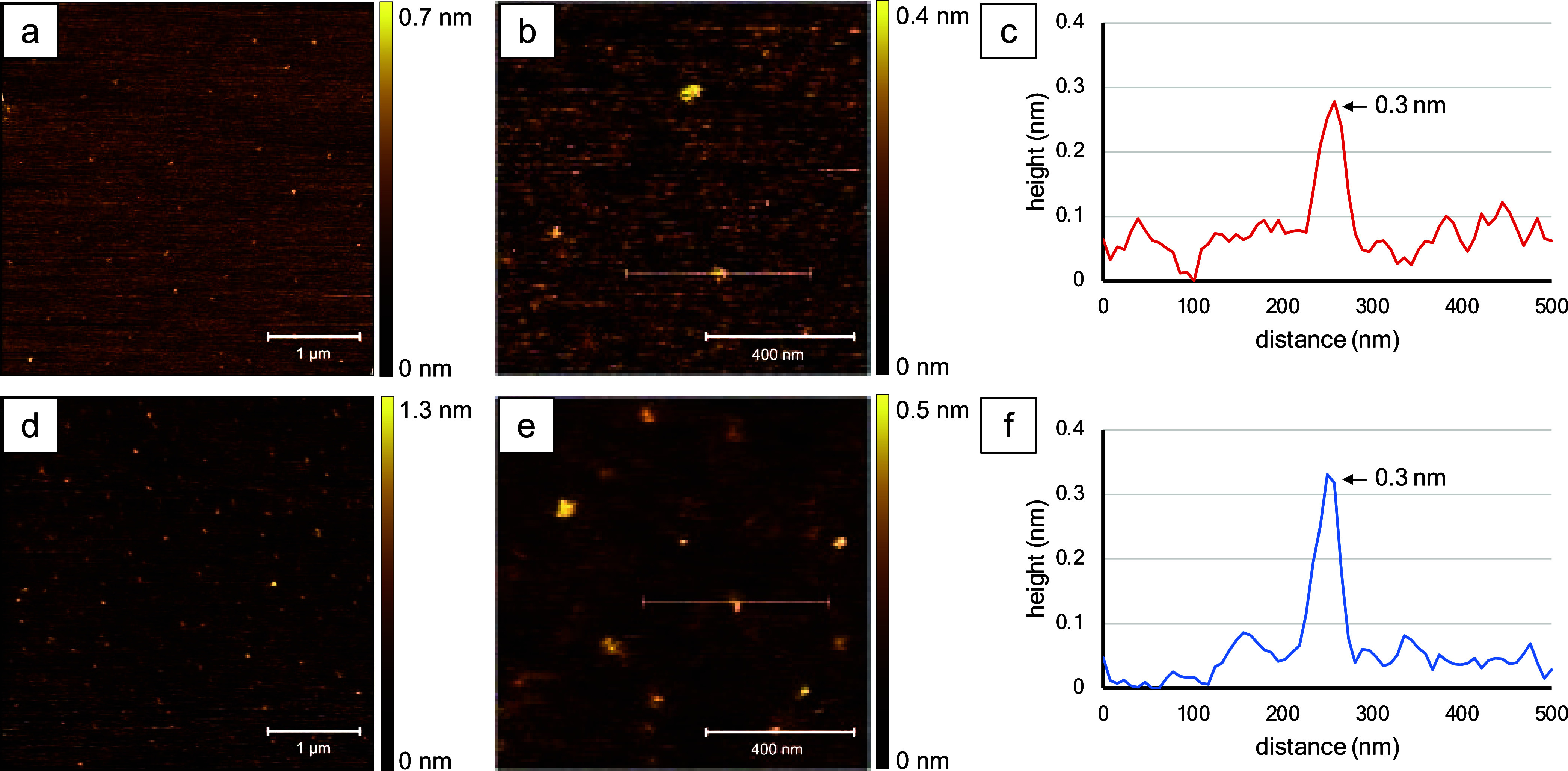
Images of BODIPY **7b** dissolved in either acetonitrile (top row) or toluene (bottom panels), captured using tapping-mode AFM. (a) Topography frame, 4 × 4 μm^2^; (b) zoom-in view, 1 × 1 μm^2^; (c) cursor profile for the line in (b); (d) image size 4 × 4 μm^2^; (e) close-up view, 1 × 1 μm^2^; and (f) cursor profile for the line in (e).

AFM topography images were captured for dropcast samples of BODIPYs **1b**, **2b**, and **7b**, dried on mica (0001). The representative AFM images are shown in Figures 3–5. At high concentrations, multilayers of the molecules were generated on flat substrates; therefore, the samples were diluted in acetonitrile or toluene to control the surface coverage and enable molecular-level characterizations with AFM.

Individual molecules of BODIPY **1b** are revealed as bright spots in the AFM topography frames (see Figure 3). A few bright features are apparent in the 2 × 2 μm^2^ frame in Figure 3a, with a surface coverage of ∼15 nanostructures. With a close-up view (Figure 3b), an individual molecule is disclosed. A profile for the white line drawn across the center of the feature indicates a height of ∼0.6 nm (Figure 3c). For the AFM cursor measurements, the heights of molecules are used for size estimates because the lateral dimensions of the AFM tip are much wider than the surface features. The *x*–*y* dimensions are exaggerated with AFM at sizes on the order of a few nanometers or less due to a convolution of the geometry of the probe and sample; however, vertical measurements on the order of tens of nanometers are not distorted by tip–sample convolution. For example, AFM measurements of the distance between molecules are accurate.

A greater surface density was observed for a sample of BODIPY **1b** prepared in toluene, as shown in Figure 3d,e. There are six nanostructures shown within the 1 × 1 μm^2^ topograph of Figure 3e. The smallest feature measured 0.3 nm in height, as shown by the cursor profile of Figure 3f. Overall, the sizes of BODIPY **1b** nanostructures prepared in toluene ranged from 0.3 to 2.0 nm, with an average size of 0.6 ± 0.3 nm (*n* = 49). Depending on the orientation of the molecule (either side-on or end-on), the dimensions for the individual nanoparticles of BODIPY **1b** measured with X-ray diffraction were 0.6–1.4 nm, which corresponds to a side-on or end-on orientation of individual molecules, respectively. The samples shown in Figure 3 do not indicate self-aggregation, and AFM images reveal nanostructures that correspond to individual molecules of BODIPY **1b** with either side-on or end-on orientations.

Samples of BODIPY **2b** dissolved in either acetonitrile or toluene were characterized with AFM, as shown in Figure 4. Nine nanostructures were observed within the 2 × 2 μm^2^ frame of Figure 4a. The dark bands on the right sides of the bright spots are artifacts introduced by digital processing. Three clusters are resolved in the zoom-in frame (1 × 1 μm^2^) of Figure 4b, which are aggregate structures of two or more molecules. The height of one of the features measured 1.7 nm (Figure 4c), which corresponds roughly to the dimensions of a cluster of two molecules. The crystallographic dimensions of BODIPY **2b** were 1.392 nm side-to-side, 1.213 nm top-to-bottom, and 0.638 nm in thickness.

For a sample of BODIPY **2b** prepared in toluene, Figure 4d (4 × 4 μm^2^ frame) discloses the surface density and distribution of nanoaggregates across a relatively broad area of the substrate. The close-up frame reveals an irregular shape of the molecular clusters (Figure 4e). Two line profiles are shown in Figure 4f. The first line profile indicates a height of 0.5 nm, which matches the thickness dimensions of a single molecule of BODIPY **2b** (Figure 4f). Line 2 was drawn across a cluster that measured 4.5 nm in height, which would correspond to the dimensions of 3–7 molecules. Although not as prominent as compared to the clusters, there is a predominance of the smaller features of BODIPY **2b**. Size analysis of the sample prepared with toluene revealed the heights of the nanostructures ranged from 0.3 to 0.9 nm, with an average size of 0.5 ± 0.1 nm (*n* = 54). The heights of the nanostructures likely correspond primarily to a side-on configuration of individual molecules of BODIPY **2b**.

The self-assembly of BODIPY **7b** was evaluated using AFM analysis for samples dissolved in acetonitrile or toluene, as compared in Figure 5. The surface coverage of individual molecules is shown for a 4 × 4 μm^2^ topography frame in Figure 5a, revealing small bright structures spaced at regular intervals of ∼0.5 nm. A close-up view of the three individual molecules is presented in Figure 5b for the sample prepared in acetonitrile. The height measured in the cursor profile was ∼0.3 nm (Figure 5c), corresponding to the smallest dimension of BODIPY **7b**. Topography images acquired for a sample of BODIPY **7b** prepared in toluene reveal similar dimensions. Higher surface coverage was obtained showing an intramolecular spacing of around 100–200 nm in the 4 × 4 μm^2^ topography frame of Figure 5d compared to 300–400 nm spacing between molecules evidenced in Figure 5a. A magnified view of the molecules is presented in Figure 5e, showing ∼12 individual molecules. One of the nanostructures measured 0.3 nm in height, as shown with a cursor profile of Figure 5f, which matches the dimensions of a single molecule of BODIPY **7b**. The average size for the sample of BODIPY **7b** prepared in toluene measured 2.1 ± 1.4 nm (*n* = 33), indicating that clusters of two or more molecules were formed.

**Figure 6:**
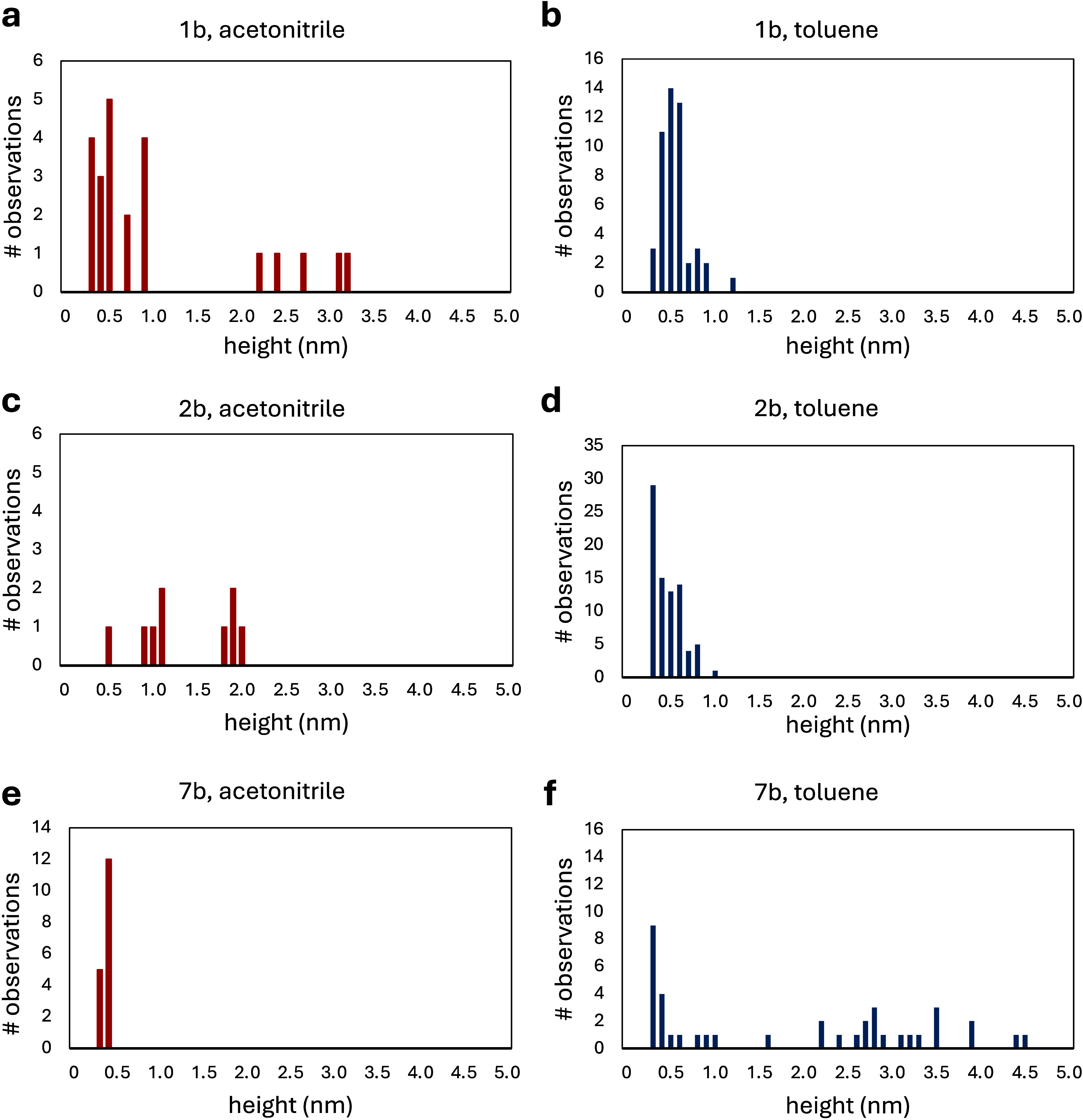
Size analysis for nanostructures of BODIPY **1b** (a,b), **2b** (c,d), and **7b** (e,f) for samples that were dissolved in acetonitrile (red) or toluene (blue), respectively. The results were obtained from AFM cursor height measurements.

To evaluate the effects of self-assembly, size analyses for the three BODIPY molecules (**1b**, **2b**, **7b**) are compared in Figure 6. The data for the histograms was obtained from individual cursor measurements of nanostructures within multiple frames for 2 × 2 μm^2^ areas. Any structures larger than 2 nm were considered to correspond to molecular clusters of two or more molecules. Interestingly, the polarity of the solvent used for sample preparation influenced the self-assembly of the BODIPY molecules. For example, BODIPY **1b** formed clusters in acetonitrile that measured between 2.2 and 3.2 nm in height (Figure 6a); however, self-assembly was not detected in nonpolar toluene since all the structures were smaller than 1.2 nm (Figure 6b). A similar trend was observed for BODIPY **2b**. In acetonitrile, aggregates were detected that measured 1.8–2.0 nm in height (Figure 6c), whereas aggregates were not observed for the sample prepared in toluene. For BODIPY **2b** dissolved in toluene, the histogram in Figure 6d shows that the majority of the nanostructures of BODIPY **2b** were smaller than 1 nm in height and were likely to be individual molecules. A different trend was evident for BODIPY **7b**. Samples prepared in acetonitrile did not display aggregation, and a uniform size of 0.3–0.4 nm was observed for the nanostructures (Figure 6e). In toluene, half of the nanostructures of BODIPY **7b** measured larger sizes ranging from 1.6 to 4.6 nm, which indicates that self-assemblies consisting of 2–7 molecules were formed when the BODIPY was dissolved in a nonpolar solvent (Figure 6f). Overall, these results demonstrate that symmetric 2,6-dinitro-substituted BODIPYs can form different types of molecular assemblies that depend on the polarity of the solvent.

**Figure 7:**
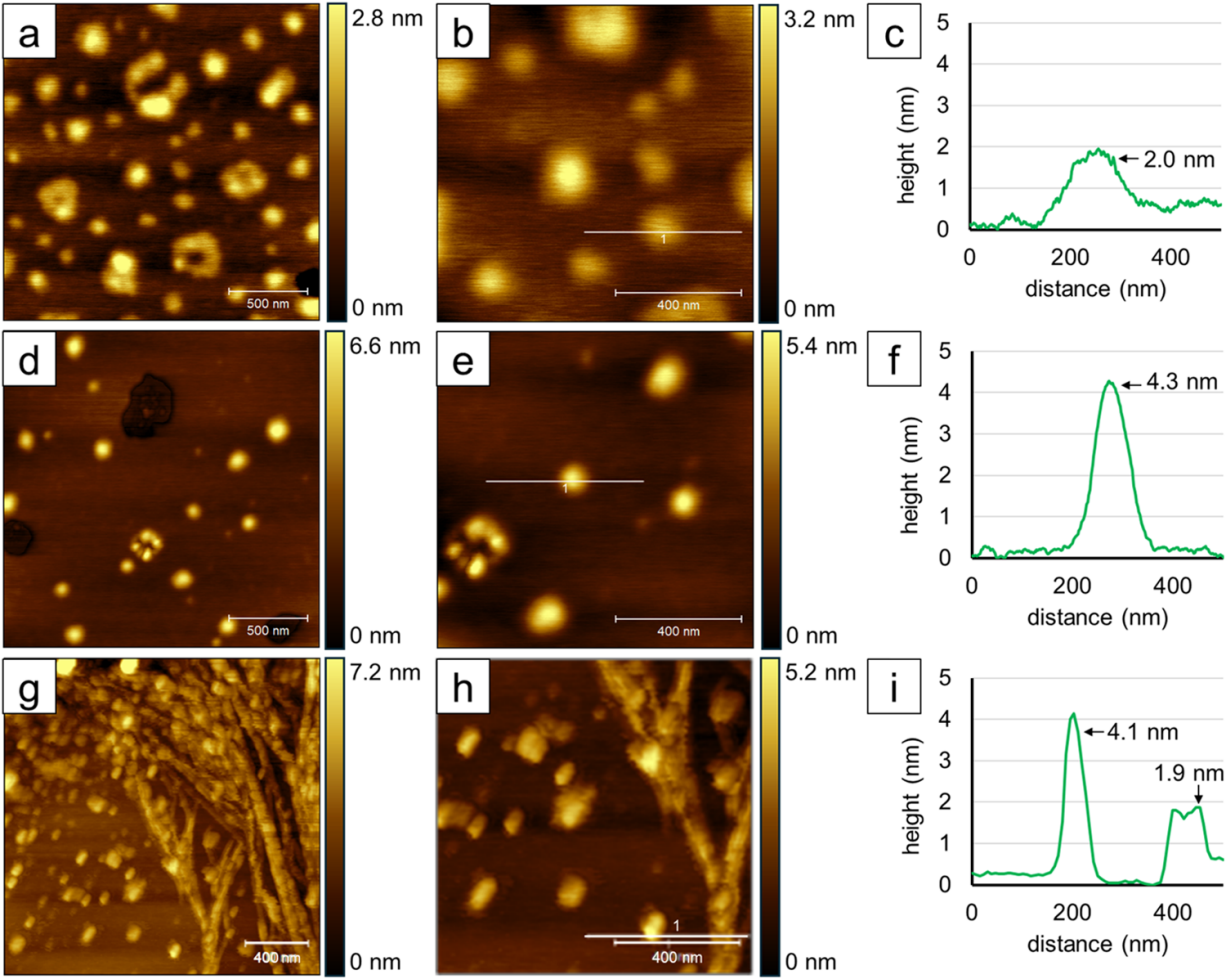
Topography AFM results for BODIPYs **1b**, **2b**, and **7b** dissolved in 80% water and 20% acetonitrile. For BODIPY **1b**, (a) 2 × 2 μm^2^ topography frame, (b) 1 × 1 μm^2^ zoom-in view, and (c) cursor profile for the line in (b). For **2b**, (d) 2 × 2 μm^2^ image, (e) 1 × 1 μm^2^ zoom-in view, and (f) cursor profile for the line in (e). For **7b**, (g) 2 × 2 μm^2^ topography frame, (h) 1 × 1 μm^2^ zoom-in view, and (i) cursor profile for the line in (h). The images were acquired using tapping-mode AFM in ambient conditions.

**Figure 8:**
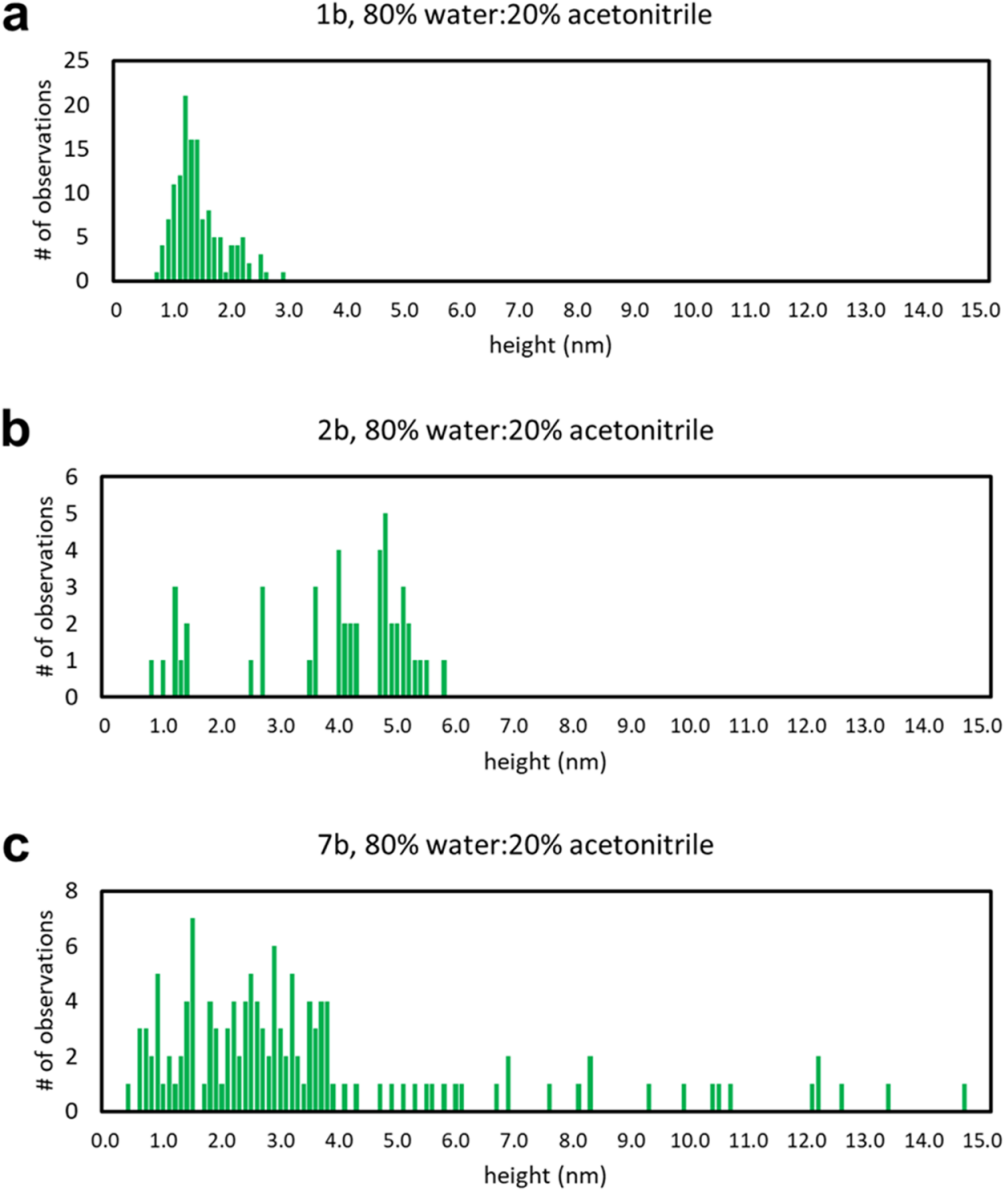
Size analysis for aggregates of BODIPY **1b, 2b**, and **7b** for samples that were dissolved in 80% water and 20% acetonitrile. The results were obtained from AFM cursor height measurements.

Additionally, the effect of solvent polarity on BODIPY aggregation was investigated using solutions of the BODIPYs in 80% water:20% acetonitrile, and the data are summarized in Figures 7 and 8 and Supporting Information Figures S8–S12. To examine the morphology of the aggregates, the amount of solution deposited onto the substrate was doubled. Aggregates of BODIPY **1b**, **2b**, and **7b** are shown in Figure 7. A 2 × 2 μm^2^ topography frame of **1b** shows around 30 round aggregates and 5 clusters of multiple aggregates forming a loose ring (Figure 7a). A 1 × 1 μm^2^ zoom-in view contains approximately ten aggregates of varying lateral sizes (Figure 7b). The height profile taken from the line in Figure 7b shows a 2.0 nm tall aggregate (Figure 7c), with an average height of 1.4 ± 0.4 nm (*n* = 134). Aggregates of BODIPY **1b** were calculated to be 0.6–2.9 nm tall. For **2b**, the 2 × 2 μm^2^ area shown in Figure 7d contains 13 round, bright spots. Figure 7e presents a 1 × 1 μm^2^ close-up of the same area, with one aggregate measuring 4.3 nm tall (Figure 7f). The average height of the aggregates was calculated to be 3.8 ± 1.4 nm (*n* = 48), with a range of 0.8–5.8 nm. Meanwhile, the aggregation of BODIPY **7b** in 80% water in acetonitrile forms smaller aggregates and a large network of strands, as seen in Figure 7g. The shape of the smaller aggregates appears more rectangular due to tip–sample convolution distorting the lateral appearance in the topography images, but the image artifacts do not affect the height measurements. Considering other topography images of the BODIPYs, the actual morphology of the aggregates was assumed to be more circular than displayed in Figure 7g,h. The 1 × 1 μm^2^ view of Figure 7g shows approximately ten small dots and a branching strand. A cursor profile shows that the small aggregate was 4.1 nm tall and the strand was 1.9 nm tall. Size analysis was performed for the small aggregates with an average height of 3.5 ± 3 nm (*n* = 131), with the heights ranging from 1.3 to 14.6 nm.

The distribution of heights for BODIPYs **1b**, **2b**, and **7b** dissolved in a solution of 80% water and 20% acetonitrile is visualized in Figure 8 for comparison purposes. All BODIPYs displayed some degree of aggregation. Compared with the other BODIPY compounds, **1b** formed the smallest and most uniform aggregates, with a difference of 2.3 nm between the smallest and largest aggregates. For **2b**, the aggregates in Figure 7d were more circular in shape and did not cluster as much as compared to the topography image in **1b** (Figure 7a). The aggregates of BODIPY **2b** were measured to be, on average, more than twice the height of **1b**, with a difference of 5.0 nm between the largest and smallest aggregates. While the similarly shaped aggregates of **7b** were of a similar average height to **2b**, the height distribution was significantly more dispersed, with the difference between the smallest and largest aggregates being 13.3 nm. In addition, neither **1b** nor **2b** aggregated into the strands seen in BODIPY **7b** (Figure 7g). More topography images in Figure S44 reveal the variance in the morphology in aggregates of **7b**, ranging from dots like **1b** and **2b** to long strands and enormous circular clusters. Across all solvents, **1b** formed consistently small aggregates. Meanwhile, **7b** formed large self-assemblies in solutions of toluene and 80% water/20% acetonitrile. A similar result was not seen in acetonitrile, possibly due to a lower sample size for the acetonitrile measurements. For BODIPY **2b**, changes in the solvent polarity appear to affect the distribution of **2b** aggregates the most. In a nonpolar solvent such as toluene, **2b** formed uniform self-assemblies, while in more polar solvents, the aggregates were more dispersed in size. Overall, the results indicate that the solvent polarity affects the aggregation of the BODIPYs to various degrees.

**Figure 9:**
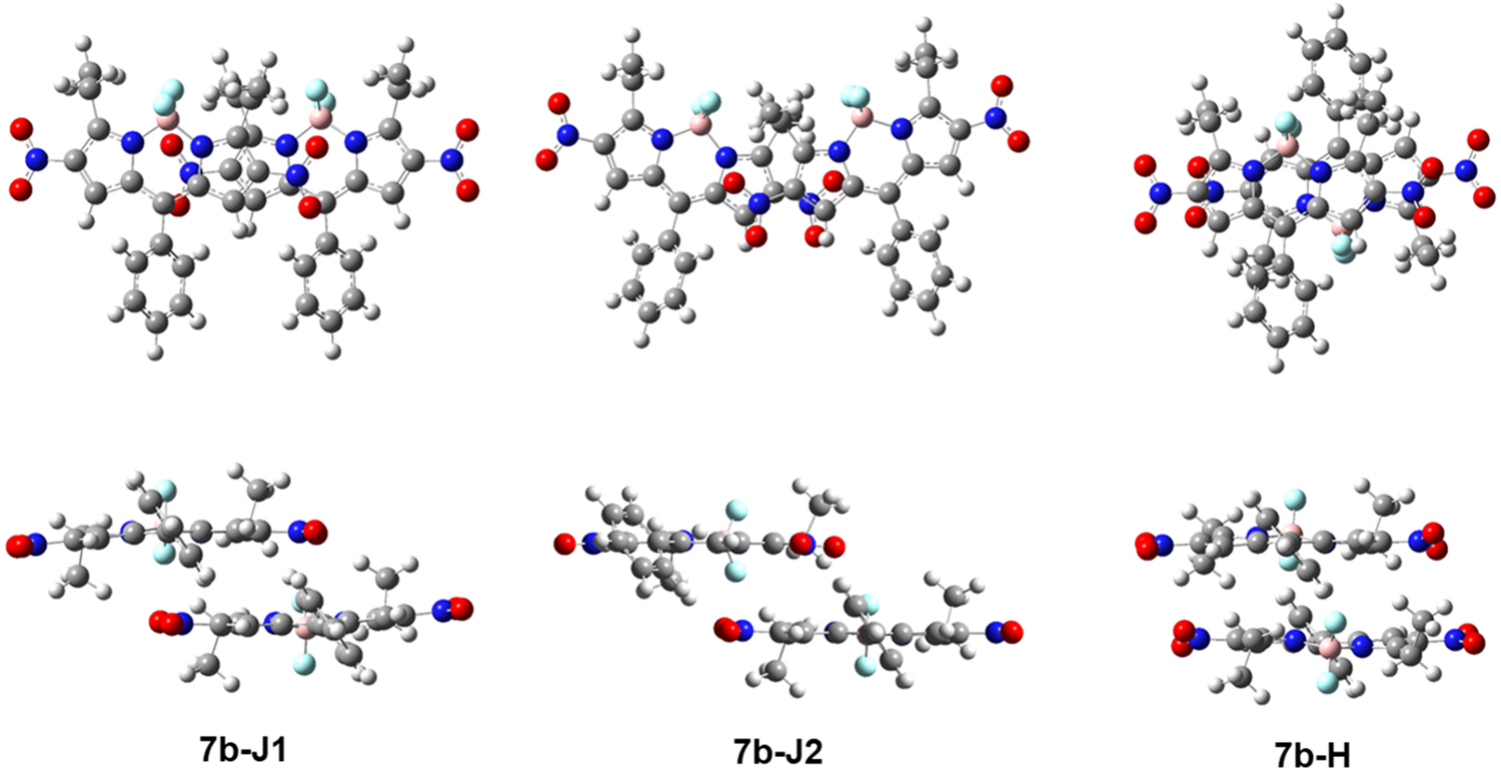
Front view (top) and side view (bottom) of the possible stable dimers of BODIPY **7b** optimized using ωB97X-D/6-31+G(d,p) in acetonitrile.

DFT calculations on potential arrangements of the BODIPY monomers show that the formation of stable J-type and H-type dimers for BODIPY **7b** is possible (Figure 9). Two types of J-type dimers and one type of H-type dimer were identified. The bonding energies for these species are similar and range between 19 and 25 kcal/mol. These calculations suggest different types of self-assembly of monomers, which might explain the different morphologies observed by AFM. Further DFT studies of these complexes are currently underway in our laboratory.

## 3 Conclusions

We report a mild and efficient method for the introduction of one or two nitro groups at the 2-, 3-, or 2,6-positions of BODIPYs in moderate to high yields. The strong electron-withdrawing character of the nitro group strongly influences the spectroscopic properties and polarity of the BODIPYs. The introduction of a nitro group at the 2- or 3-position of BODIPYs induces marked decreases in molar absorptivity, along with hypsochromic shifts in the absorption bands, due to larger HOMO–LUMO gaps induced by the strongly electron-withdrawing nitro group. In addition, the mononitro-BODIPYs show increased Stokes shifts due to larger structural differences between their ground and excited states compared to the symmetric compounds. Introduction of a second nitro group produces symmetrical BODIPYs with red-shifted absorption and emission spectra and decreased polarity compared with the mononitro-BODIPYs. The fluorescence properties and self-assembly of the nitro-substituted BODIPYs are highly dependent on the polarity of the solvent; polar solvents tend to quench the fluorescence emission, while nonpolar solvents significantly increase their fluorescence. Furthermore, the self-assembly properties of select nitro-BODIPYs were shown to depend on the polarity of the solvent using AFM. Our results show that nitro-substituted BODIPYs can find applications in bioimaging and as polarity sensors because their properties are very sensitive to solvent polarity. These compounds are also valuable precursors to other functionalized BODIPYs bearing, for example, amino, amide, and isothiocyanate groups, which can find applications in other areas.

## 4 Experimental Section

### 4.1 General

All reagents and solvents were purchased from commercial vendors and used as received without further purification. Reactions were conducted in oven-dried glassware and monitored by plastic-backed silica thin-layer chromatography plates. Purification of the compounds was performed via silica gel column chromatography (60 Å, 40–63 μm) and preparative TLC. All NMR spectra were recorded using a Bruker spectrometer operating at 400 or 500 MHz for ^1^H, 128 MHz for ^11^B, and 101 or 126 MHz for ^13^C. Chemical shifts (δ) are given in ppm relative to CDCl_3_ (7.26 ppm for ^1^H and 77.16 ppm for ^13^C) or acetone-d_6_ (2.05 ppm for ^1^H and 29.80 ppm for ^13^C). Coupling constants (*J*) are reported in hertz. Peak multiplicity is indicated as follows: s (singlet), d (doublet), t (triplet), q (quartet), dd (doublet of doublets), and m (multiplet). High-resolution mass spectrometry (HRMS) data were obtained at the LSU Mass Spectrometry Facility (MSF) by using an Agilent 6230 ESI-TOF mass spectrometer and MALDI. BODIPYs **1**,^21^**2**,^22^**3**,^23^**5**,^24,35^ and **7**,^27^ were prepared as previously reported, and their spectroscopic data agrees with the literature reports. Caution: the synthetic procedures use flammable organic solvents (acetone, chloroform, DCM, DCE, ethyl acetate, *tert*-butylmethyl ether, and hexanes) as reaction solvents and for purification of the products.

### 4.2 Synthesis and Characterization

#### 1,3,5,7-Tetramethyl-2-nitro-BODIPY (**1a**)

BODIPY **1** (15 mg, 0.060 mmol) was dissolved in dry DCE (10 mL) in an oven-dried 25 mL round-bottomed flask. A solution of nitronium tetrafluoroborate (NO_2_BF_4_) in sulfolane (1 equiv, 0.12 mL) was added dropwise, and the mixture was refluxed for 1 h under a N_2_ atmosphere. The solvent was removed under reduced pressure. The dark brown residue was dissolved in *tert*-butylmethyl ether (10 mL), and the resulting solution was washed with water 3–5 times. The organic portions were collected and dried over Na_2_SO_4_, and the solvent was evaporated under reduced pressure. The crude product was purified by column chromatography using 40% ethyl acetate in hexanes to give a reddish powder in 71% yield. ^1^H NMR (400 MHz, CDCl_3_) δ: 7.24 (s, 2H), 6.29 (s, 1H), 2.86 (s, 3H), 2.63 (s, 3H), 2.56 (s, 3H), 2.34 (s, 3H).

^13^C NMR (126 MHz, CDCl_3_) δ: 166.44, 150.47, 147.03, 137.74, 133.34, 128.01, 123.14, 123.12, 121.79, 15.60, 14.69, 11.76, 11.69. ^11^B NMR (128 MHz, CDCl_3_) δ 0.65 (t, *J* = 31.9 Hz). HRMS (ESI-TOF) *m*/*z* [M-H]^−^calcd for C_13_H_13_BF_2_N_3_O_2_ 292.1069 found 292.1069.

#### 1,3,5,7-Tetramethyl-2,6-dinitro-BODIPY (**1b**)

This compound was prepared as described above for **1a** using **1** (20.1 mg, 0.081 mmol) in dry DCE (15 mL). NO_2_BF_4_ in sulfolane (10 equiv,1.62 mL) was added dropwise, and the final mixture was stirred for 2 h. Column chromatography was performed using 3:2:5 DCM/ethyl acetate/hexanes. The title BODIPY was obtained in 75% yield as a reddish powder. ^1^H NMR (500 MHz, acetone-d_6_) δ: 8.52 (s, 1H), 2.88 (s, 6H), 2.72 (s, 6H). ^13^C NMR (126 MHz, acetone-d_6_) δ 156.92, 142.51, 140.77, 132.14, 130.99,15.18,11.83. ^11^B NMR (128 MHz, CDCl_3_) δ 0.59 (t, *J* = 31.0 Hz). HRMS (ESI-TOF) *m*/*z* [M-H]^−^calcd for C_13_H_12_BF_2_N_4_O_4_ 337.0942 found 337.0942.

#### 1,3,5,7-Tetramethyl-2-nitro-8-phenyl-BODIPY (**2a**)

This compound was prepared as described above for **1a** using **2** (75 mg, 0.231 mmol) in dry DCE (25 mL). NO_2_BF_4_ in sulfolane (1 equiv, 0.46 mL) was added dropwise, and the mixture was stirred at reflux under nitrogen for 3 h. Column purification was done using 3:2:5 DCM/ethyl acetate/hexanes. The title BODIPY **2a** was obtained in 69% yield as a reddish-black powder. ^1^H NMR (400 MHz, CDCl_3_) δ 7.57–7.53 (m, 3H), 7.32–7.26 (m, 2H), 6.21 (s, 1H), 2.86 (s, 3H), 2.64 (s, 3H), 1.65 (s, 3H), 1.43 (s, 3H). ^13^C NMR (100 MHz, CDCl_3_) δ: 164.23, 149.25, 148.77, 143.75, 135.04, 134.89, 133.73, 129.80, 129.59, 127.67, 127.10, 124.92, 124.89,124.84, 15.25, 15.00, 14.25, 12.29. ^11^B NMR (128 MHz, CDCl_3_) δ 0.55 (t, *J* = 31.9 Hz). HRMS (ESI-TOF) *m*/*z* [M-H]^−^calcd for C_19_H_16_BF_2_N_4_O_4_ 413.1233 found 413.1244.

#### 1,3,5,7-Tetramethyl-2,6-dinitro-8-phenyl-BODIPY (**2b**)

This compound was prepared as described above for **1a** using **2** (20 mg, 0.062 mmol) in dry DCE (15 mL). NO_2_BF_4_ in sulfolane (3 equiv, 0.37 mL) was added dropwise, and the reaction was refluxed for 8 h. The title BODIPY **2b** was obtained in 70% yield as a reddish-black powder. ^1^H NMR (400 MHz, CDCl_3_) δ 7.69–7.58 (m, 3H), 7.32–7.28 (m, 2H), 2.91 (s, 6H), 1.72 (s, 6H). ^13^C NMR (101 MHz, CDCl_3_) δ: 155.32, 150.06, 141.95, 141.50, 132.72, 130.81, 130.20, 129.70, 127.23, 14.75, 13.15. ^11^B NMR (128 MHz, CDCl_3_) δ 0.43 (t, *J* = 31.0 Hz). HRMS (ESI-TOF) *m*/*z* [M + H]^+^ calcd for C_19_H_18_BF_2_N_4_O_4_ 415.1396 found 415.1389.

#### 1,3,5,7-Tetramethyl-2,6-dinitro-8-(3-pyridyl)-BODIPY (**3b**)

This compound was prepared as described above for **1a** using **3** (17.5 mg, 0.054 mmol) in dry DCE (10 mL). NO_2_BF_4_ in sulfolane (15 equiv, 1.61 mL) was added, and the reaction was refluxed for 12h. The title BODIPY **3b** was obtained in 71% yield as a reddish powder. ^1^H NMR (400 MHz, CDCl_3_) δ 8.92 (dd, *J* = 4.9, 1.7 Hz, 1H), 8.61 (d, *J* = 2.3 Hz, 1H), 7.69 (dt, *J* = 7.9, 2.0 Hz, 1H), 7.60 (dd, *J* = 7.8, 4.8 Hz, 1H), 2.92 (s, 6H), 1.73 (s, 6H). ^13^C NMR (126 MHz, CDCl_3_). ^13^C NMR (126 MHz, CDCl_3_) δ: 156.26, 152.01, 147.65, 141.57, 135.51, 124.57, 14.97, 13.89. ^11^B NMR (128 MHz, CDCl_3_) δ 0.40 (t, *J* = 30.8 Hz). HRMS (ESI-TOF) *m*/*z* [M + H]^+^calcd for C_18_H_17_BF_2_N_5_O_4_ 416.1344 found 416.1342.

#### 1,3,5,7-Tetramethyl-8-(2-methoxy-3-pyridyl)-BODIPY (**4**)

BODIPY **4** was synthesized by condensing 6-methoxypyridine-3-carbaldehyde with 2,4-dimethylpyrrole in the presence of trifluoroacetic acid (TFA) under nitrogen for 15 h. The resulting intermediate was oxidized with 2,3-dichloro-5,6-dicyano-1,4-benzoquinone (DDQ) for 2 h, followed by boron complexion of the dipyrromethene intermediate using boron trifluoride diethyl etherate (BF_3_.OEt_2_) in triethylamine for 20 h. The reaction was then stopped, and the excess solvent was evaporated under reduced pressure. The crude residue was dissolved in DCM and washed with 100 mL of water two times. The organic layer was extracted by using DCM and dried over Na_2_SO_4_. The solvent was removed under reduced pressure, and column chromatography was carried out using 20% ethyl acetate in hexanes to obtain the desired compound in 35% yield. ^1^H NMR (400 MHz, CDCl_3_) δ 8.05 (d, *J* = 2.4 Hz, 1H), 7.44 (dd, *J* = 8.4, 2.5 Hz, 1H), 6.87 (d, *J* = 8.4 Hz, 1H), 5.99 (s, 2H), 3.99 (s, 3H), 2.54 (s, 6H), 1.47 (s, 6H). ^13^C NMR (101 MHz, CDCl_3_) δ: 164.57, 156.06, 145.78, 142.94, 138.74, 138.14, 132.03, 123.96, 121.66, 111.43, 53.75, 15.23, 14.65. ^11^B NMR (128 MHz, CDCl_3_) δ 0.73 (t, *J* = 33.0 Hz). HRMS (ESI-TOF) *m*/*z* [M + H]^+^calcd for C_19_H_21_BF_2_N_3_O 356.1754 was found 356.1746.

#### 1,3,5,7-Tetramethyl-8-(2-methoxy-3-pyridyl)-2-nitro-BODIPY (**4a**)

This compound was prepared as described above for **1a** using **4** (16 mg, 0.045 mmol) in dry DCM (10 mL). NO_2_BF_4_ in sulfolane (1.1 equiv, 0.1 mL) was added dropwise, and the mixture was stirred at room temperature under nitrogen for 2 h. Column chromatography was done using 20% ethyl acetate in hexanes. The title compound **4a** was obtained in 90% yield as a reddish powder. ^1^H NMR (500 MHz, CDCl_3_) δ 8.07 (d, *J* = 2.5 Hz, 1H), 7.47 (dd, *J* = 8.5, 2.5 Hz, 1H), 6.95 (d, *J* = 8.4 Hz, 1H), 6.24 (s, 1H), 4.03 (s, 3H), 2.86 (s, 3H), 2.64 (s, 3H), 1.78 (s, 3H), 1.54 (s, 3H). ^13^C NMR (101 MHz, CDCl_3_) 165.11, 165.04, 149.74, 148.56, 145.70, 140.47, 139.34, 138.41, 135.88, 134.74, 127.63, 125.46, 122.84, 112.09, 54.05, 16.10, 15.52, 14.47, 13.24. ^11^B NMR (128 MHz, CDCl_3_) δ 0.52 (t, *J* = 31.7 Hz). HRMS (ESI-TOF) *m*/*z* [M + H] ^+^ calcd for C_19_H_20_N_4_O_3_BF_2_: 401.1610 found 401.1597.

#### 1,3,5,7-Tetramethyl-8-(2-methoxy-3-pyridyl)-2,6-dinitro-BODIPY(**4b**)

This compound was prepared as described above for **1a** using **4** (16.5 mg, 0.046 mmol) in dry DCE (10 mL). NO_2_BF_4_ in sulfolane (10 equiv, 0.93 mL) was added, and the reaction refluxed for 3.5 h. column chromatography was carried out using 20% ethyl acetate in hexanes to obtain BODIPY **4b** in 96% yield as a reddish powder. ^1^H NMR (400 MHz, CDCl_3_) δ 8.09 (d, *J* = 2.5 Hz, 1H), 7.48 (dd, *J* = 8.5, 2.5 Hz, 1H), 7.01 (d, *J* = 8.5 Hz, 1H), 4.05 (s, 3H), 2.91 (s, 6H), 1.85 (s, 6H). ^13^C NMR (126 MHz, CDCl_3_) δ: 165.69, 155.86, 146.97, 145.47, 141.77, 141.69, 137.96, 130.37, 121.85, 112.68, 54.28, 14.93, 14.17. ^11^B NMR (128 MHz, CDCl_3_) δ 0.44 (t, *J* = 30.9 Hz). HRMS (ESI-TOF) *m*/*z* [M + H] ^+^ calcd for C_19_H_19_BN_5_O_5_F_2_ 446.1454 found 446.1447.

#### 2-Nitro-8-phenyl-BODIPY (**5a**) and 3-Nitro-8-phenyl-BODIPY (**6**)

Compounds **5a** and **6** were prepared as described above for **1a** using **5** (15 mg, 0.056 mmol) in dry DCE (10 mL). NO_2_BF_4_ in sulfolane (2 equiv, 0.22 mL) was added dropwise, and the mixture was refluxed for 7 h. Preparative TLC was done using 25% ethyl acetate in hexanes to obtain compounds **5a** and **6** in 7 and 83% yields, respectively. Data for **5a**: ^1^H NMR (400 MHz, CDCl_3_) δ 8.36 (s, 1H), 8.27 (s, 1H), 7.73–7.66 (m, 1H), 7.70–7.61 (m, 1H), 7.65–7.55 (m, 6H), 7.32 (s, 1H), 7.23 (d, *J* = 4.6 Hz, 2H), 6.79 (d, *J* = 4.7 Hz, 1H), 1.54 (s, 1H). ^13^C NMR (126 MHz, CDCl_3_) δ: 151.87, 149.77, 141.93, 137.90, 136.35, 135.58, 132.51, 132.15, 132.02, 130.64, 129.19, 122.67, 122.17. ^11^B NMR (128 MHz, CDCl_3_) δ −0.01 (t, *J* = 27.4 Hz). HRMS (ESI-TOF) *m*/*z* [M + H] ^+^calcd for C_15_H_11_BF_2_N_3_O_2_ 314.0912 found 314.0908. Data for **6**: 8.33 (s, 1H), 7.69–7.65 (m, 1H), 7.63–7.56 (m, 3H), 7.56–7.51 (m, 1H), 7.18 (dd, *J* = 8.0, 4.5 Hz, 2H), 6.82 (dd, *J* = 4.7, 1.6 Hz, 1H), 6.76 (d, *J* = 4.4 Hz, 1H). ^13^C NMR (101 MHz, CDCl_3_) δ: 153.77, 150.78, 149.23, 138.05, 136.35, 134.48, 132.68, 131.82, 130.76, 128.98, 126.72, 123.91, 115.01. ^11^B NMR (128 MHz, CDCl_3_) δ 0.34 (t, *J* = 24.9 Hz). HRMS (ESI-TOF) *m*/*z* [M + H]^+^ and [M + Na]^+^ calcd for C_15_H_11_BF_2_N_3_O_2_, C_15_H_10_BF_2_N_3_O_2_Na 314.0912, 336.0732 found 314.0917 and 336.0742, respectively.

#### 3,5-Diethyl-2-nitro-8-phenyl-BODIPY (**7a**)

This compound was prepared as described above for **1a** using BODIPY **7** (20 mg, 0.062 mmol) in dry DCE (15 mL). NO_2_BF_4_ in sulfolane (2 equiv, 0.25 mL) was added, and the reaction was refluxed for 3 h. The title BODIPY **7a** was obtained in 87% yield as an orange-reddish powder. ^1^H NMR (400 MHz, CDCl_3_) δ 7.61–7.58 (m, 1H), 7.58–7.46 (m, 4H), 7.22 (d, *J* = 1.6 Hz, 1H), 7.05 (d, *J* = 4.6 Hz, 1H), 6.63 (dd, *J* = 4.6, 1.1 Hz, 1H), 3.36 (q, *J* = 7.4 Hz, 2H), 3.16 (q, *J* = 7.6 Hz, 2H), 1.40 (d, *J* = 15.0 Hz, 6H). ^13^C NMR (126 MHz, CDCl_3_) δ: 172.57, 155.08, 144.58, 138.61, 137.38, 135.23, 132.74, 130.98, 130.47, 130.28, 129.30, 129.24, 128.72, 122.45, 122.31, 22.79, 21.45, 13.07, 12.31. ^11^B NMR (128 MHz, CDCl_3_) δ 0.73 (t, *J* = 31.7 Hz). HRMS (ESI-TOF) *m*/*z* [M + H] ^+^calcd for C_19_H_19_BF_2_N_3_O_2_ 370.1538 found 370.1542.

#### 3,5-Diethyl-2,6-dinitro-8-phenyl-BODIPY (**7b**)

This compound was prepared as described above for **1a** using **7** (20.8 mg, 0.064 mmol) in dry DCE (10 mL). NO_2_BF_4_ in sulfolane (5 equiv, 0.64 mL) was added, and the reaction was refluxed for 5 h. The title BODIPY was obtained in 55% yield as an orange-reddish powder. ^1^H NMR (500 MHz, CDCl_3_) δ 7.77–7.69 (m, 1H), 7.64 (dd, *J* = 8.5, 7.0 Hz, 2H), 7.60–7.52 (m, 4H), 3.42 (q, *J* = 7.5 Hz, 4H), 1.43 (t, *J* = 7.4 Hz, 6H). ^13^C NMR (126 MHz, CDCl_3_) δ): 161.60, 151.79, 141.63, 132.63, 131.79, 130.98, 130.61, 129.43, 129.01, 22.15, 13.08. ^11^B NMR (128 MHz, CDCl_3_) δ 0.59 (t, *J* = 30.9 Hz). HRMS (ESI-TOF) *m*/*z* [M + H] ^+^calcd for C_19_H_18_BF_2_N_4_O_4_ 415.1389 found 415.1395.

### 4.3 X-ray Analysis

Data for **2a**, **2b** (two polymorphs), **5a**, **6**, and **7b** were collected on a Bruker Kappa ApexII DUO diffractometer, while those for **1b**, **3b**, **4a**, and **4b** were collected on a Bruker D8 Venture DUO Photon III diffractometer. BODIPY **2b** (both polymorphs), **5a**, and **6** used Mo Kα radiation, while **1b**, **2a**, **4b**, and **7b** used Cu Kα and **3b** and **4a** used Ag Kα. All data were collected at 100 K. Data for the ten structures reported herein have been deposited at the Cambridge Crystallographic Data Centre under deposition numbers CCDC 2345035–2345041, 2357072–2357073, and 2380719.

### 4.4 Spectroscopic Analysis

UV–visible absorption was collected on a Varian Cary 50 spectrophotometer, and emission spectra were recorded on a PerkinElmer LS55 spectrophotometer. All solutions (1 × 10^–6^ M to 5 × 10^–6^ M) were prepared using spectrophotometric-grade solvents (acetonitrile/toluene). Quartz cuvettes (10 mm path length) were used to minimize reabsorption effects. Absolute fluorescence quantum yields of all the samples were collected on an FS5 spectrofluorometer from Edinburgh instruments, using 1 × 10^–6^ M solutions.

### 4.5 Atomic Force Microscopy

Dropcast samples were prepared for AFM studies by depositing a drop (20 μL) of sample onto freshly cleaved mica substrates (ruby muscovite mica, S&J Trading Co. NY). The concentration of BODIPYs **1b**, **2b**, and **7b** that was deposited was 0.10 mM. For the samples dissolved in 80% water in acetonitrile, an additional 20 μL was deposited onto the substrate after the first drop was dried overnight. After the samples were dried in air, AFM images were acquired ex situ using the tapping mode in ambient air. Surface characterizations were obtained with a model 5500 scanning probe microscope (Keysight Technologies, Santa Rosa, CA) equipped with PicoView software (version 1.20.2). Ultrasharp NCHR silicon tips with an aluminum reflex coating were used for tapping-mode AFM at a scan rate of 1.5 line/s, with an average spring constant ranging from 42 N/m and resonant frequencies in the range of 300–350 kHz (Nanoworld, Neuchâtel, Switzerland). Digital images were processed with Gwyddion software, which is freely available from the Czech Metrology Institute.^39^

### 4.6 Theoretical Calculations

All ground and excited states were studied at the ωB97X-D/6-31+G(d,p)^40^ and MN15/6-31+G(d,p)^41^ levels of theory. The solvent effects were taken into account by using the polarized continuum model (PCM). The UV–vis data were calculated using TD-DFT at the MN15/6-31+G(d,p) level of theory. The first three singlet excitations were considered, and the lowest-energy excited singlet state was optimized to calculate the properties reported in this study. Using the MN15 functional was recommended in a recent benchmark study of the photophysical properties of difluoroborane and hydroxyphenylimidazol dyes.^42^ To confirm its applicability for our series of dyes, we tested three additional functionals recommended in the literature for studies of BODIPYs: ωB97X-D, as recommended by Harvey et al.;^43^ TPSSh, as suggested by Lopez and Cardenas-Jiron et al.;^44^ and M06-2X, as recommended by Jacquemin and Le Guennic.^45^ The comparison between the experimental and calculated data is shown in Figure S4 of the Supporting Information. As can be seen in the figure, while the TPSSh method gives vertical excitation energies that are closest to the experimental values, the MN15 method shows a better prediction of the tendencies of the bathochromic and hypsochromic shifts for the molecules in our series. Both methods overestimate the Stokes shifts in a similar way. Therefore, we used the MN15 method to examine the photophysical properties of the entire series of BODIPYs reported in this study.

All calculations were performed using the Gaussian 16 program package.^46^

## Supporting Information Available

NMR data for all BODIPYs, absorption and emission spectra, X-ray data (CIF) (ZIP)
Molecular electrostatic potentials (MESPs), molecular orbitals, and dipole moment orientations (PDF)

## Abbreviations

TD-DFT: time-dependent density-functional theory
NMR: nuclear magnetic resonance
TLC: thin-layer chromatography
HRMS: high-resolution mass spectrometry
HOMO: highest occupied molecular orbital
LUMO: lowest unoccupied molecular orbital
MBTE: *tert*-butylmethyl ether
TFA: trifluoroacetic acid
DDQ: 2,3-dichloro-5,6-dicyano-1,4-benzoquinone
AFM: atomic force microscopy
