# Supplementary material for: Effects of Nitro-Substitution on the Spectroscopic and Self-Assembly Properties of BODIPY Dyes
Source: ACS Omega. 2025 Apr 7;10(15):14723–37. doi: 10.1021/acsomega.4c08799 (PMC12019473; doi:10.1021/acsomega.4c08799)
Supplement: Supplementary file 2 — ao4c08799_si_002.pdf [file ao4c08799_si_002.pdf]

Supporting Information

Effects of Nitro-substitution on the Spectroscopic and Self-Assembly Properties of BODIPY Dyes

Caroline Gwaro,[1,ς] Caroline Ndung'U,[1,ς] Petia Bobadova-Parvanova,[2] Dylan Goliber,[2] Quynh Do,[1] Ashley R. Walker,[1] Evan Murders,[1] Daniel LaMaster,[3] Frank R. Fronczek,[1] Jayne Garno,[1] and Maria da Graça H. Vicente[1,]*

[1]Department of Chemistry, Louisiana State University, Baton Rouge, 70803 LA

[2]Department of Chemistry and Fermentation Sciences, Appalachian State University, Boone, NC 28608

[3]Department of Chemistry, Talladega College, Talladega, AL 35160

[ς]C.G. and C.N. contributed equally.

Table of Contents

Molecular Electrostatic potentials..……………………3

Dipole moments..………...3

Frontier Orbitals...4

Calculated versus experimental maximum absorption wavelengths………………………...5

BODIPY core bond lengths ....……………………6

Absorption and emission spectra of BODIPYs in acetonitrile 7-8

Absorption and emission spectra of BODIPYs in toluene 9-10

Absorption and emission spectra of BODIPYs in 80% water and 20% acetonitrile......... …11

Table of spectroscopic properties in 80% water and 20% acetonitrile..................................11

Atomic force microscopy studies in 80% water and 20% acetonitrile............................... 12-14

^{1}H, ^{13}C and ^{11}B NMR spectra for all BODIPYs... 15-32

Computational data

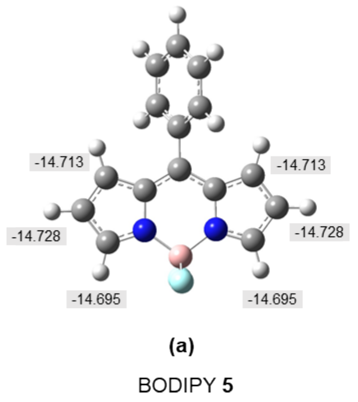

Fig. S1. ωB97X-D/6-31+G(d,p) Molecular Electrostatic Potentials (MESP, a.u.) at the carbon nuclei and MESP maps for a) BODIPY **5**, and b) BODIPY **5cat**.

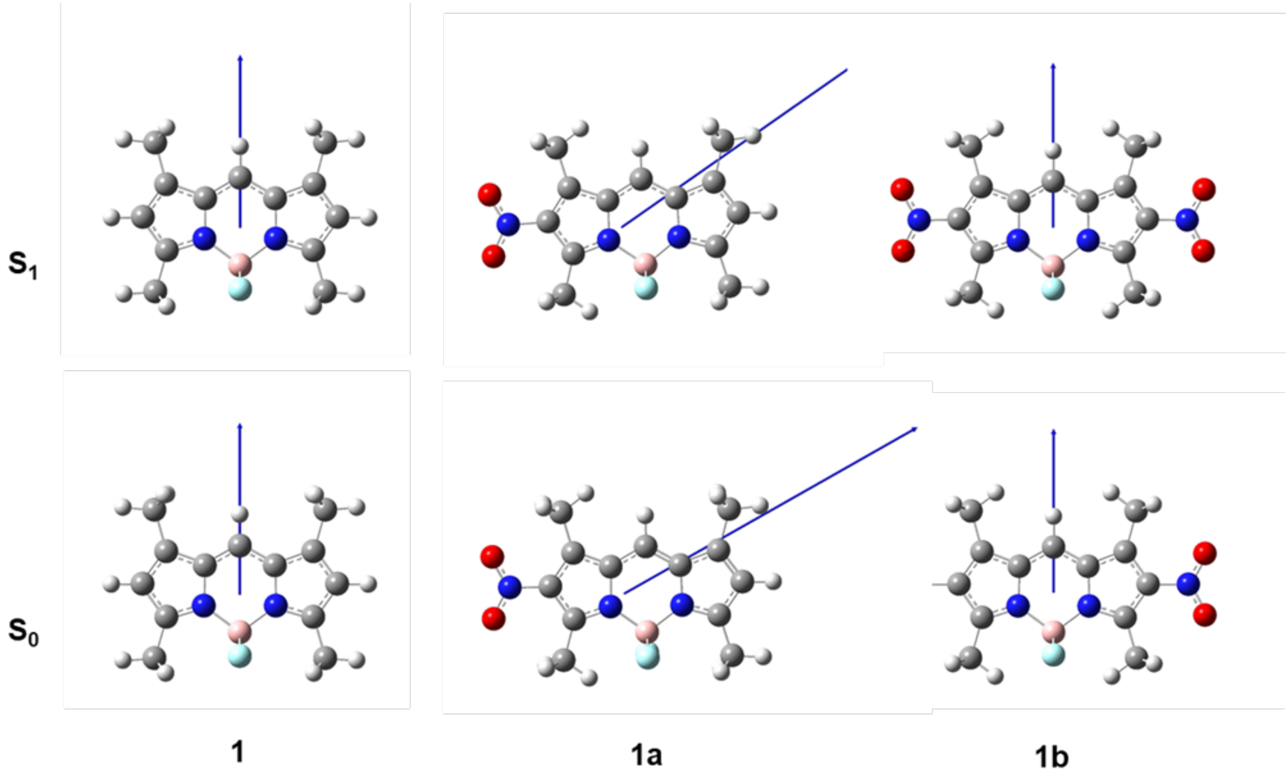

Fig. S2. Dipole moments of the ground (S_0) and excited (S_1) states of unsubstituted **1**, 2-mononitro **1a**, and 2,6-dinitro **1b**. Calculated at the MN15/6-31+G(d,p) level in acetonitrile.

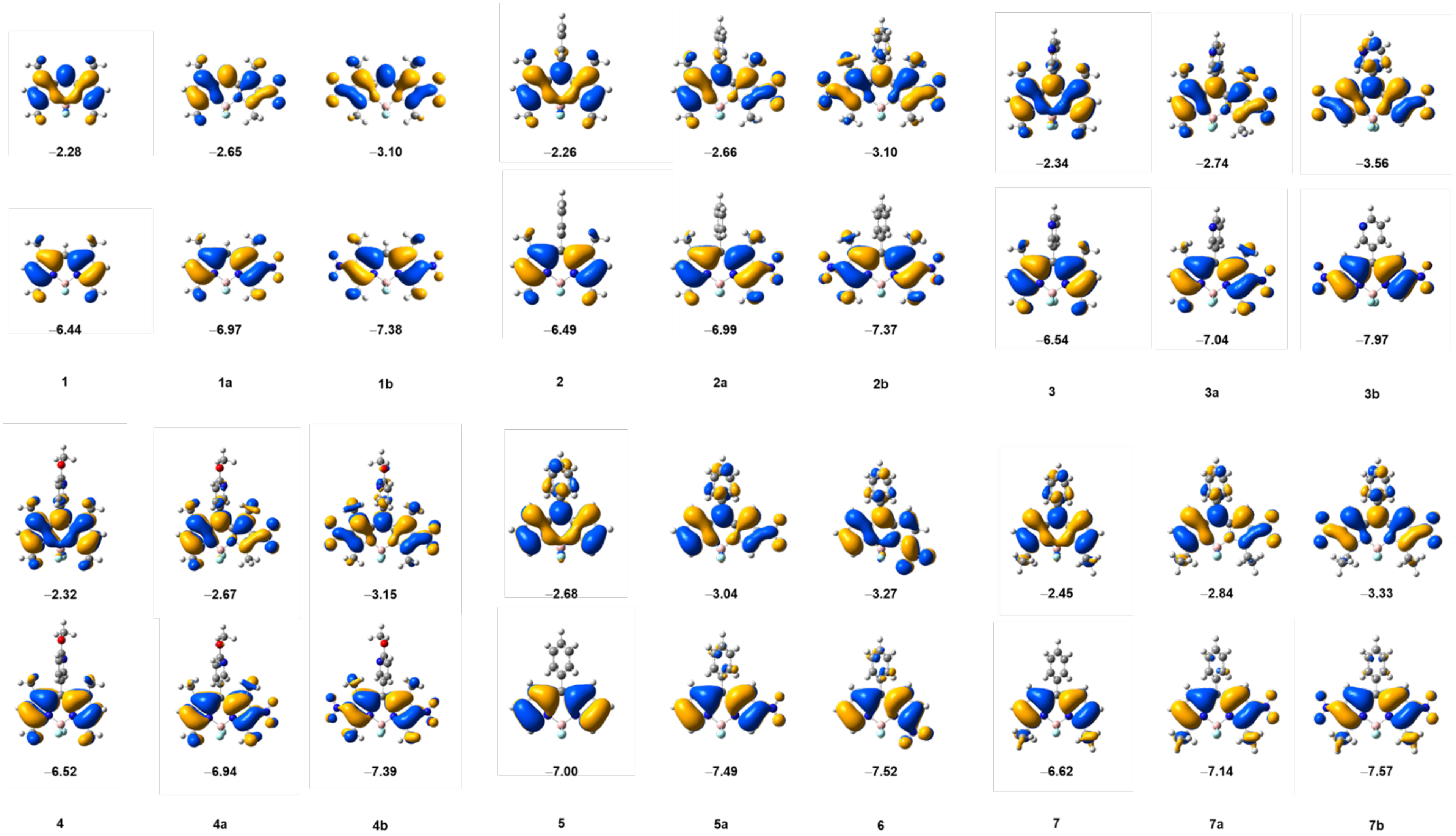

Fig. S3. Frontier orbitals for the series of the BODIPYs studied. Orbital energies in eV

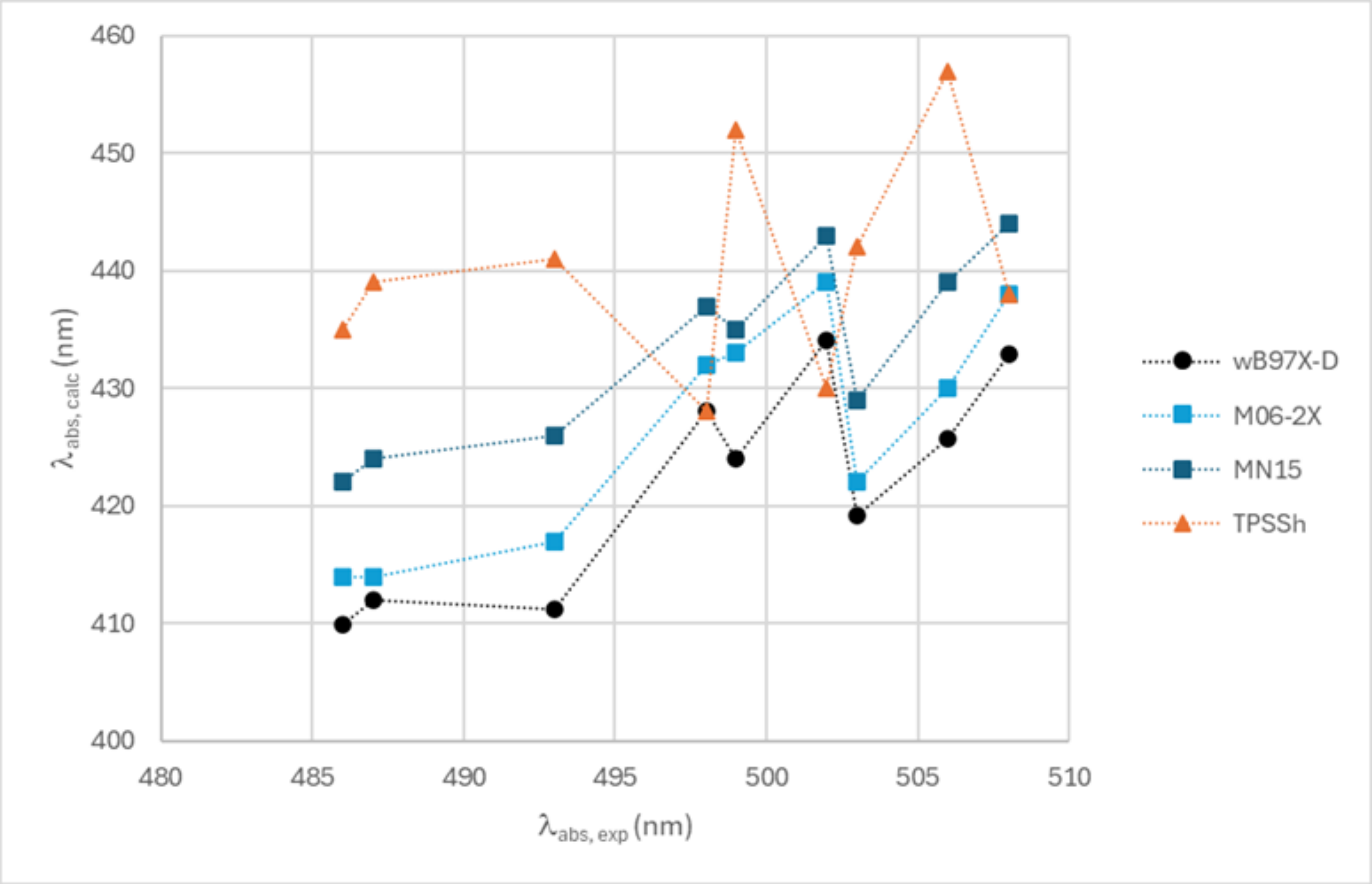

Fig. S4. Calculated ($\lambda_{abs,\ calc}$) versus experimental ($\lambda_{abs,\ exp}$) maximum absorption wavelengths for compounds **1**, **1a**, **1b**, **2**, **2a**, **2b**, **7**, **7a**, and **7b** using different DFT functionals: ωB97X-D, M06-2X, MN15, and TPSSh. All calculations were performed using the 6-31+G(d,p) basis set in acetonitrile.

Table S1. MN15/6-31+G(d,p) calculated BODIPY core bond lengths for the ground and excited states of **1, 1a, 1b, 7, 7a, 7b.** All parameters are calculated in acetonitrile.

Compound	S_0			S_1			Change		
	R_{B-N} (Å)	R_{N-C} (Å)	$R_{C-C_{meso}}$ (Å)	R_{B-N} (Å)	R_{N-C} (Å)	$R_{C-C_{meso}}$ (Å)	ΔR_{B-N} (Å)	ΔR_{N-C} (Å)	$\Delta R_{C-C_{meso}}$ (Å)
**1**	1.549	1.350	1.391	1.542	1.361	1.406	–0.007	+0.011	+0.015
**1a**									
nitro side	1.562	1.342	1.410	1.567	1.340	1.400	+0.005	–0.003	–0.010
non-nitro side	1.550	1.342	1.372	1.537	1.366	1.409	–0.014	+0.024	+0.036
**1b**	1.562	1.337	1.391	1.559	1.343	1.405	–0.003	+0.006	+0.014
**2**	1.544	1.349	1.402	1.537	1.359	1.417	–0.007	+0.010	+0.016
**2a**									
nitro side	1.554	1.341	1.425	1.558	1.339	1.414	+0.004	–0.002	–0.011
non-nitro side	1.544	1.341	1.385	1.533	1.363	1.424	–0.011	+0.022	+0.039
**2b**	1.554	1.337	1.406	1.552	1.343	1.420	–0.002	+0.006	+0.013
**7**	1.549	1.351	1.404	1.542	1.358	1.420	–0.008	+0.007	+0.017
**7a**									
nitro side	1.557	1.348	1.426	1.562	1.342	1.411	+0.004	–0.006	–0.015
non-nitro side	1.553	1.341	1.384	1.539	1.360	1.425	–0.014	+0.019	+0.041
**7b**	1.561	1.342	1.407	1.558	1.345	1.418	–0.003	+0.002	+0.011

Absorbance(right) and Emission (left) spectra.

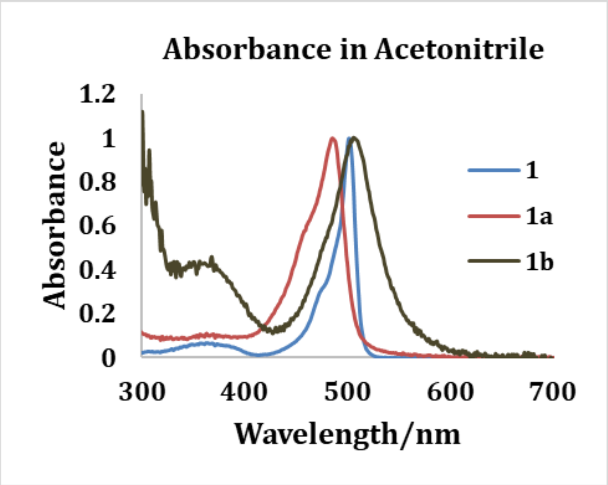

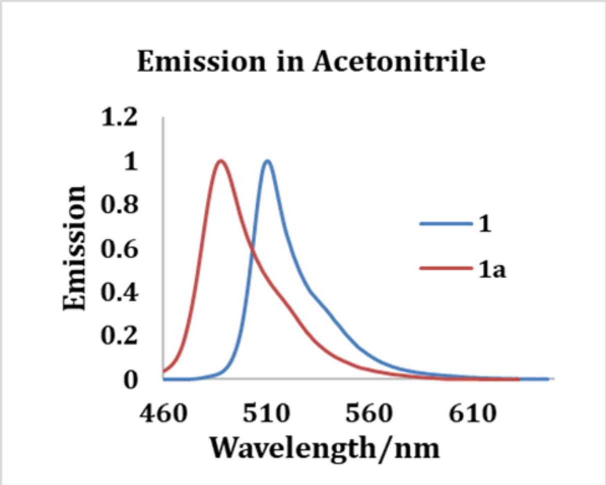

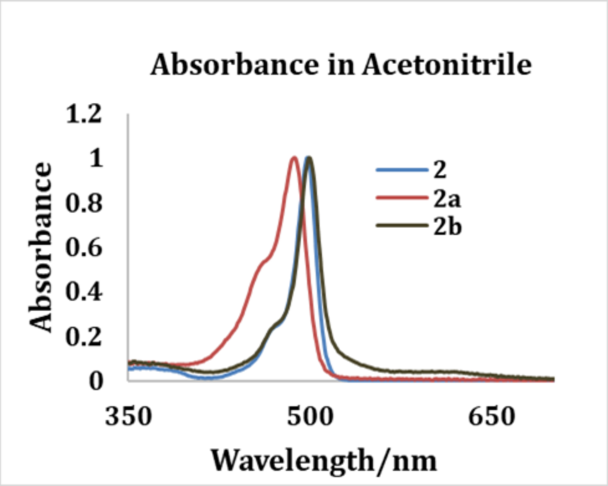

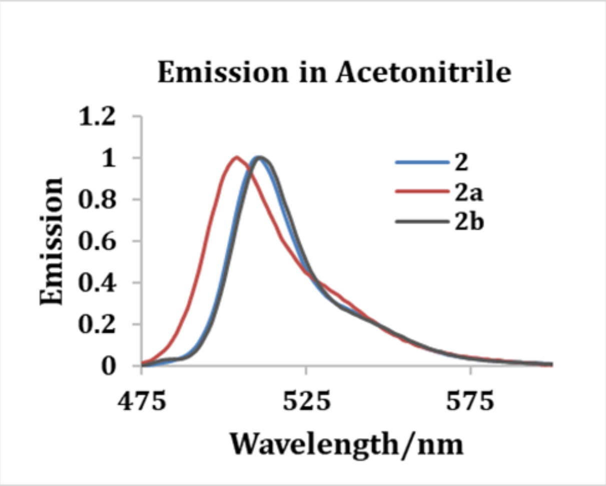

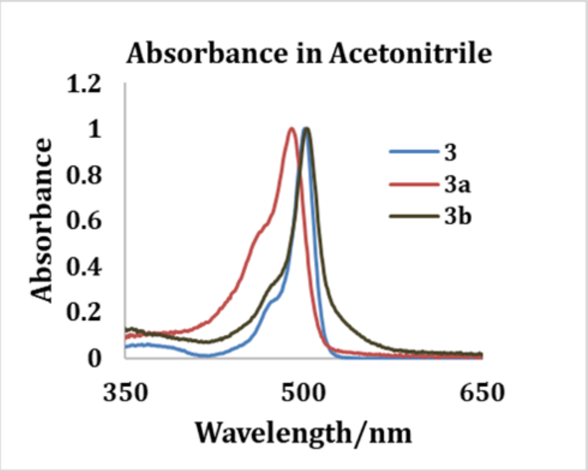

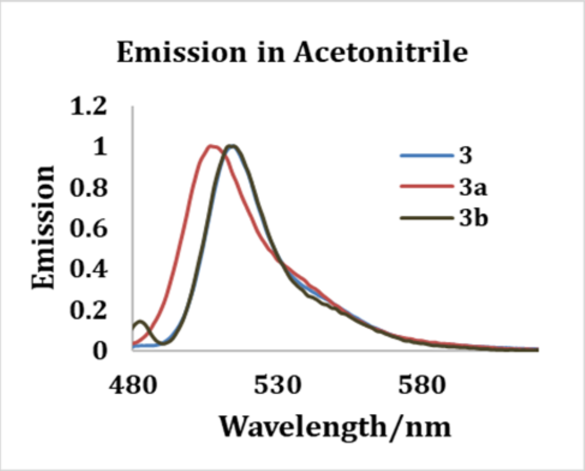

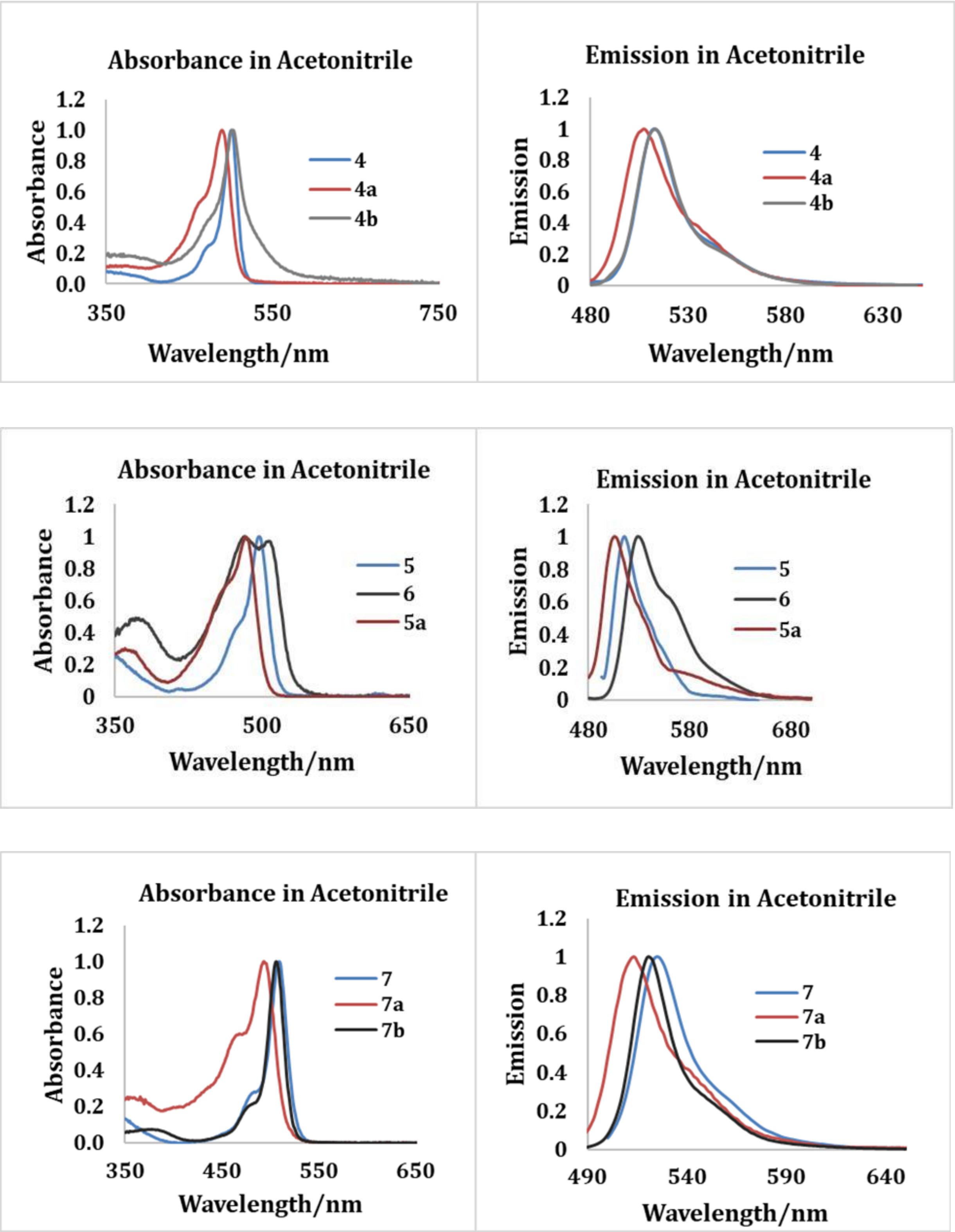

Fig. S5: Normalized absorption (left) and emission (right) spectra of BODIPYs in acetonitrile.

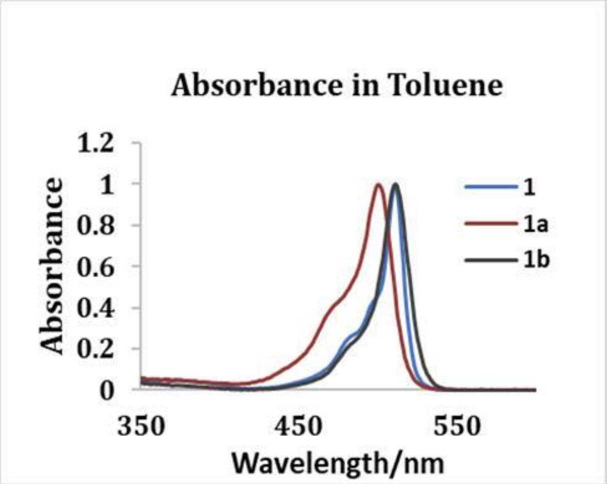
Absorbance in Toluene
Absorbance
Wavelength/nm
1.2
1
0.8
0.6
0.4
0.2
0
350
450
550
1
1a
1b

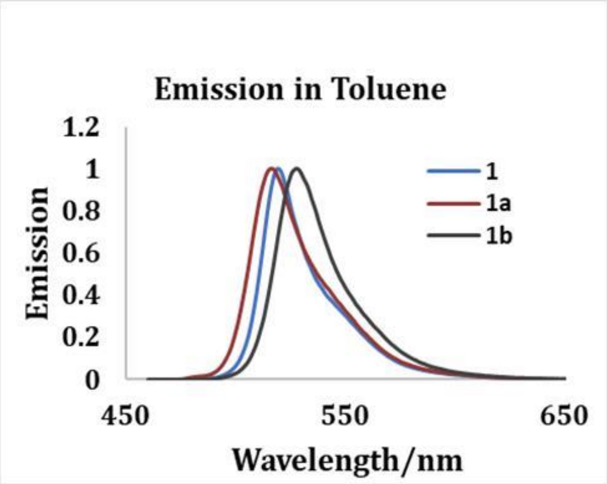
Emission in Toluene
Emission
Wavelength/nm
1.2
1
0.8
0.6
0.4
0.2
0
450
550
650
1
1a
1b

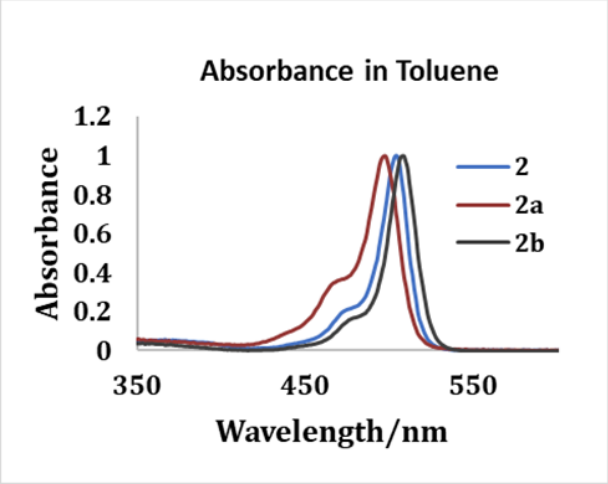
Absorbance in Toluene
Absorbance
Wavelength/nm
1.2
1
0.8
0.6
0.4
0.2
0
350
450
550
2
2a
2b

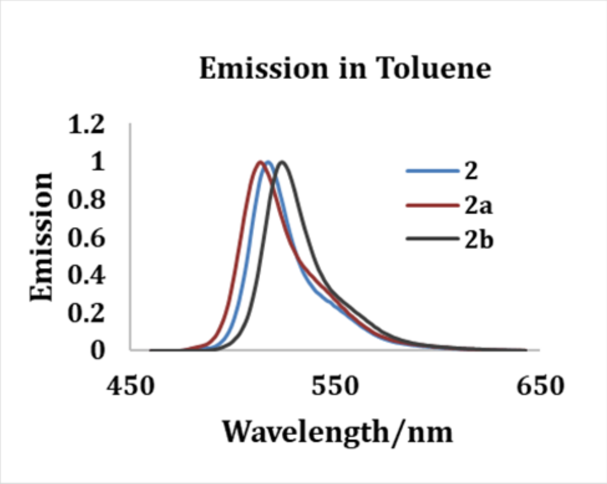
Emission in Toluene
Emission
Wavelength/nm
1.2
1
0.8
0.6
0.4
0.2
0
450
550
650
2
2a
2b

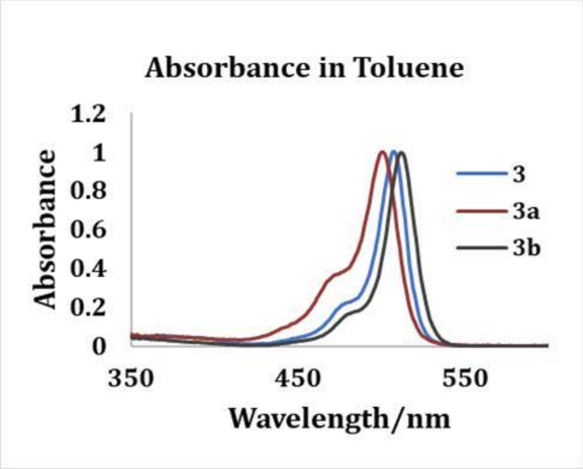
Absorbance in Toluene
Absorbance
Wavelength/nm
1.2
1
0.8
0.6
0.4
0.2
0
350
450
550
3
3a
3b

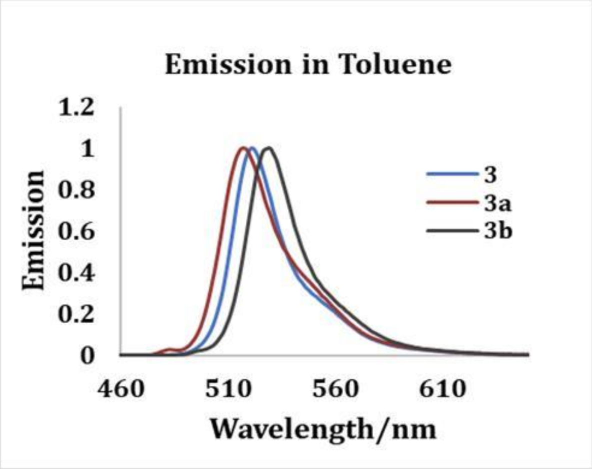
Emission in Toluene
Emission
Wavelength/nm
1.2
1
0.8
0.6
0.4
0.2
0
460
510
560
610
3
3a
3b

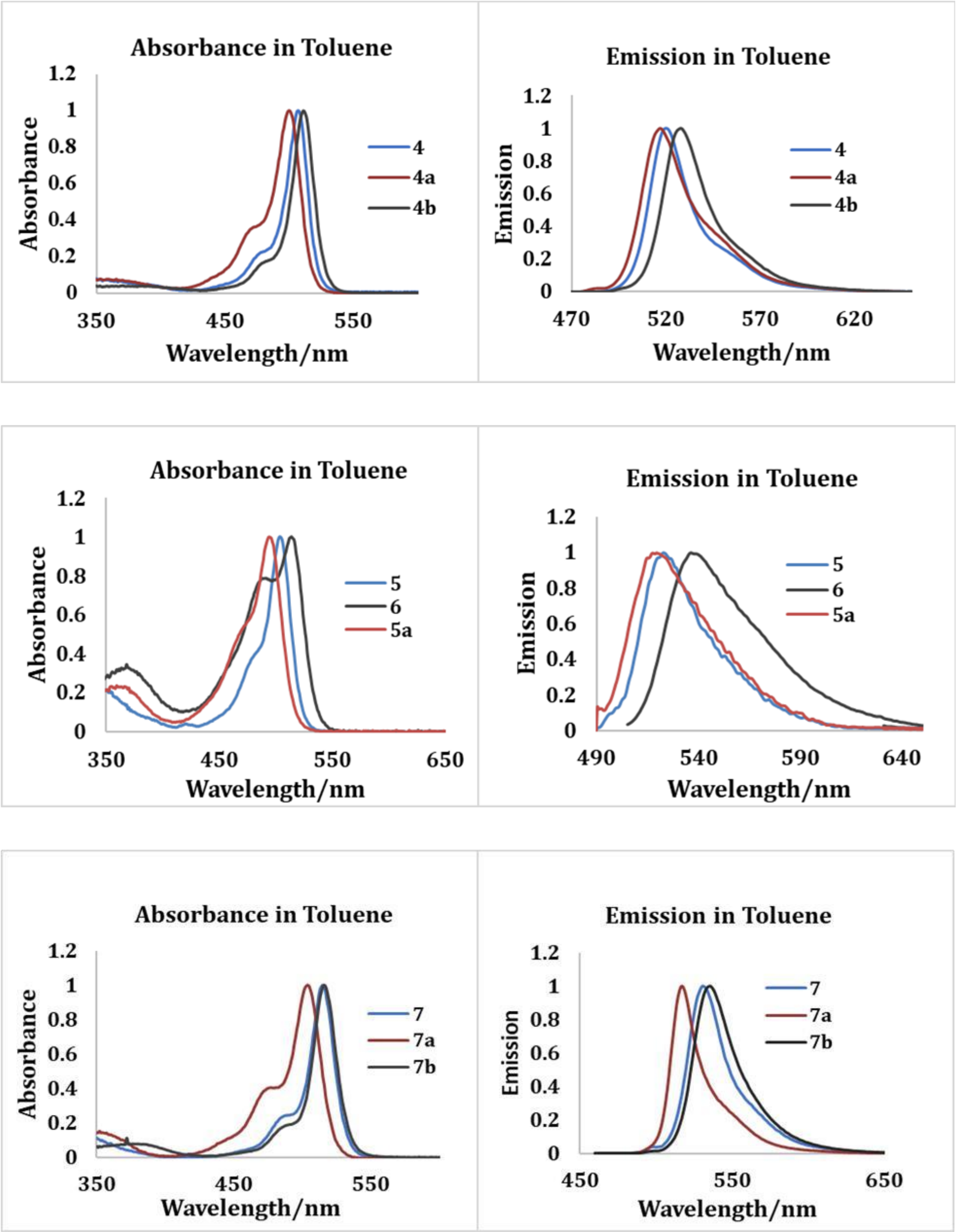

Fig. S6: Normalized absorption (left) and emission (right) spectra of BODIPYs in toluene.

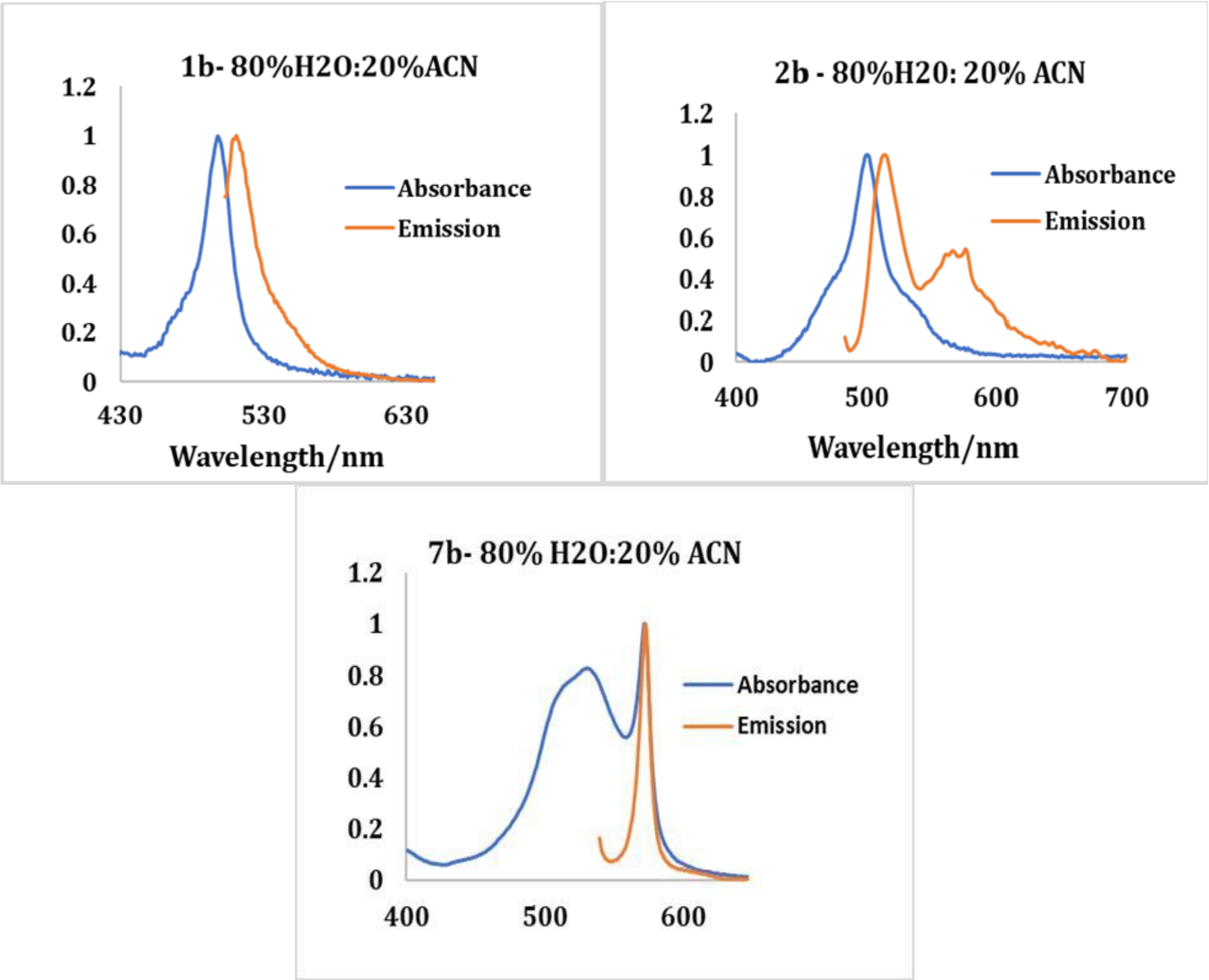

Fig. S7: Normalized absorption and Emission spectra of BODIPYs **1b,2b** and **7b** 80% water and 20% acetonitrile (ACN)

Table S2: Spectroscopic studies of BODIPYs **1b,2b**, and **7b** in 80% water and 20% acetonitrile

BODIPY	80% Water:20% Acetonitrile		
	λ_{abs}^{max}/nm	λ_{em}^{max} /nm (Φ_f)	Stokes shift/nm
**1b**	498	511(0.00)	13
**2b**	500	514(0.71)	14
**7b**	572	573 (1.76)	1

Atomic force microscopy

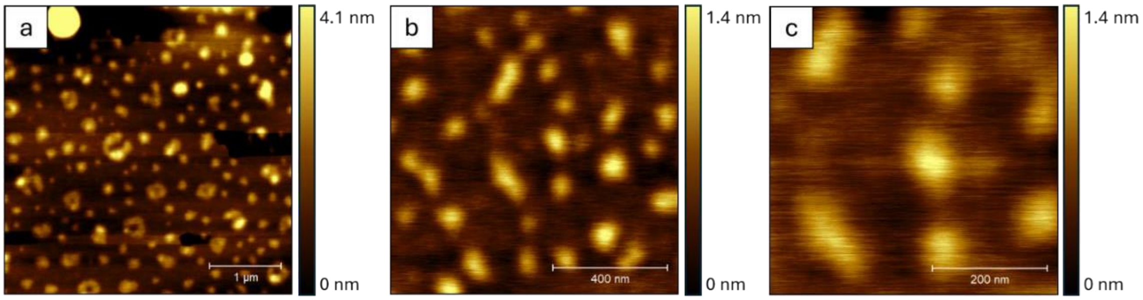

Fig. S8. Topography images of BODIPY **1b** in 80% water and 20% acetonitrile on mica. (a) A 4×4 µm² and subsequent (b) 1×1 µm² and (c) 500×500 nm² zoom-in views of a region.

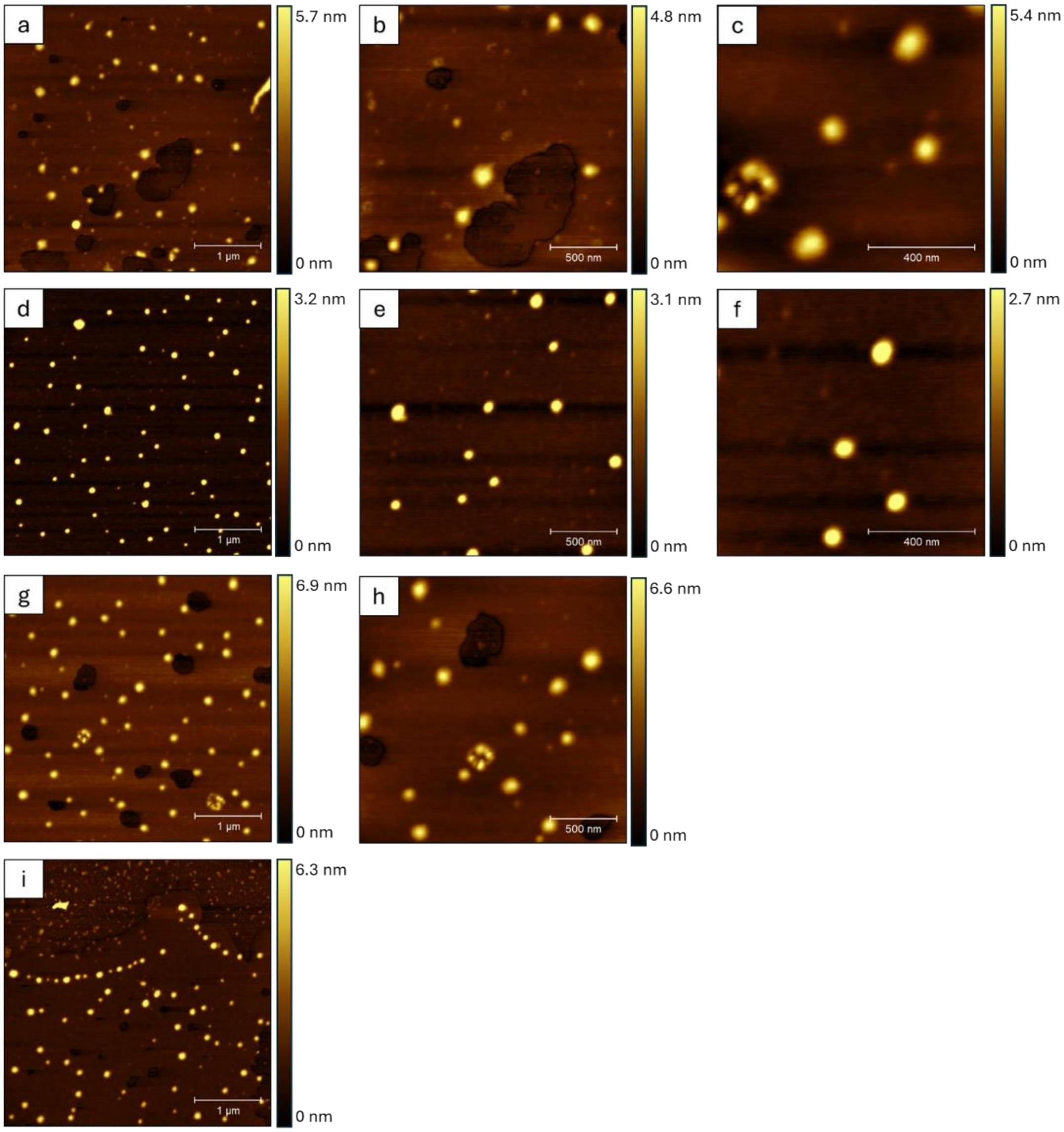

Fig. S9. AFM topography frames (a-i) of BODIPY **2b** in 80% water and 20% acetonitrile deposited on mica. Each row denotes a different area on the surface. (a, d, g, and i); 4×4 µm². (b, e and h); 2×2 µm². (c and f); 1×1 µm² close-up views.

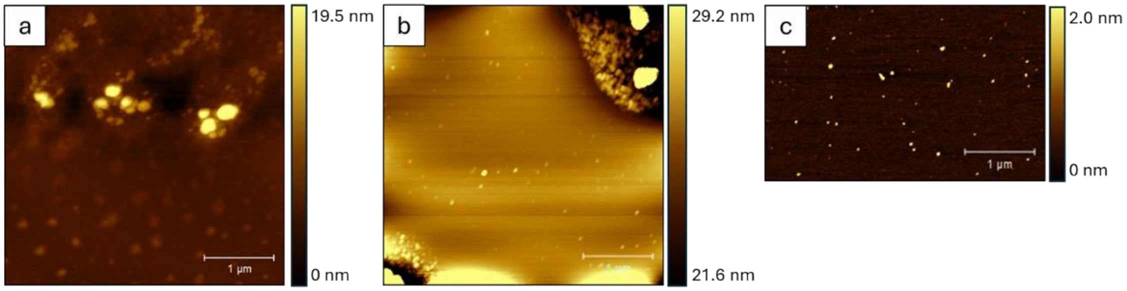

Fig. S10. AFM topography images of BODIPY **7b** aggregates in 80% water and 20% acetonitrile on mica.(a) 4×4 µm²; (b) 4×4 µm²; and (c) 3×4 µm² images of different regions.

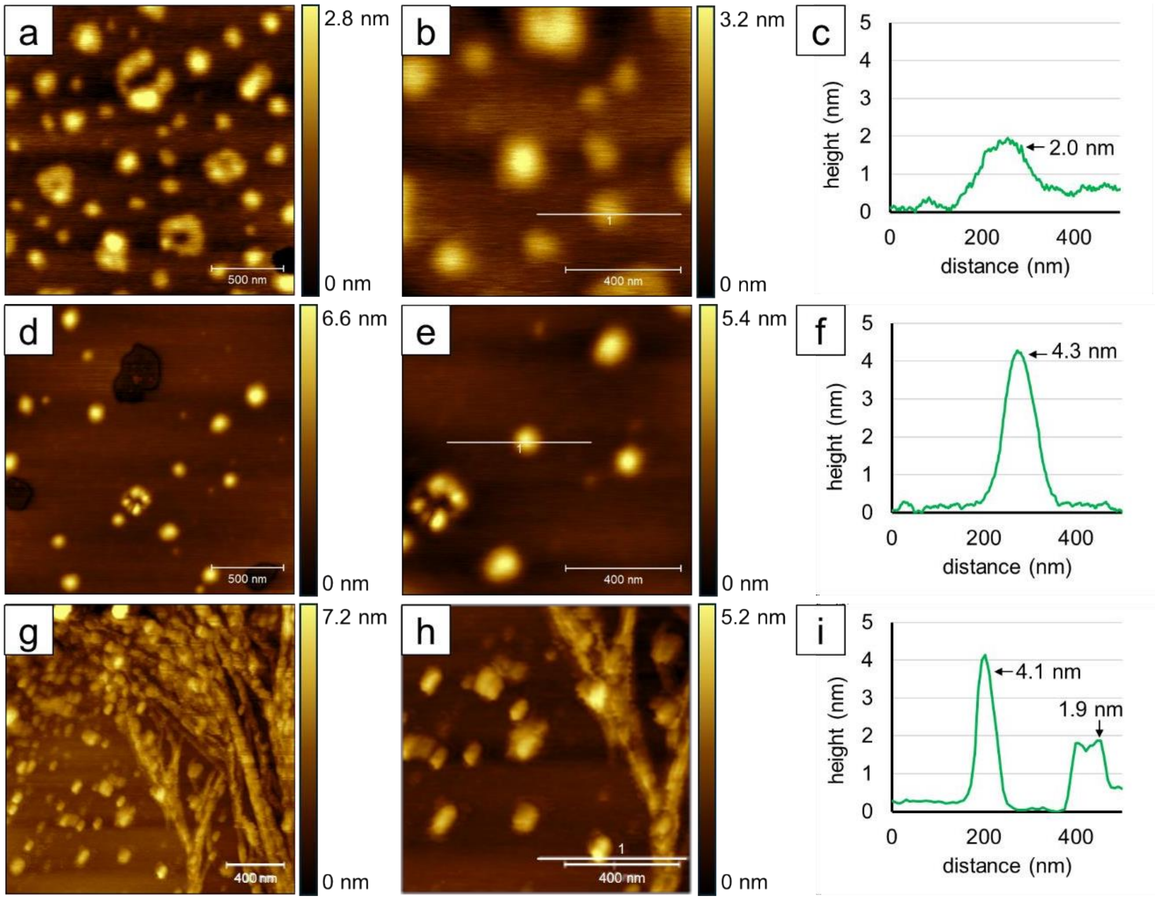

Fig. S11: Topography AFM results for BODIPYs **1b, 2b**, and **7b** dissolved in 80% water and 20% acetonitrile. For BODIPY **1b**: (a) Topography frame, 2×2 µm²; (b) zoom-in view, 1×1 µm²; (c) cursor profile for the line in b. For **2b**: (d) a 2×2 µm² image (e) a 1×1 µm² zoom-in view; (f) cursor profile for the line in e. For **7b**: (g) Topography frame, 2×2 µm²; (h) zoom-in view, 1×1 µm²; (i) cursor profile for the line in h. Images acquired using tapping-mode AFM in ambient conditions.

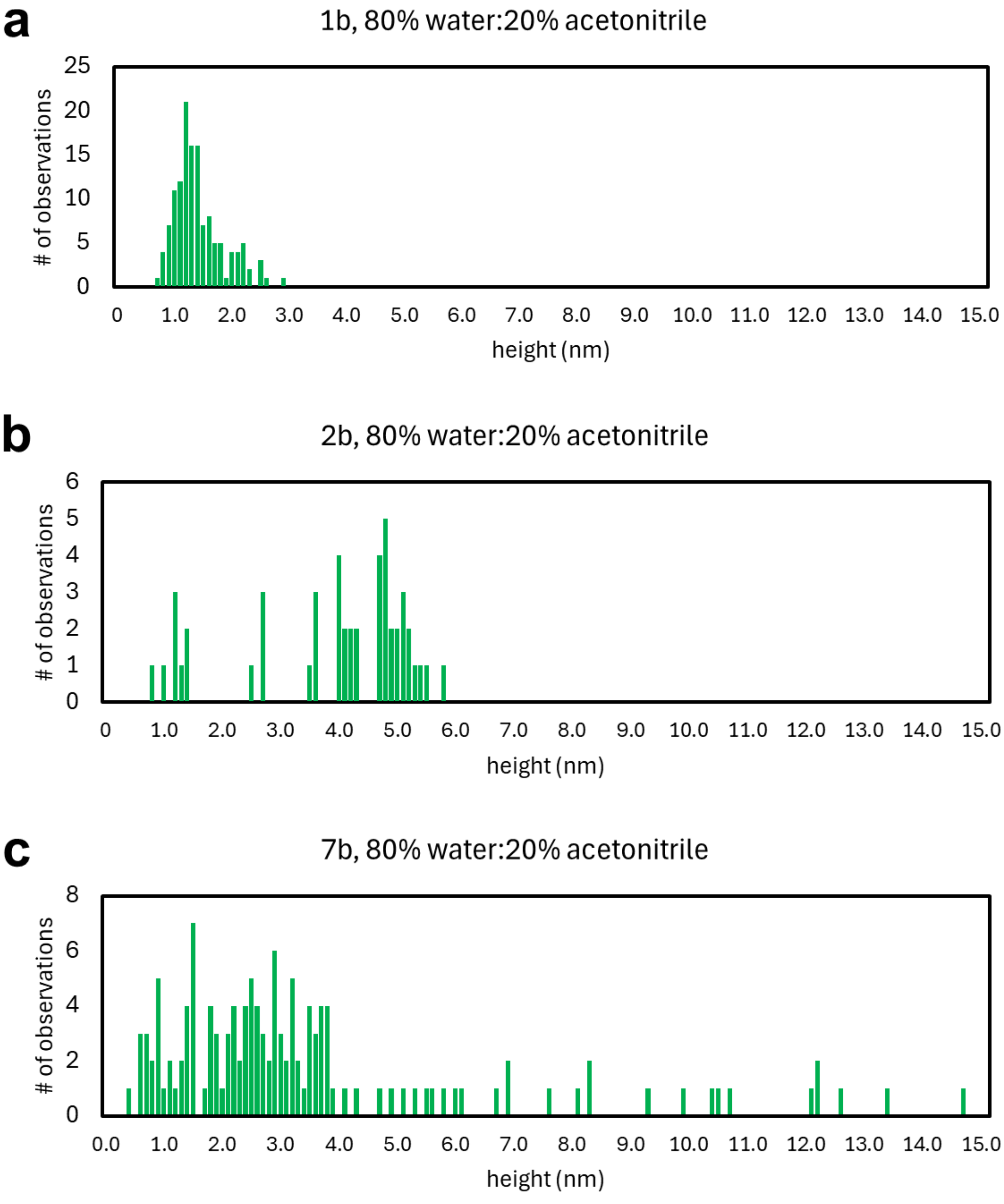

Fig. S12: Size analysis for aggregates of BODIPYs **1b** (a), **2b** (b) and **7b** (c) for samples that were dissolved in 80% water and 20% acetonitrile. Results obtained from AFM cursor height measurements.

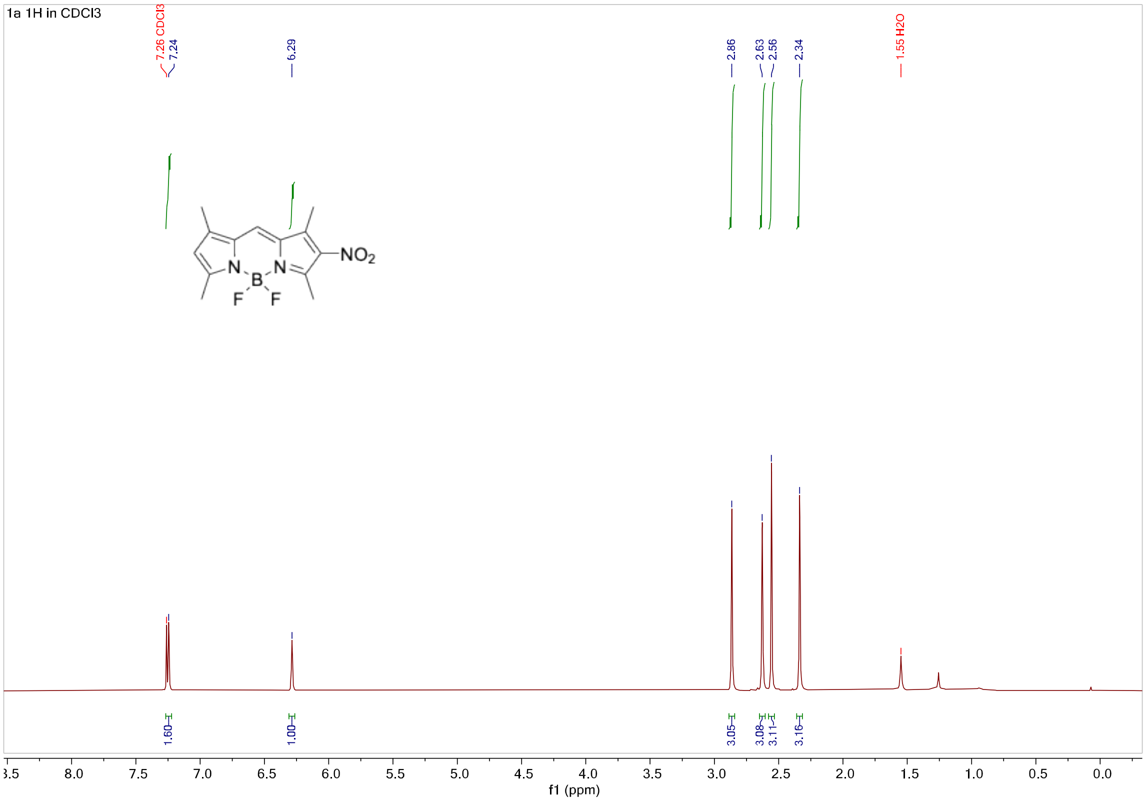

Fig. S13. ^{1}H NMR spectrum of BODIPY **1a** in $CDCl_3$

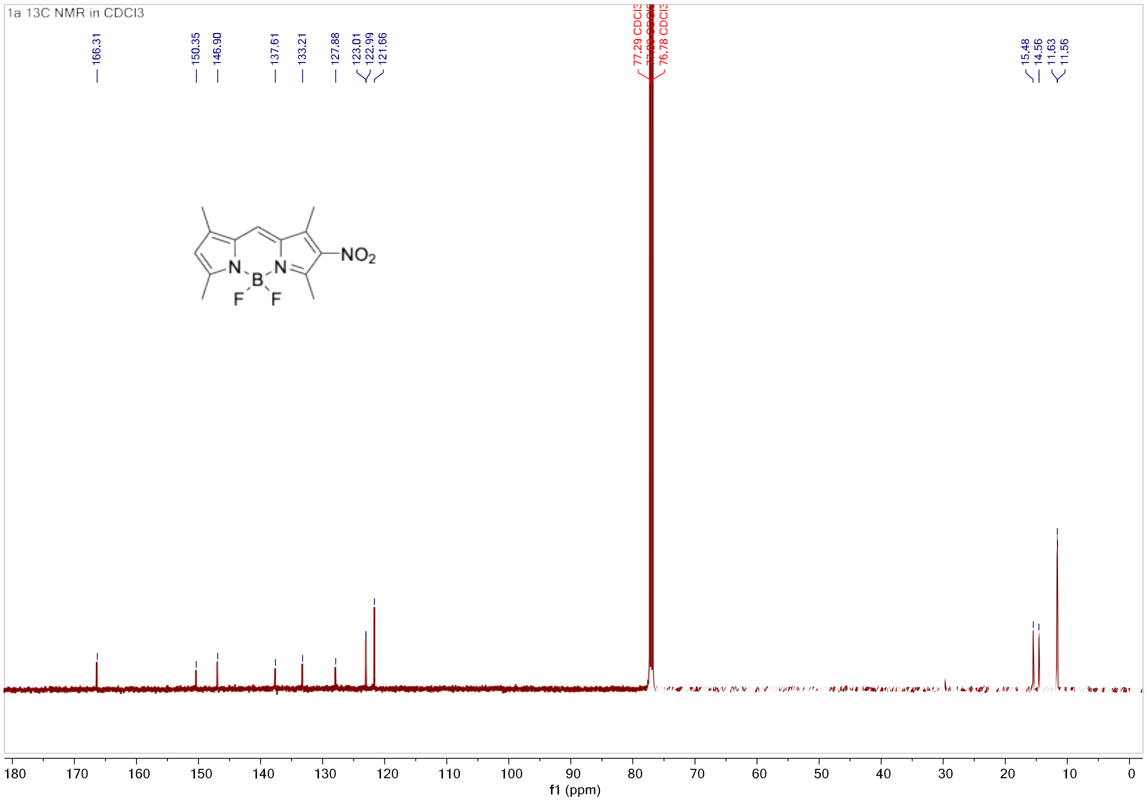

Fig. S14. ^{13}C NMR spectrum of BODIPY **1a** in $CDCl_3$

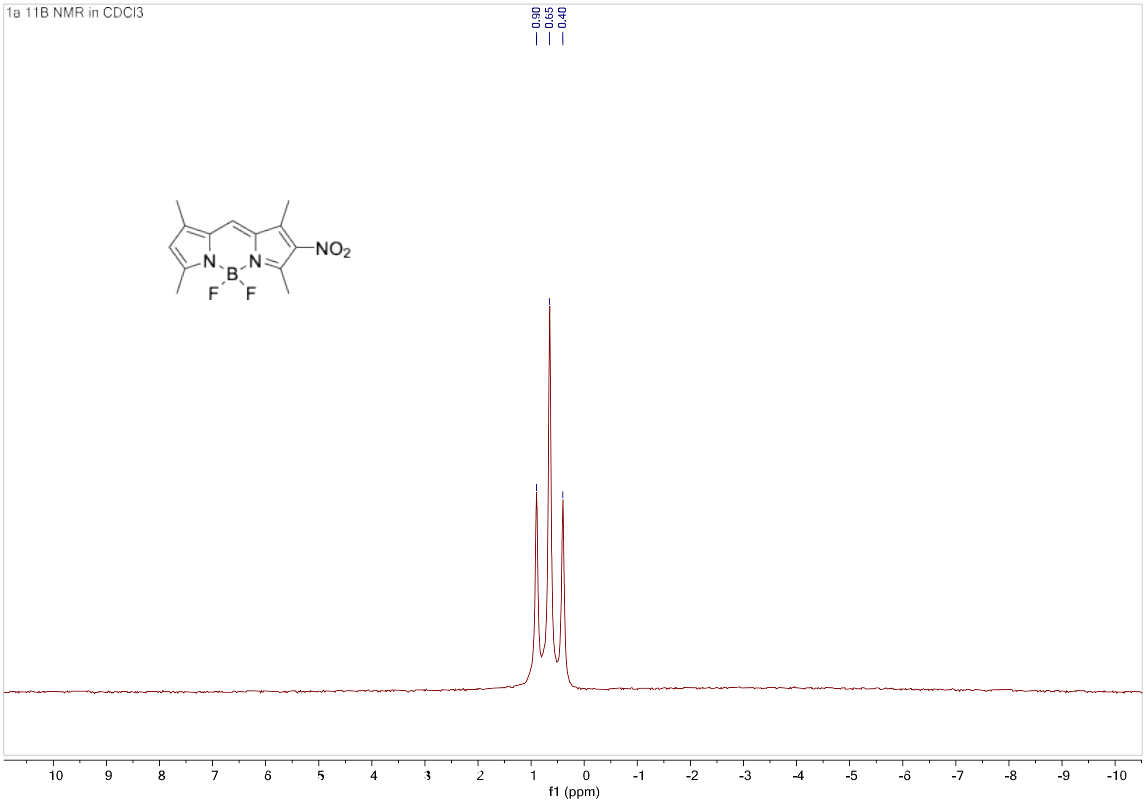

Fig. S15. ^{11}B NMR spectrum of BODIPY **1a** in $CDCl_3$

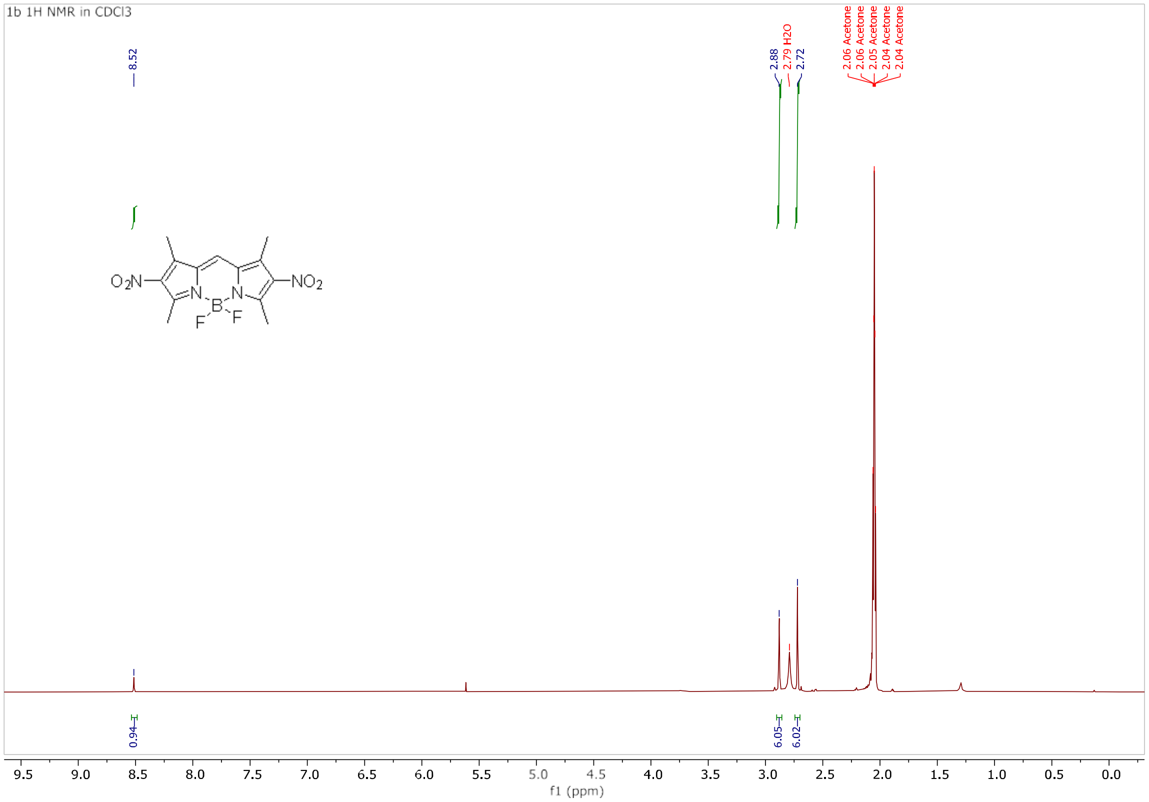

Fig. S16. ^{1}H NMR spectrum of BODIPY **1b** in acetone-d_6

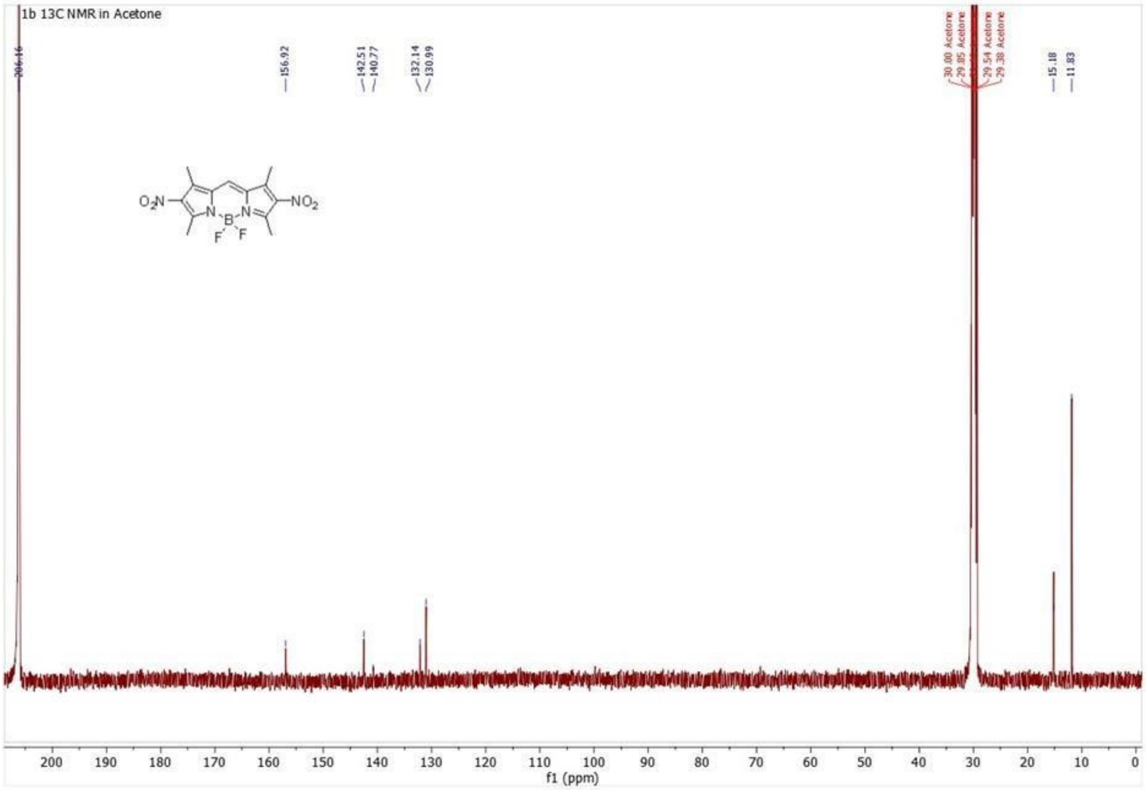

Fig. S17. ^{13}C NMR spectrum of BODIPY **1b** in acetone-d_6

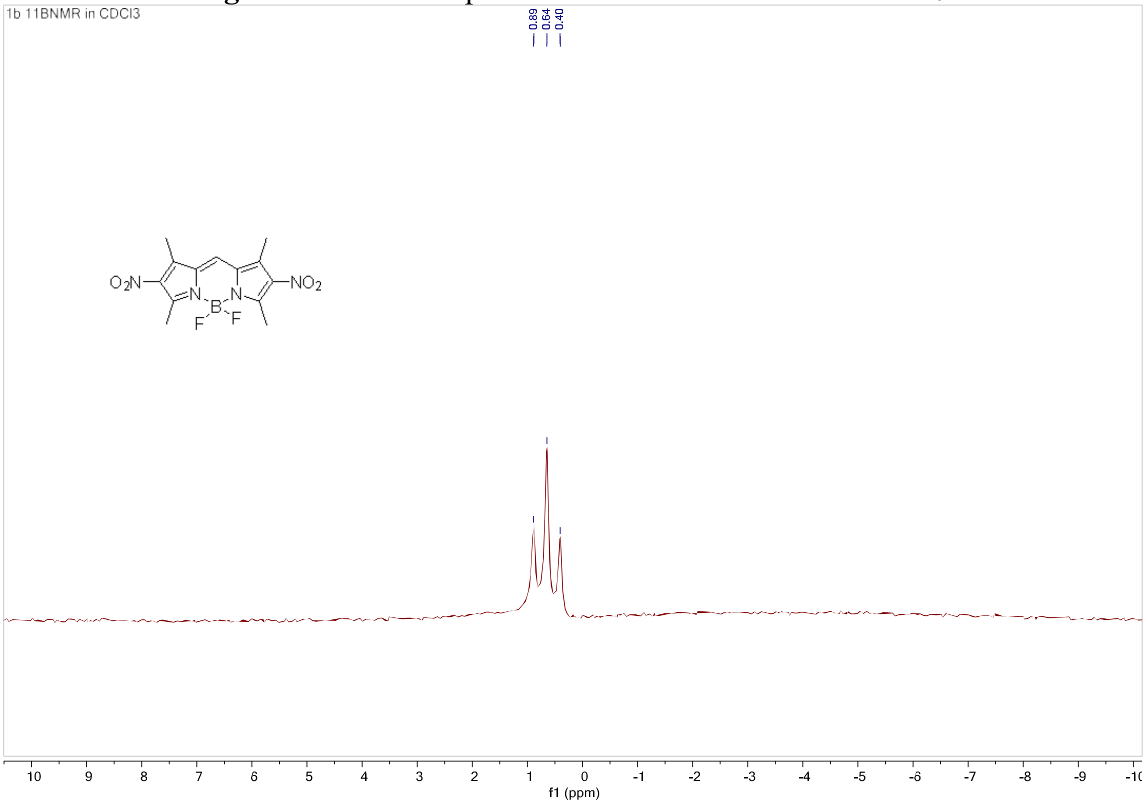

Fig. S18. ^{11}B NMR spectrum of BODIPY **1b** in $CDCl_3$.

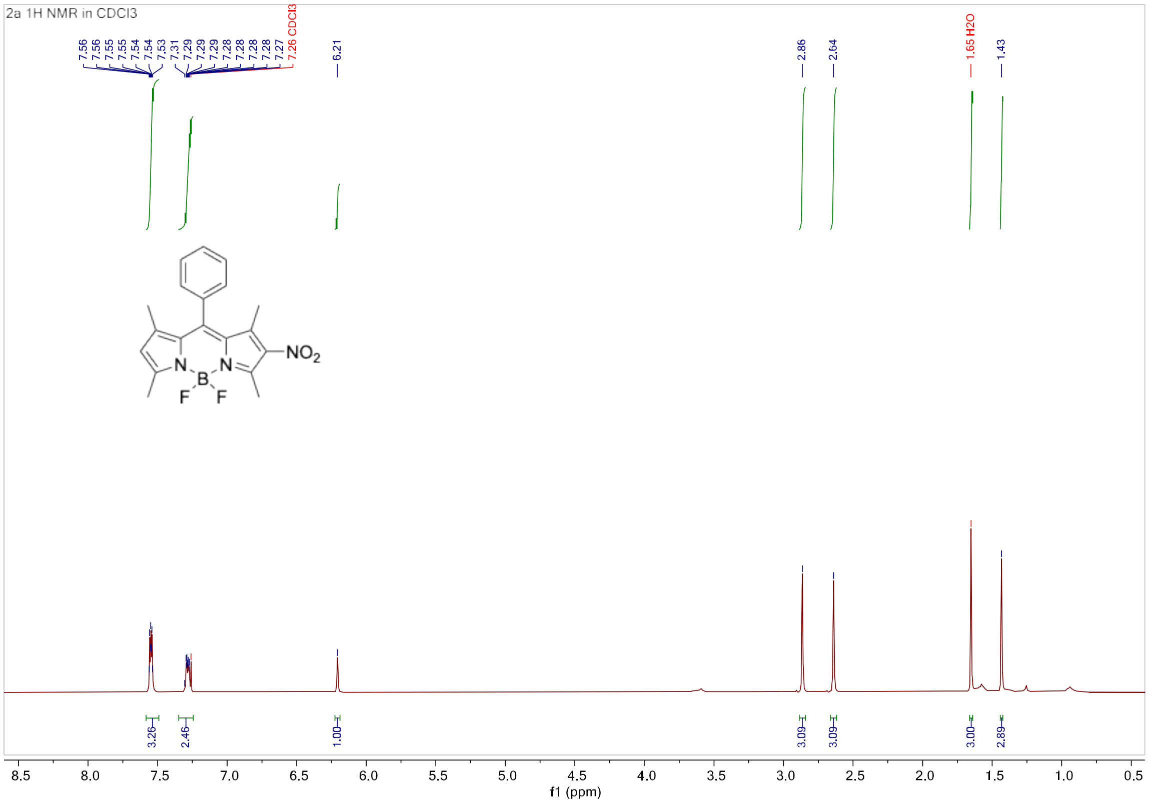

Fig. S19. ^{1}H NMR spectrum of BODIPY **2a** in $CDCl_3$

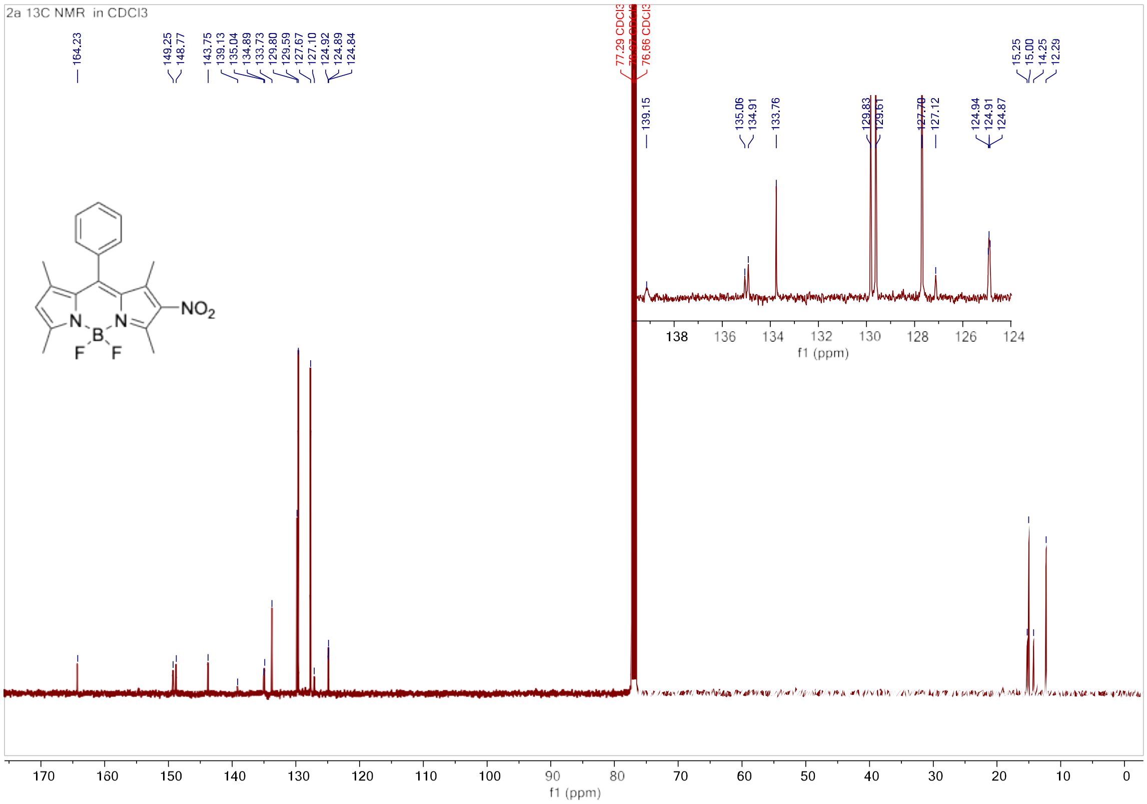

Fig. S20. ^{13}C NMR spectrum of BODIPY **2a** in $CDCl_3$

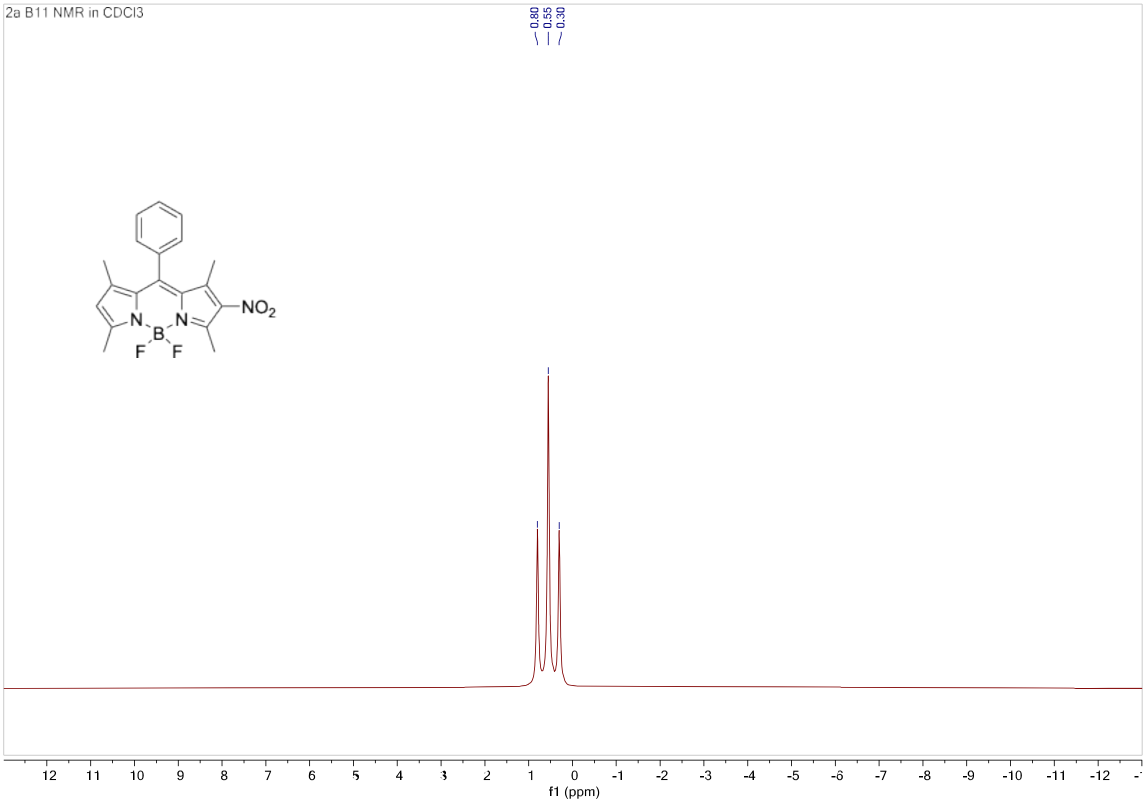

Fig. S21. [11] B NMR spectrum of BODIPY **2a** in $CDCl_3$

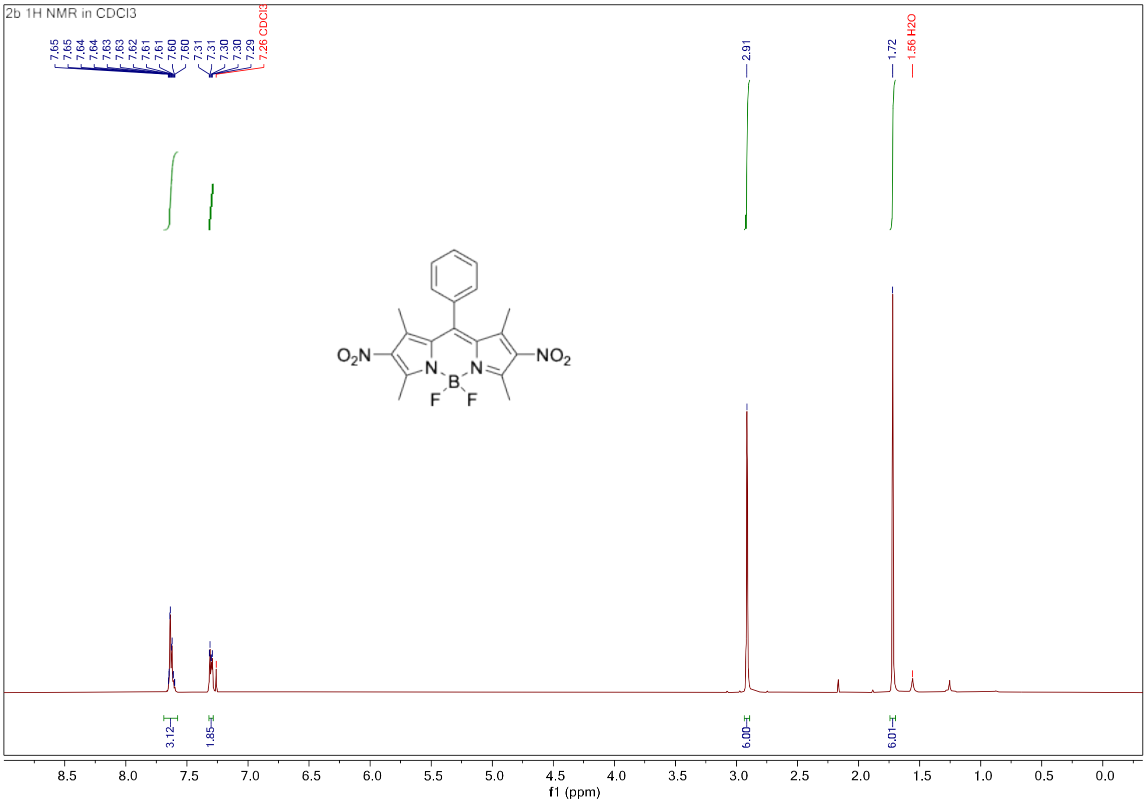

Fig. S22. ^{1}H NMR spectrum of BODIPY **2b** in $CDCl_3$

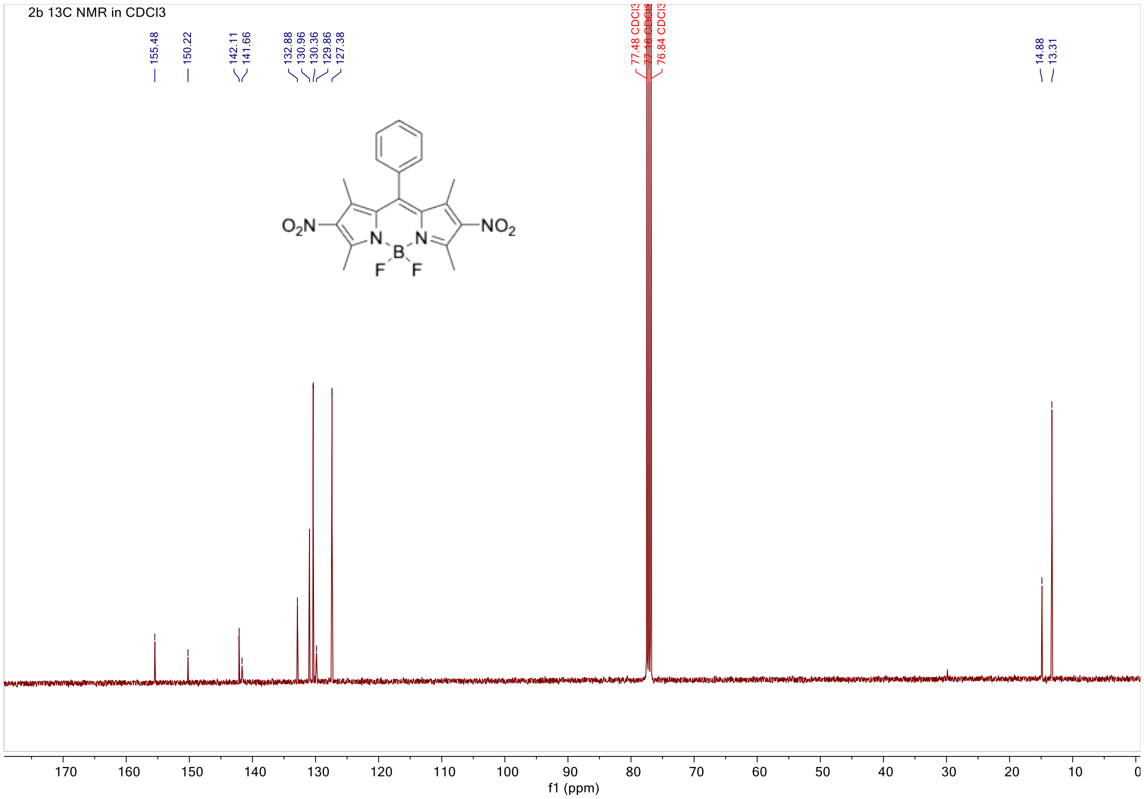

Fig. S23.^{13}C NMR spectrum of BODIPY **2b** in $CDCl_3$

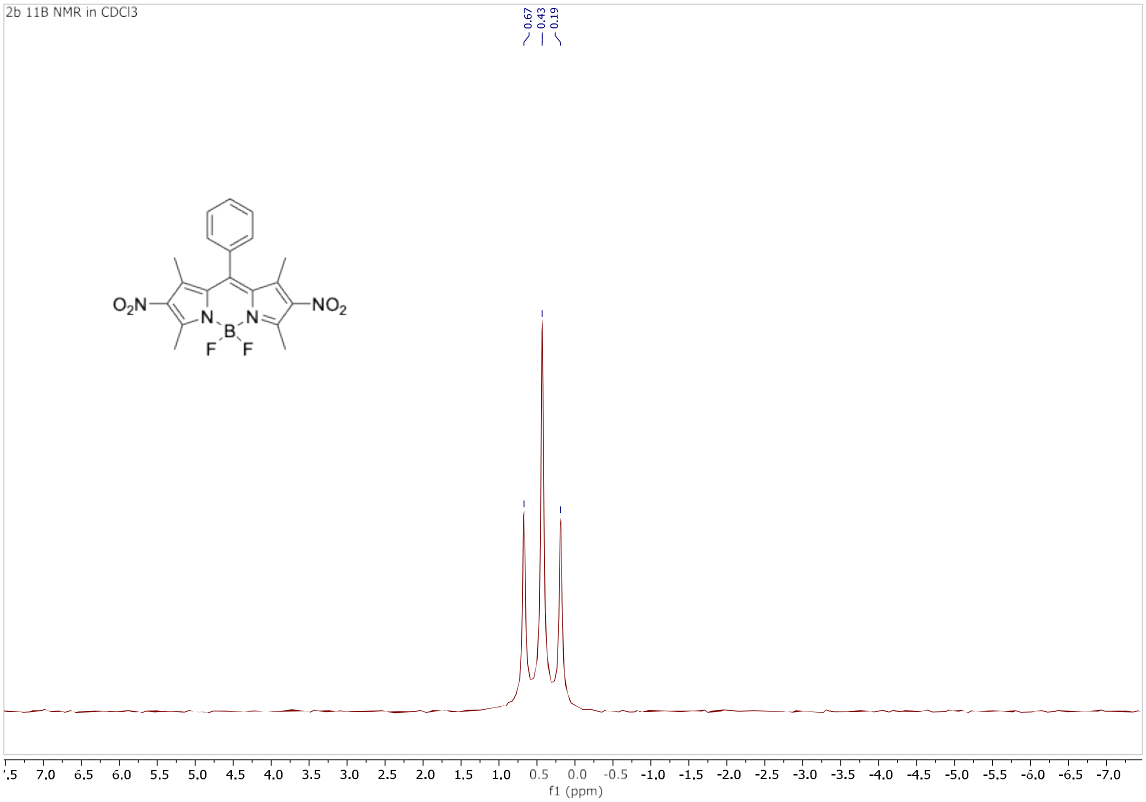

Fig. S24. ^{11}B NMR spectrum of BODIPY **2b** in $CDCl_3$

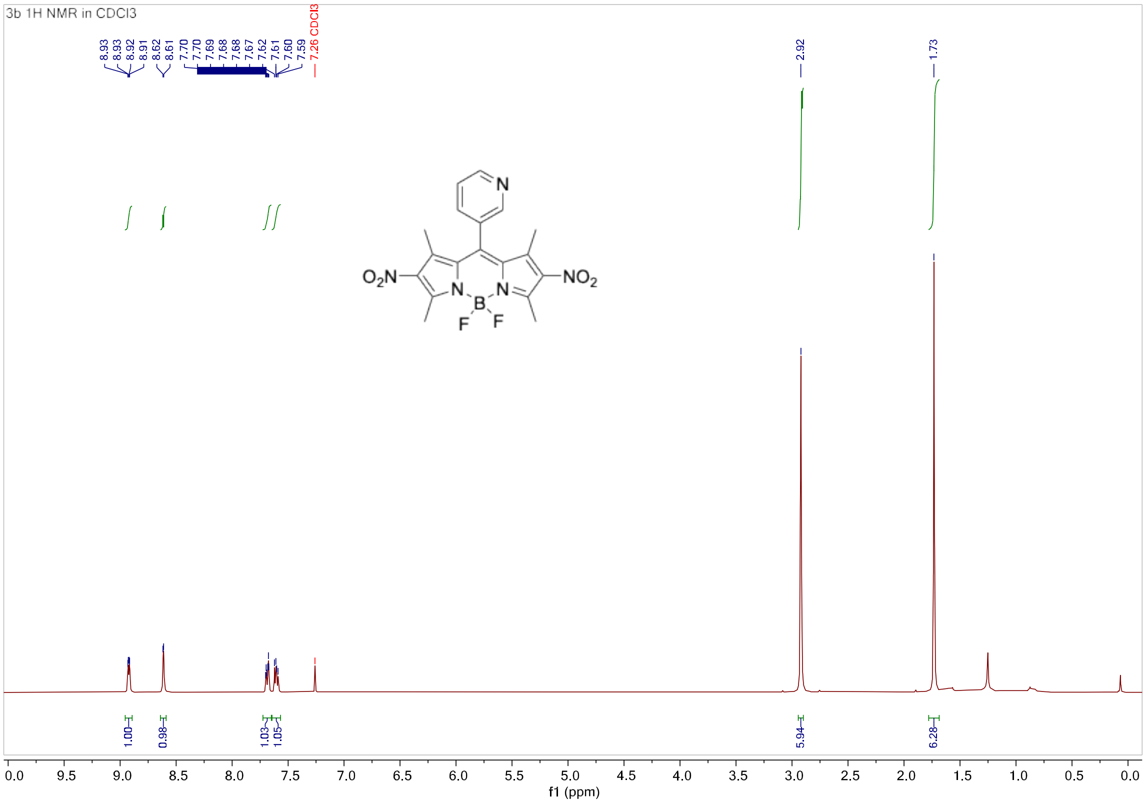

Fig. S25. ^{1}H NMR spectrum of BODIPY **3b** in $CDCl_3$

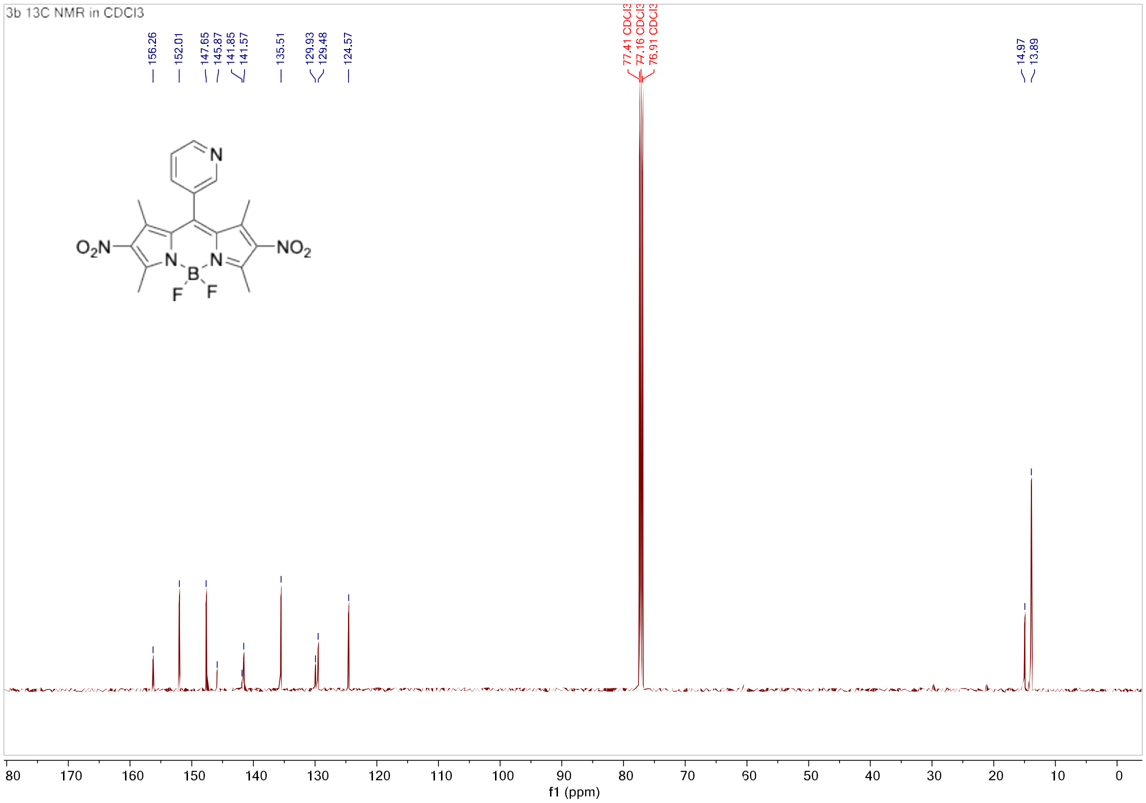

Fig. S26. ^{13}C NMR spectrum of BODIPY **3b** in $CDCl_3$

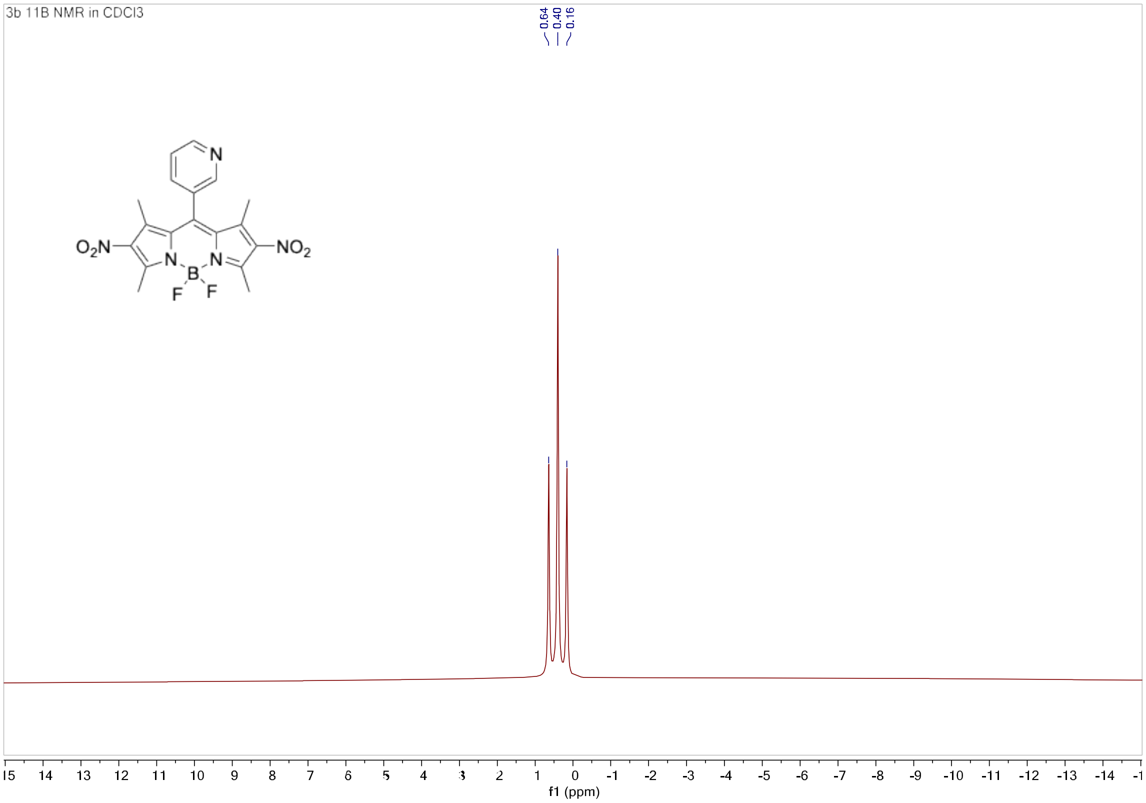

Fig. S27. [11] B NMR spectrum of BODIPY **3b** in $CDCl_3$

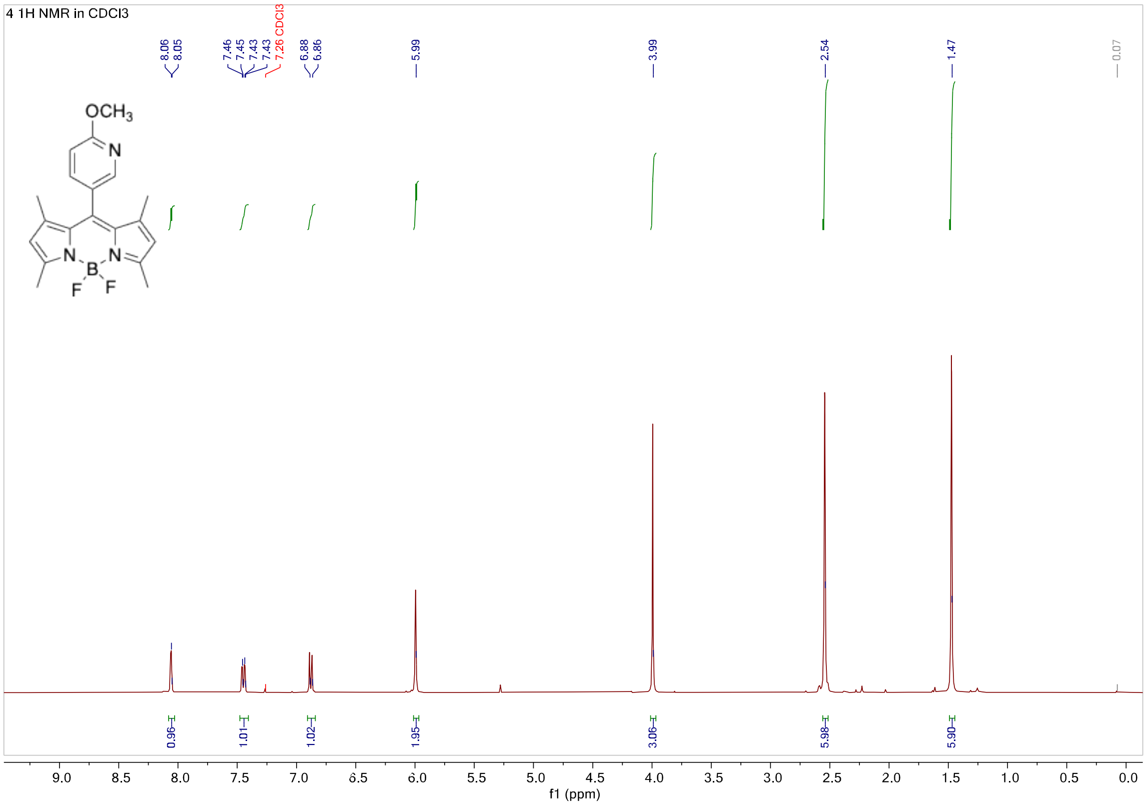

Fig. S28. ^{1}H NMR spectrum of BODIPY **4** in $CDCl_3$

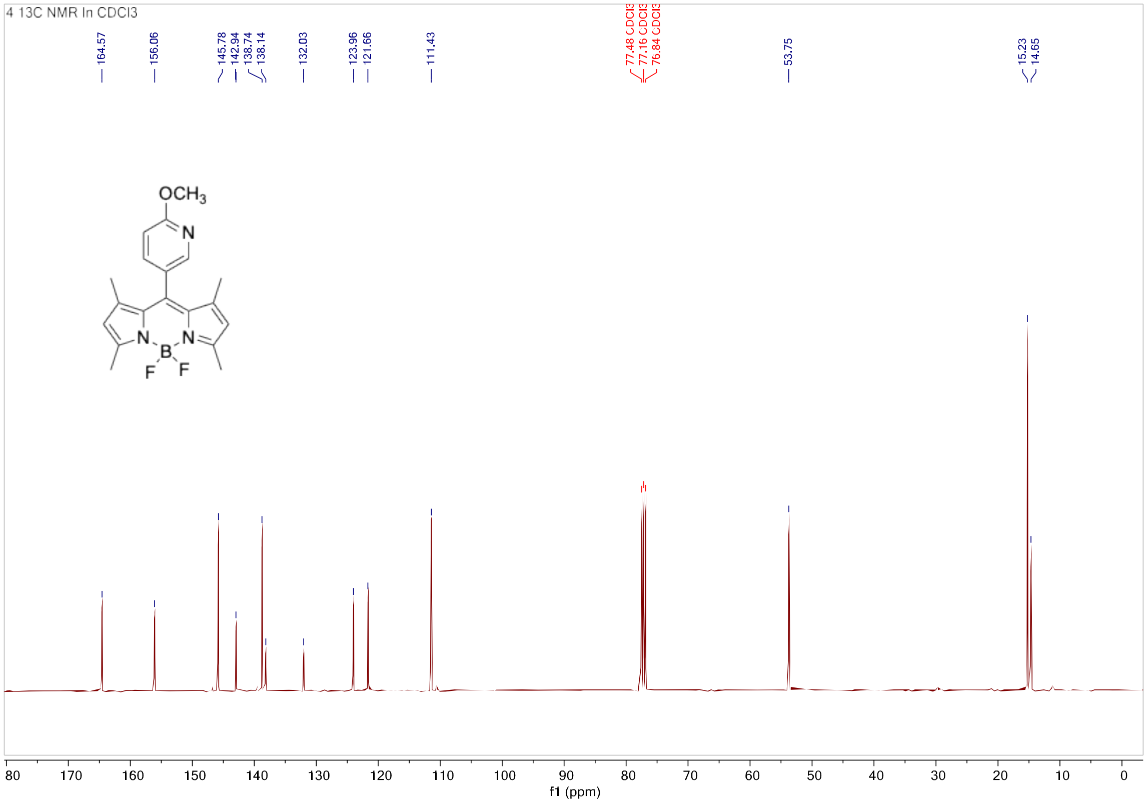

Fig. S29. ^{13}C NMR spectrum of BODIPY **4** in $CDCl_3$

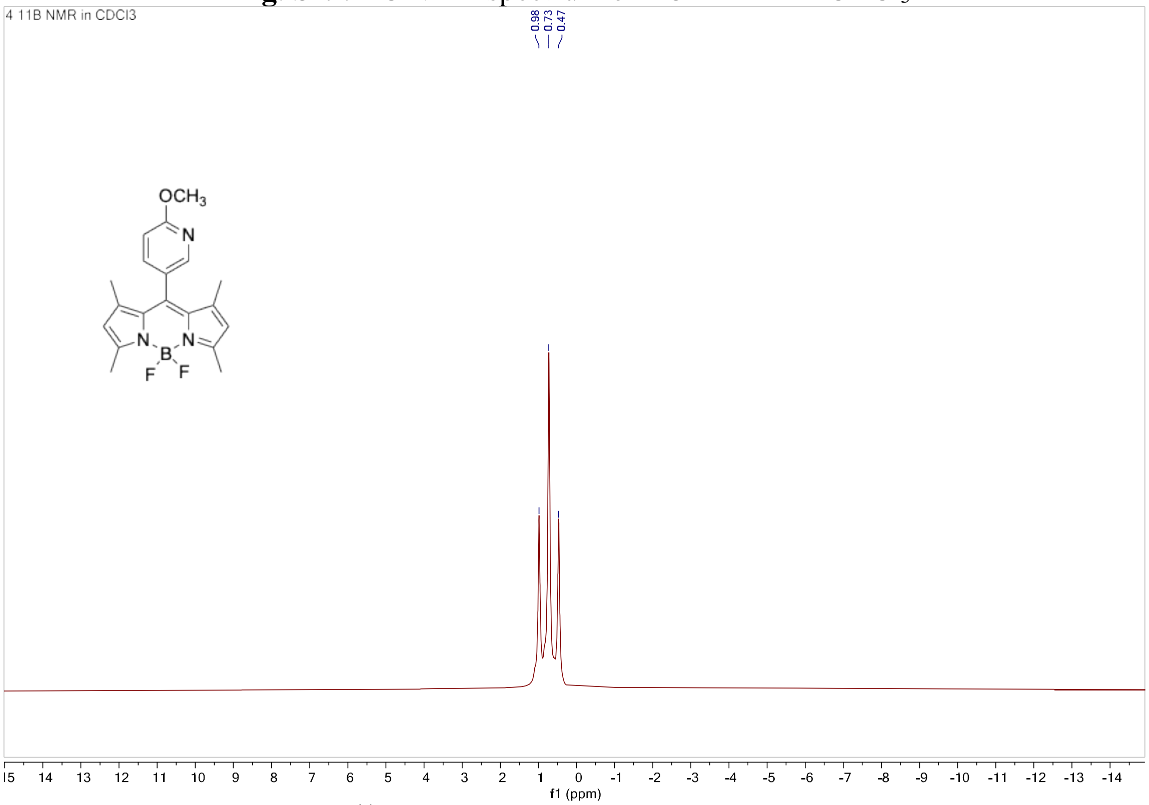

Fig. S30. ^{11} B NMR spectrum of BODIPY **4** in $CDCl_3$

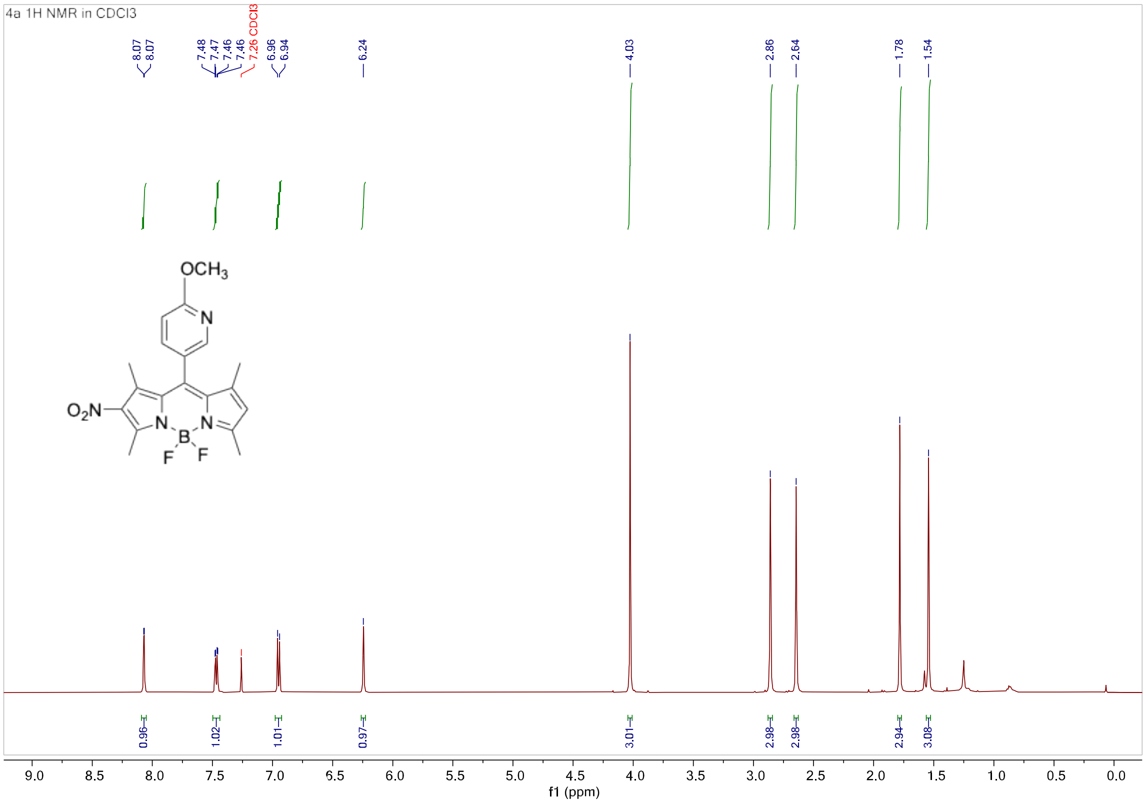

Fig. S31. ^{1}H NMR spectrum of BODIPY **4a** in $CDCl_3$

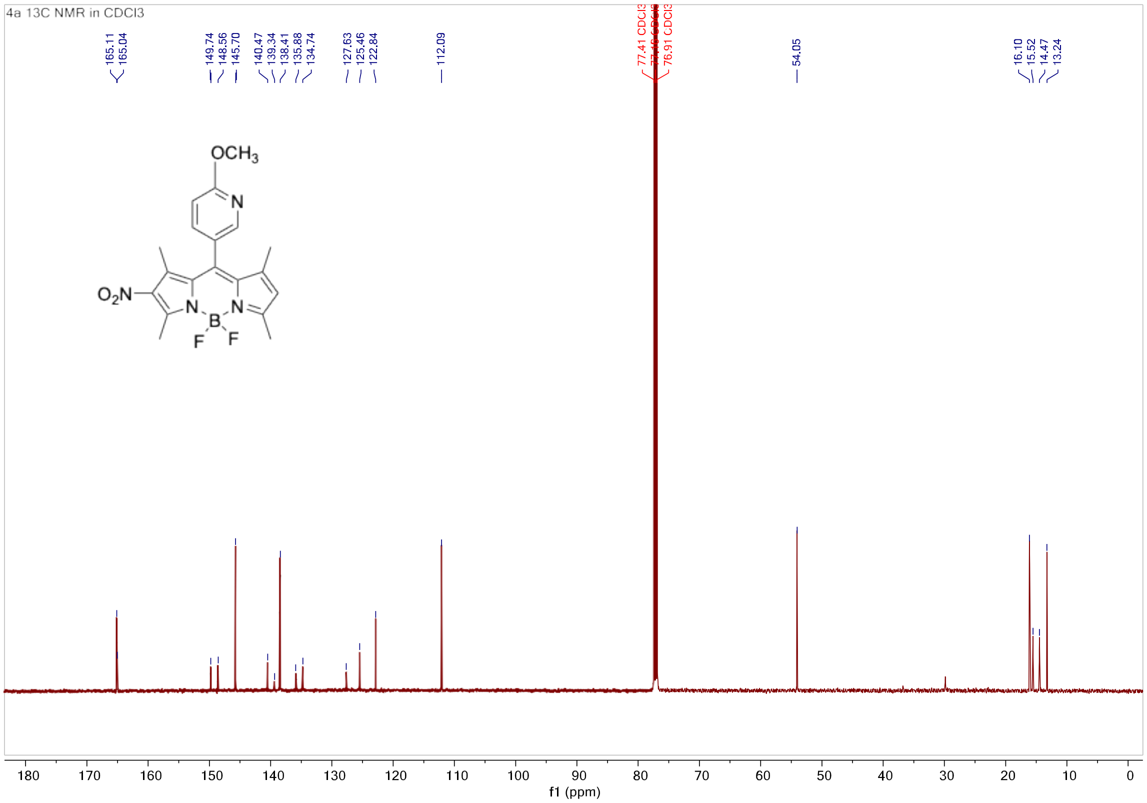

Fig. S32. ^{13}C NMR spectrum of BODIPY **4a** in $CDCl_3$

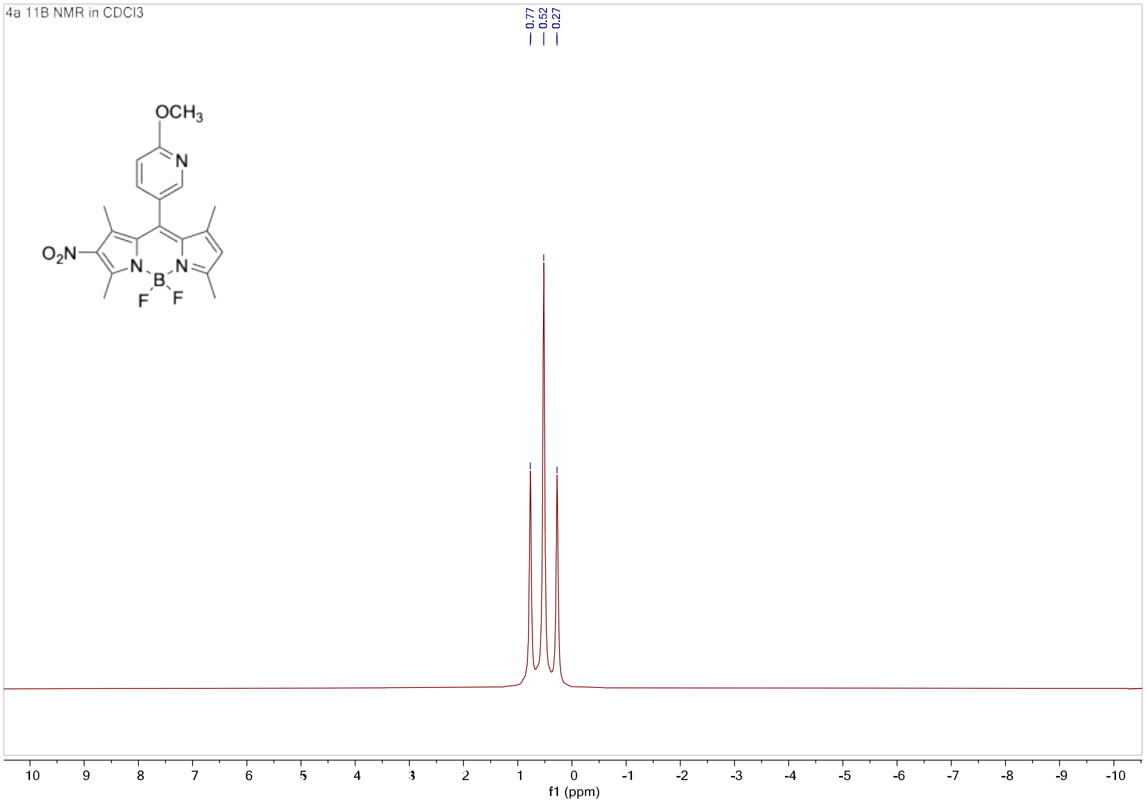

Fig. S33. [11] B NMR spectrum of BODIPY **4a** in $CDCl_3$

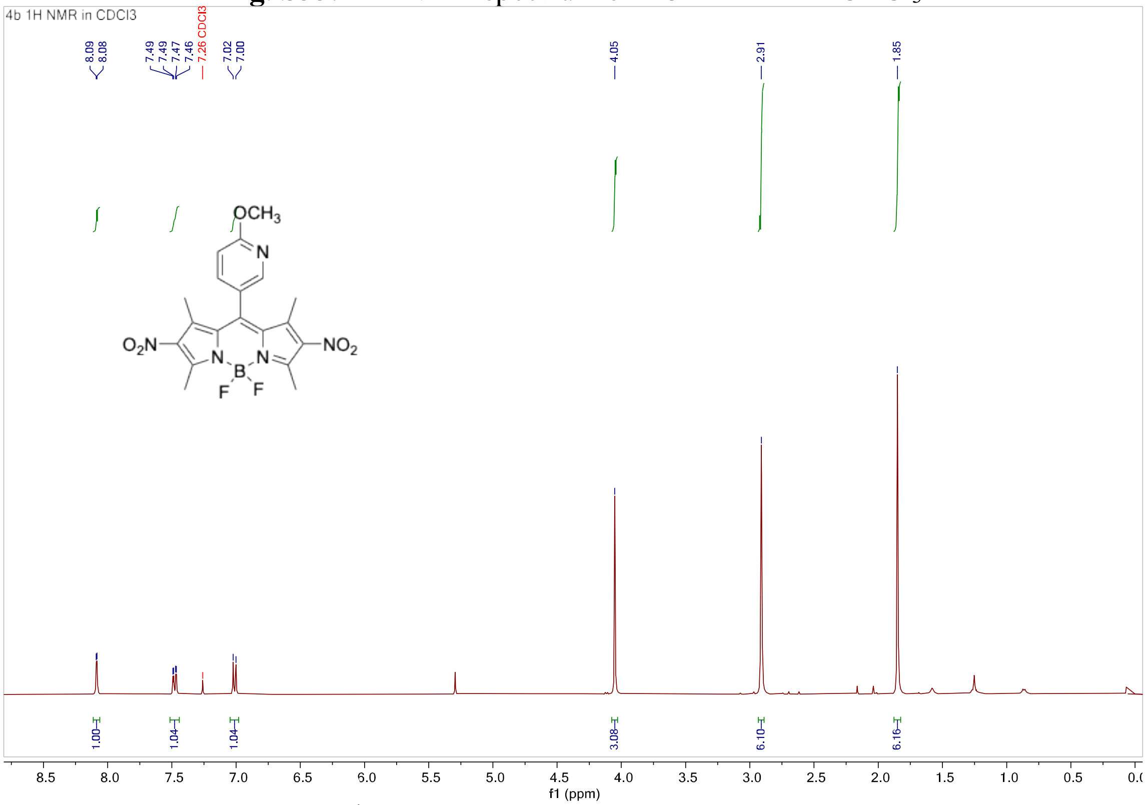

Fig. S34. ^{1}H NMR spectrum of BODIPY **4b** in $CDCl_3$

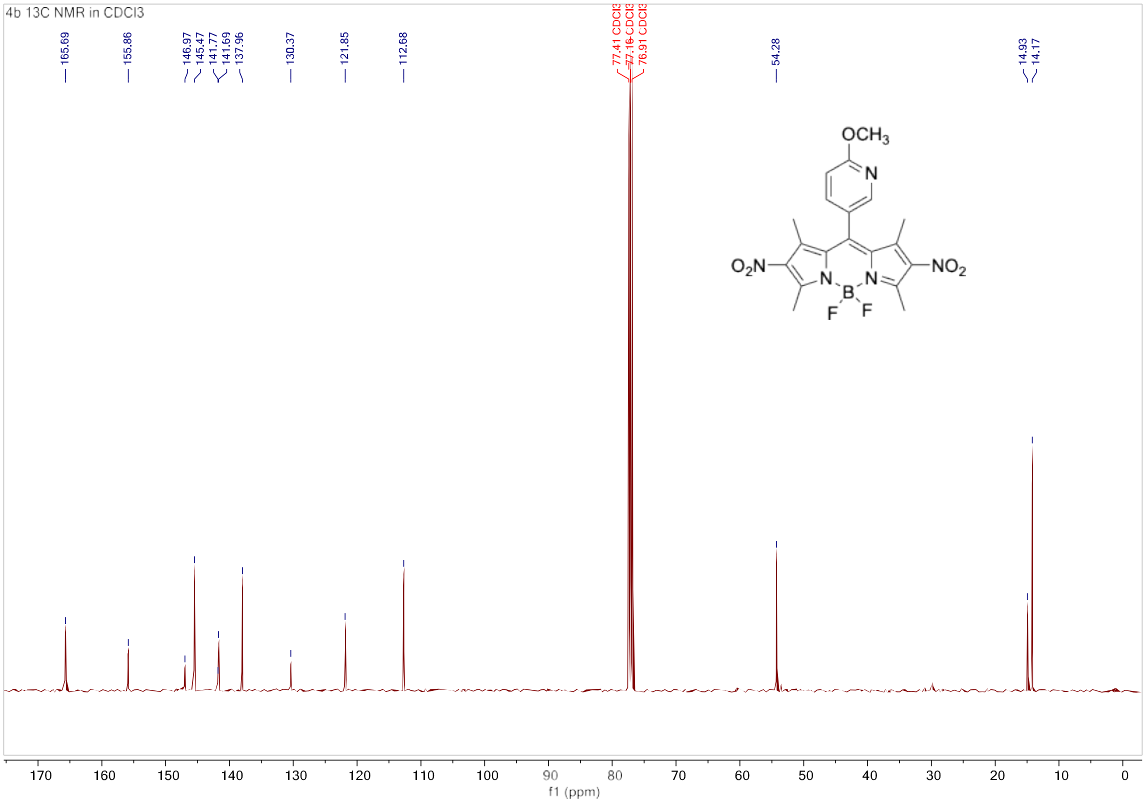

Fig. S35. ^{13}C NMR spectrum of BODIPY **4b** in $CDCl_3$

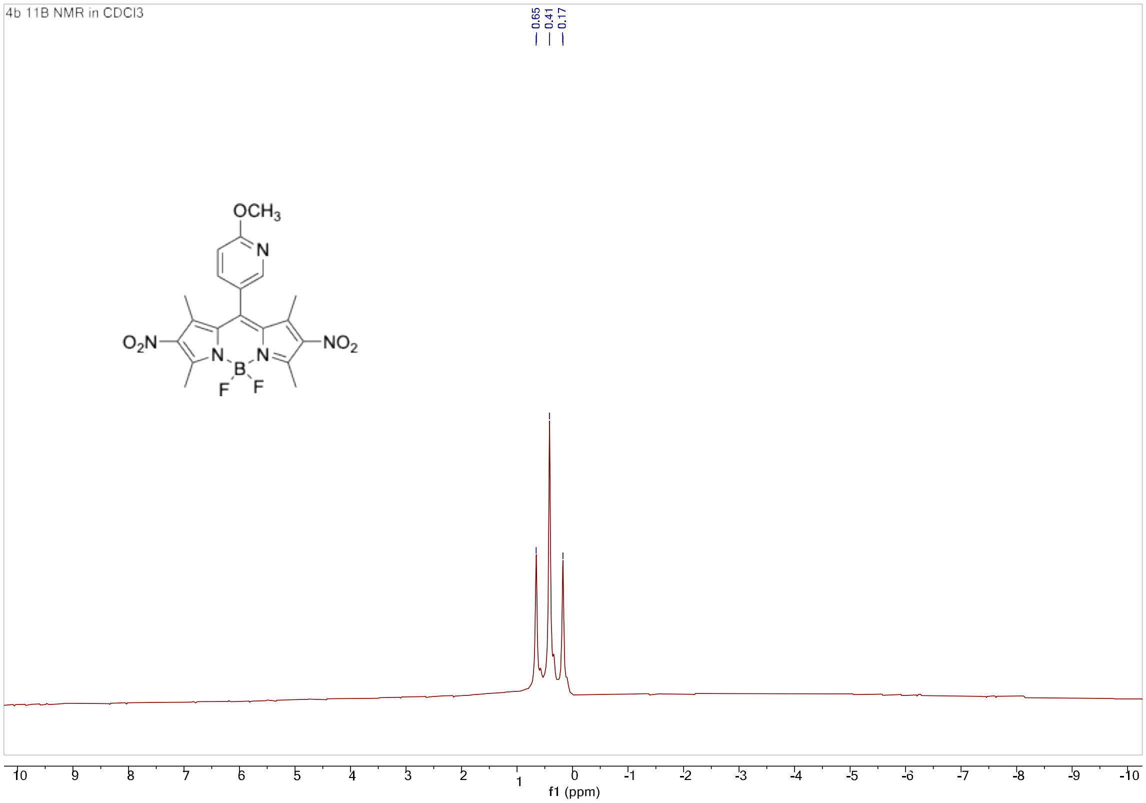

Fig. S36. ^{11}B NMR spectrum of BODIPY **4b** in $CDCl_3$

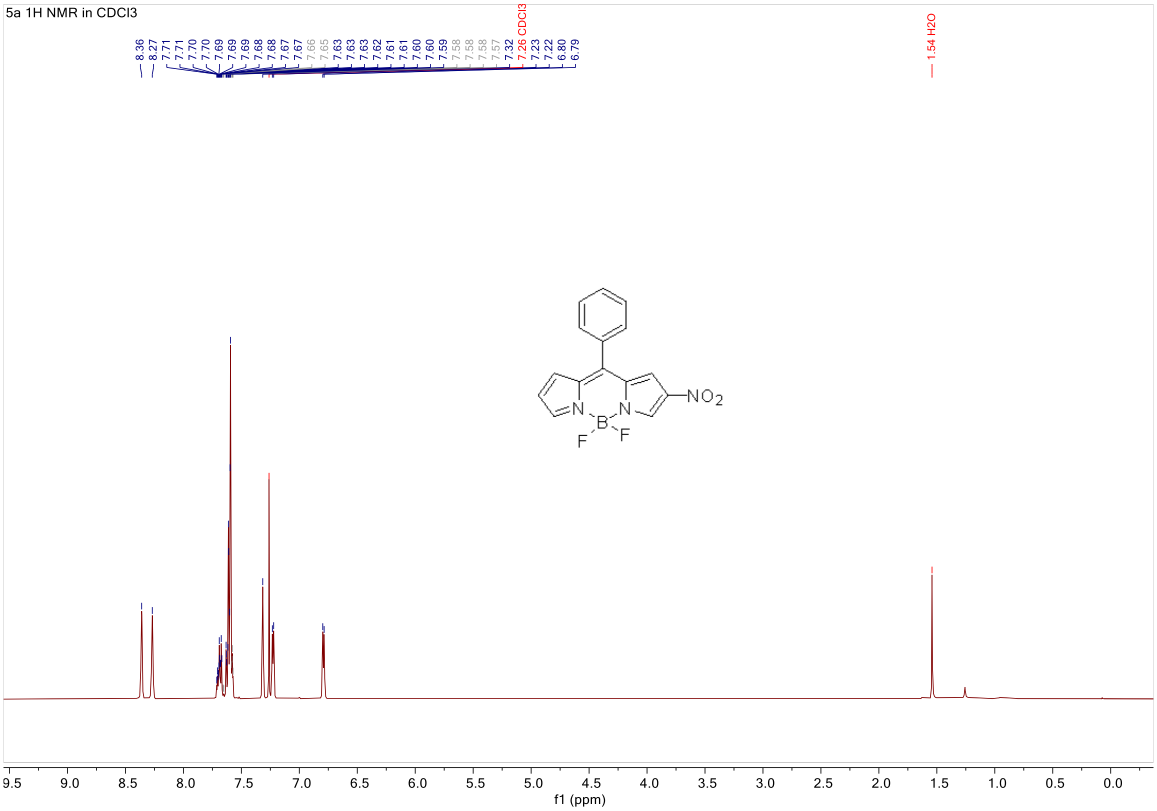

Fig. S37. ^{1}H NMR spectrum of BODIPY **5a** in $CDCl_3$

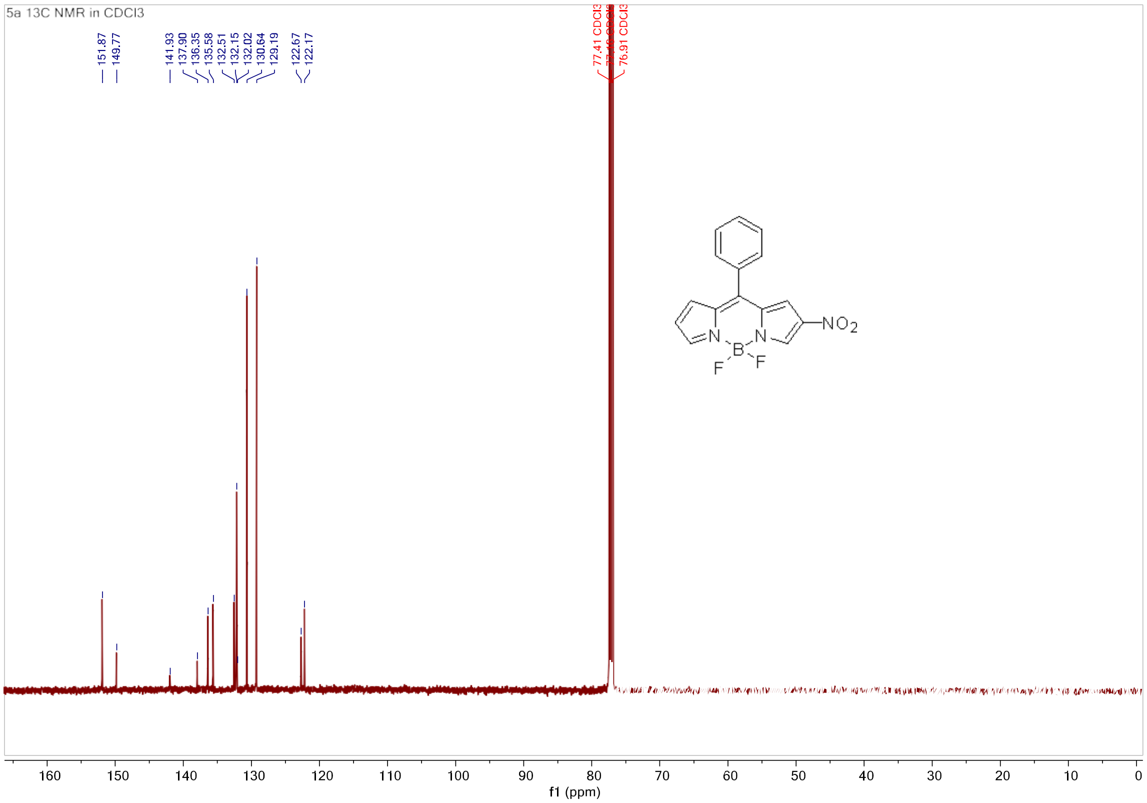

Fig. S38. ^{13}C NMR spectrum of BODIPY **5a** in $CDCl_3$

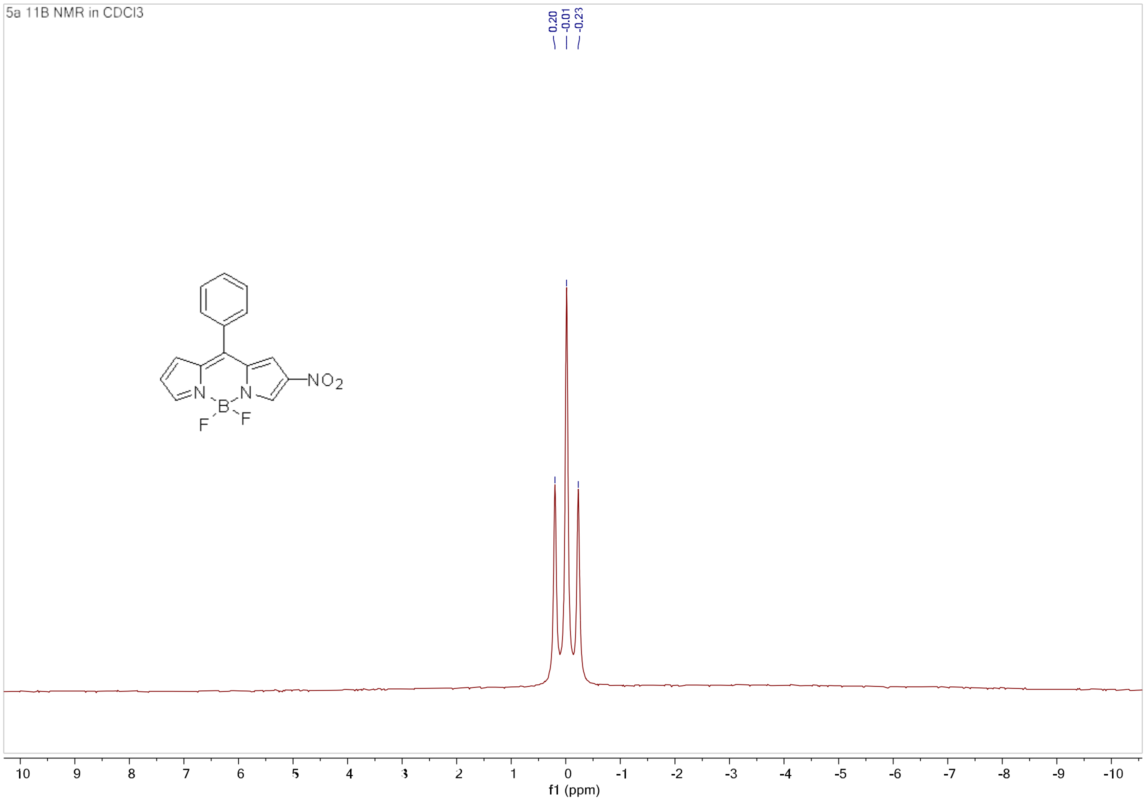

Fig. S39. [11] B NMR spectrum of BODIPY **5a** in $CDCl_3$

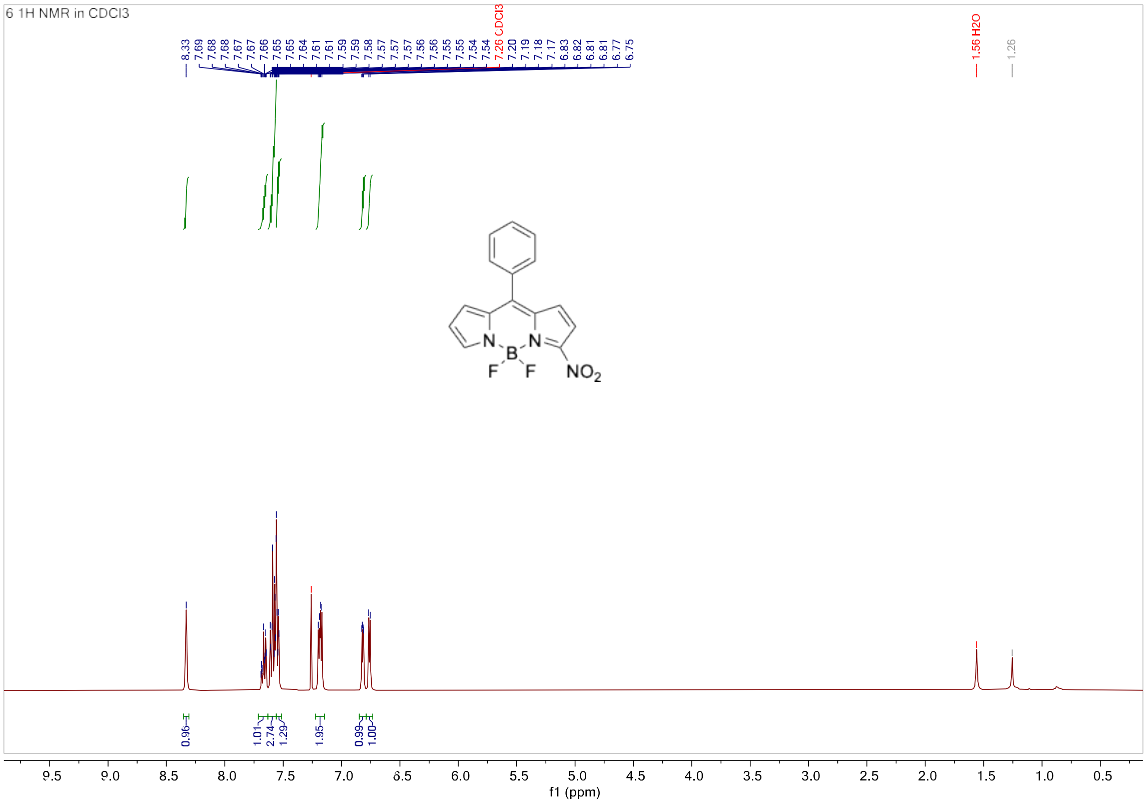

Fig. S40.^{1}H NMR spectrum of BODIPY **6** in $CDCl_3$

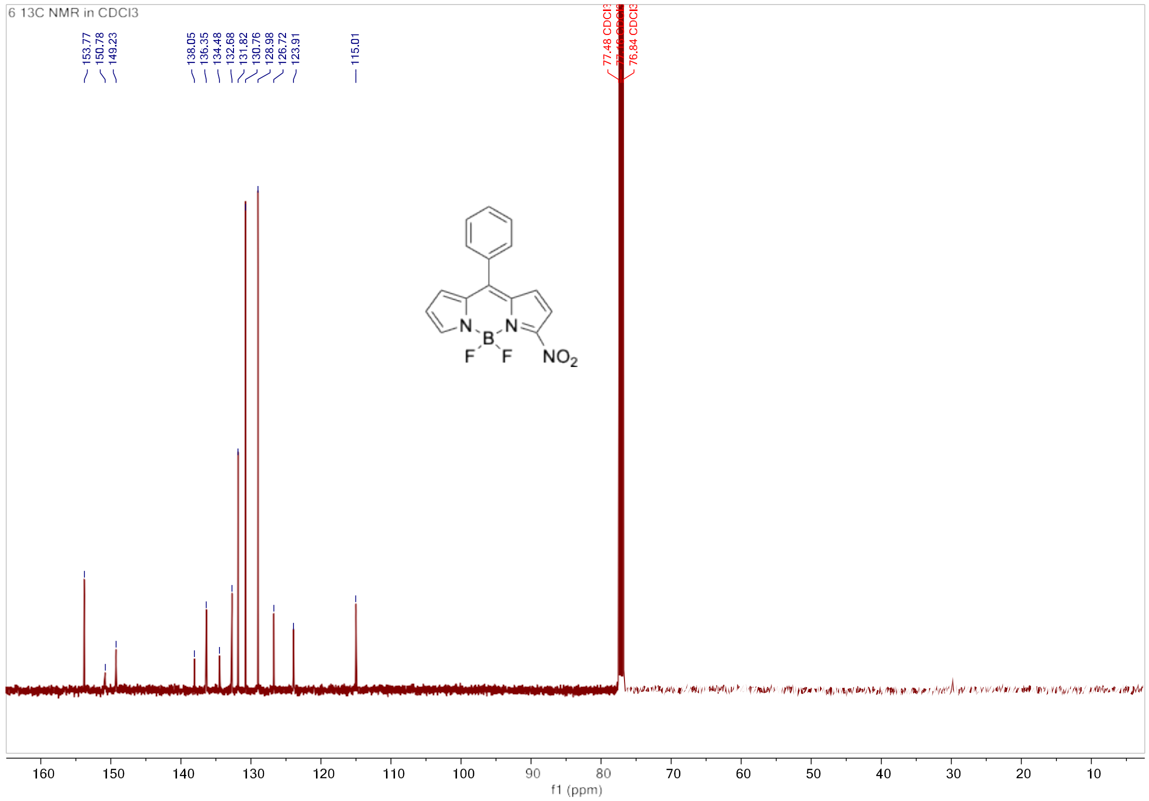

Fig. S41. ^{13}C NMR spectrum of BODIPY **6** in $CDCl_3$

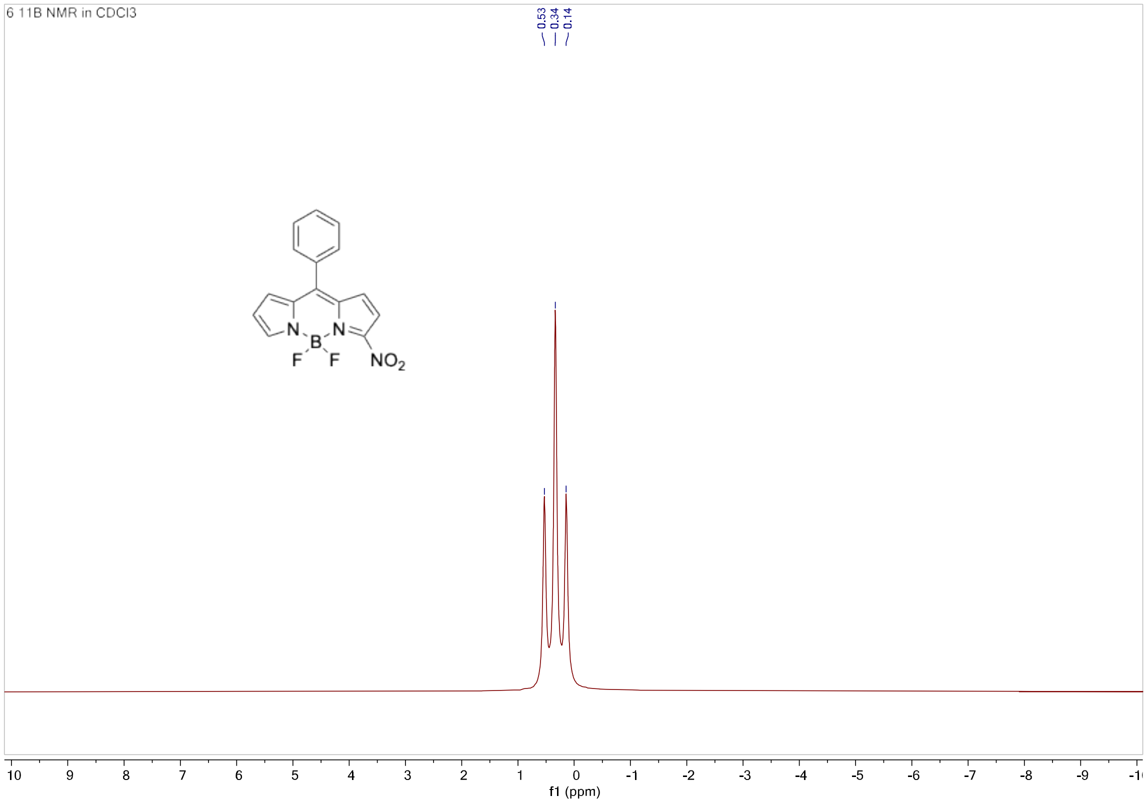

Fig. S42. ^{1}H NMR spectrum of BODIPY **6** in $CDCl_3$

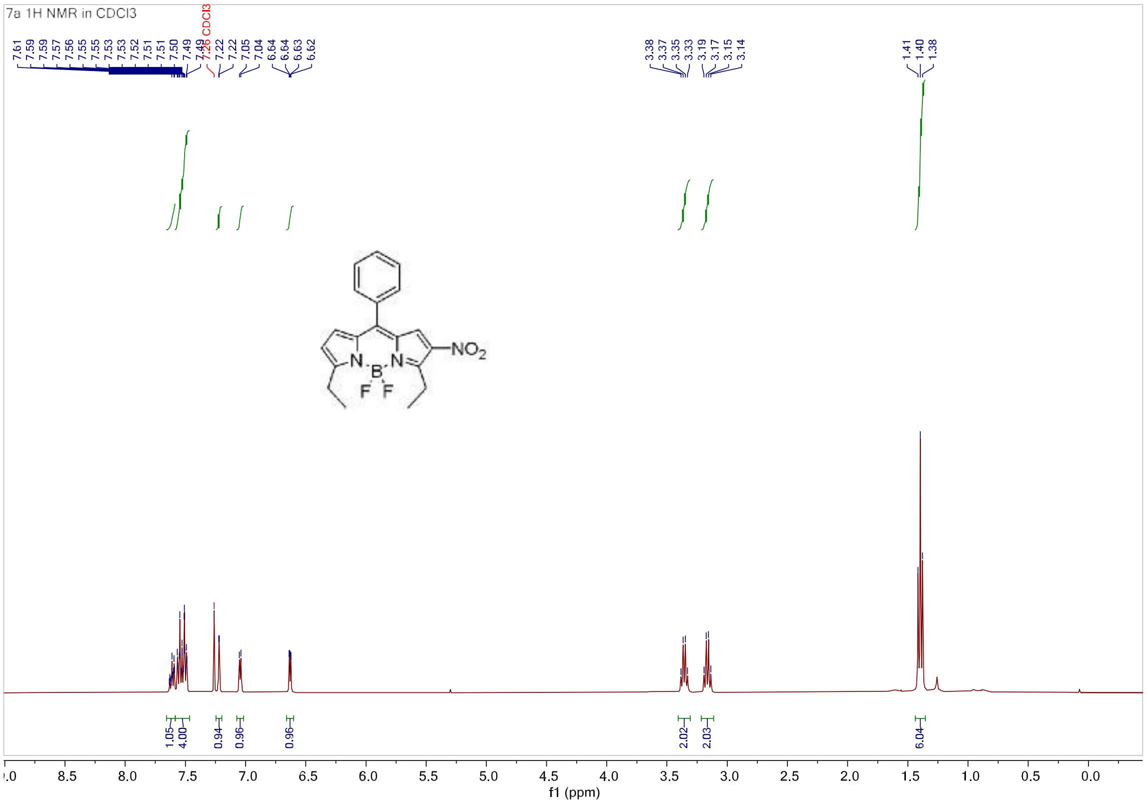

Fig. S43. ^{1}H NMR spectrum of BODIPY **7a** in $CDCl_3$

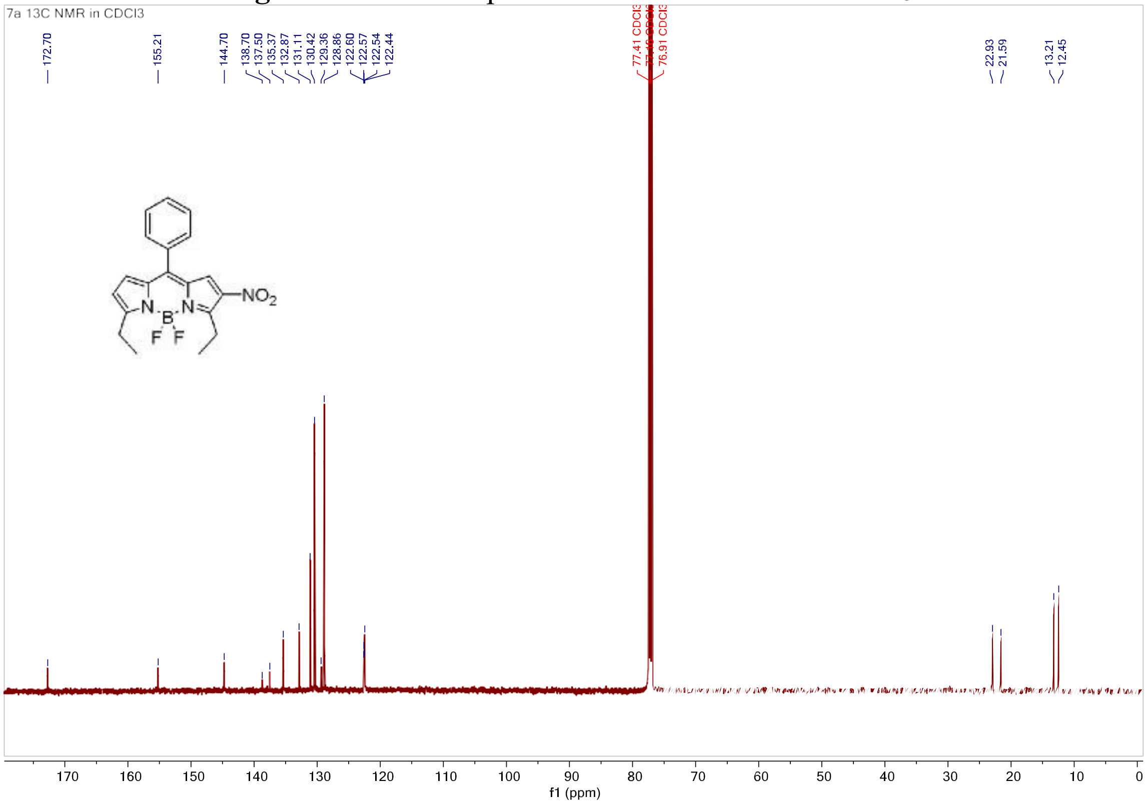

Fig. S44. ^{13}C NMR spectrum of BODIPY **7a** in $CDCl_3$

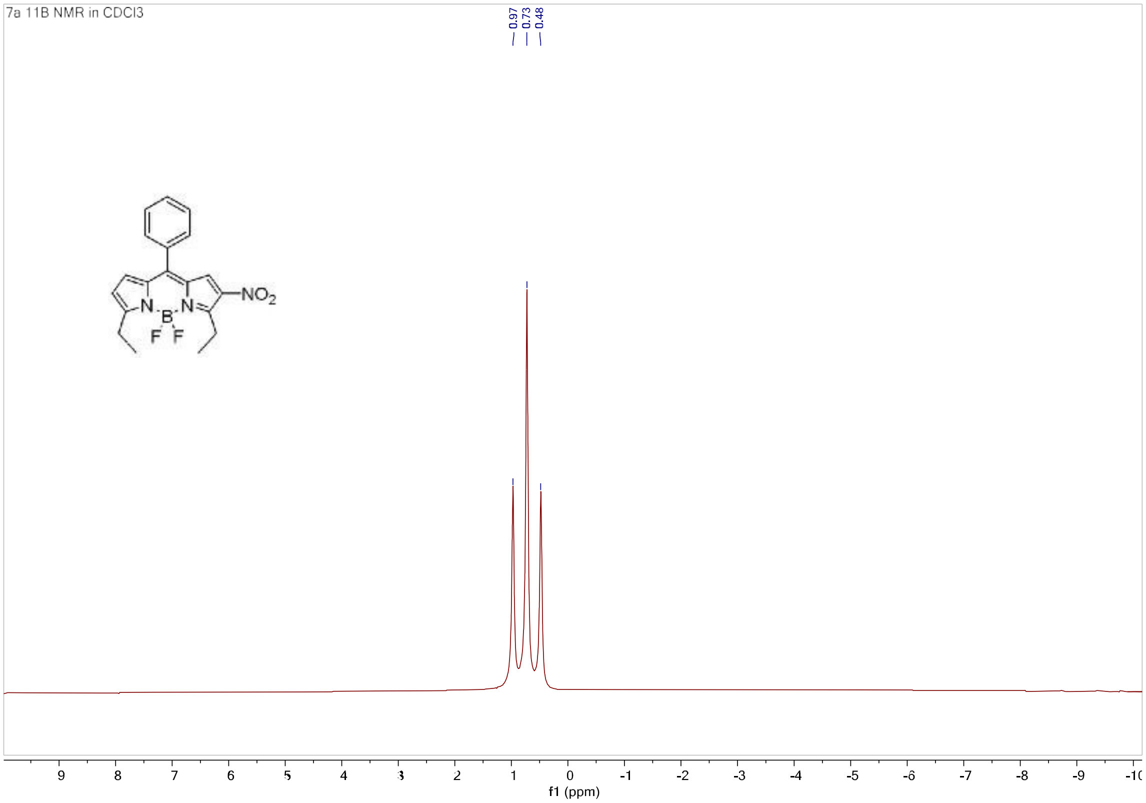

Fig. S45. [11] B NMR spectrum of BODIPY **7a** in $CDCl_3$

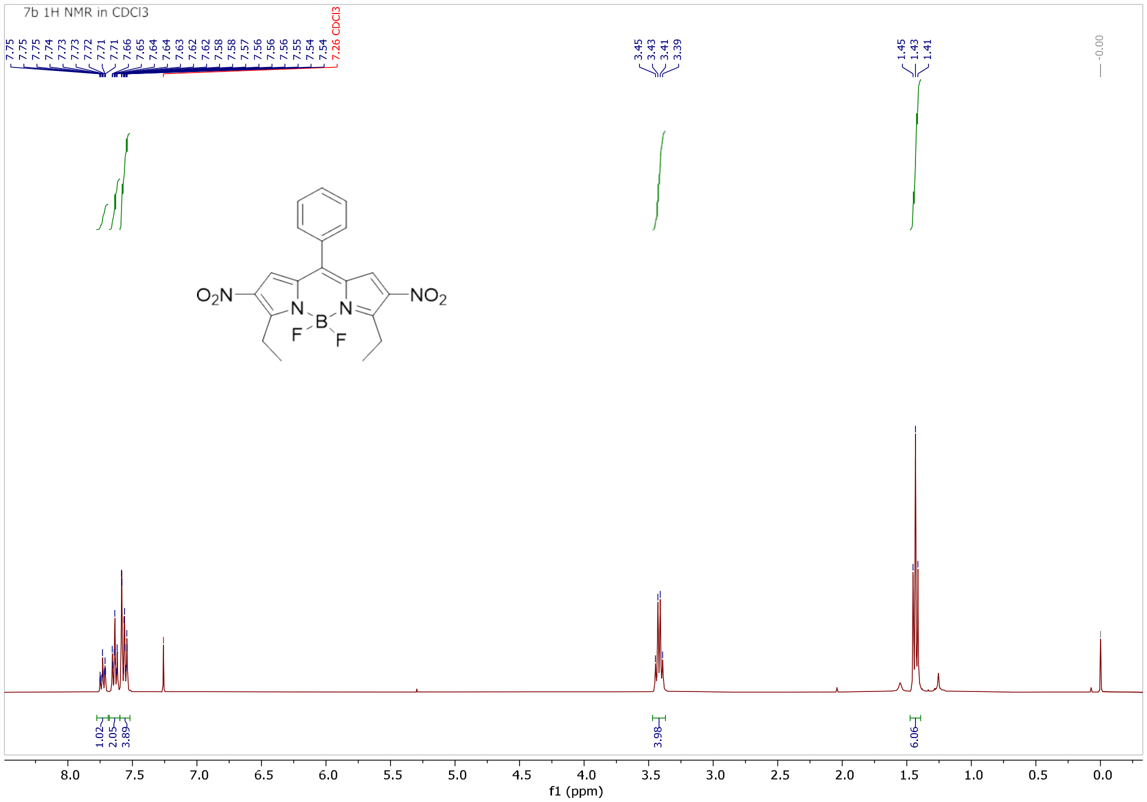

Fig. S46. ^{1}H NMR spectrum of BODIPY **7b** in $CDCl_3$

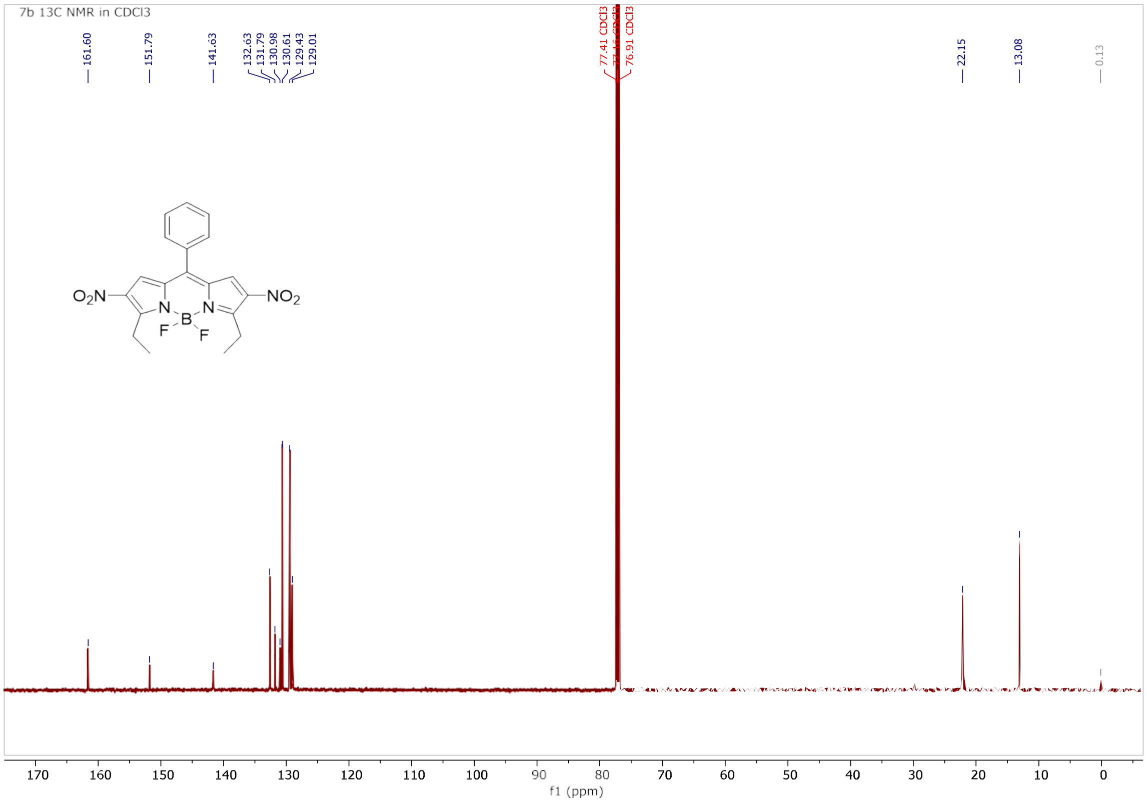

Fig. S47. ^{13}C NMR spectrum BODIPY **7b** in $CDCl_3$

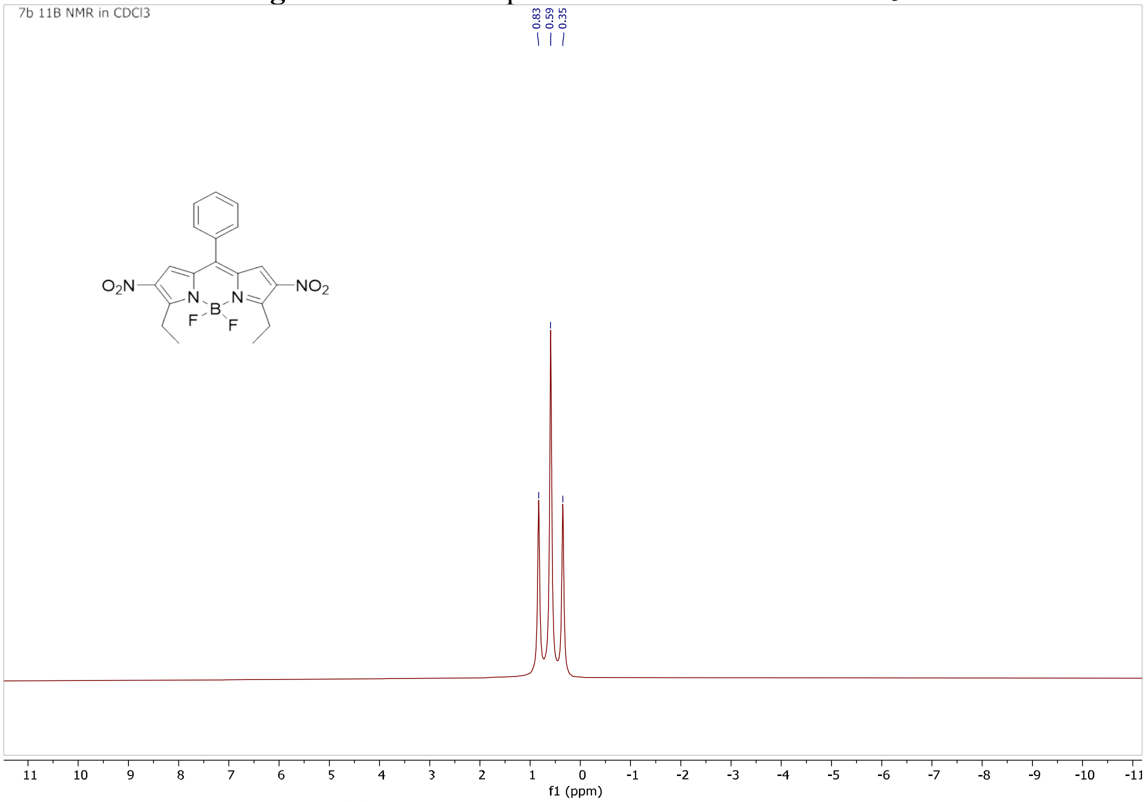

Fig. S48. ^{11}B NMR spectrum of BODIPY **7b** in $CDCl_3$
